# Drug-induced pancreatitis: a real-world analysis of the FDA Adverse Event Reporting System and network pharmacology

**DOI:** 10.3389/fphar.2025.1564127

**Published:** 2025-04-16

**Authors:** Hao Xie, Lin Jiang, Junya Peng, Haoyang Hu, Meifen Han, Bin Zhao

**Affiliations:** ^1^ Department of Pharmacy, Peking Union Medical College Hospital, Chinese Academy of Medical Science and Peking Union Medical College, Beijing, China; ^2^ State Key Laboratory of Complex Severe and Rare Diseases, Peking Union Medical College Hospital, Chinese Academy of Medical Science and Peking Union Medical College, Beijing, China; ^3^ Department of Internal Medicine, Peking Union Medical College Hospital, Chinese Academy of Medical Science and Peking Union Medical College, Beijing, China; ^4^ Department of General Surgery, Peking Union Medical College Hospital, Chinese Academy of Medical Science and Peking Union Medical College, Beijing, China; ^5^ West China School of Pharmacy, Sichuan University, Chengdu, China; ^6^ Department of Pharmacy, Shanghai Children’s Medical Center, School of Medicine, Shanghai Jiao Tong University, Shanghai, China

**Keywords:** pancreatitis, adverse drug reaction, time to onset (TTO), toxicology, target

## Abstract

**Background:**

Drug-induced pancreatitis is a rare disease but frequently reported, owing to the vast number of medications.

**Aim:**

To summarize potential drugs causing pancreatitis and to speculate on underlying mechanisms.

**Methods:**

We extracted more than 60,000 reports of pancreatitis submitted to the U.S. Food and Drug Administration Adverse Event Reporting System (January 2004 to March 2023). Data on patient age, sex, weight, time to onset, and outcome (death et al.) were collected. Disproportionality analysis was used in data mining to identify associations between drugs and pancreatitis events. Seven databases, commonly used for network pharmacology analysis, were searched to identify potential targets.

**Results:**

Of 867 drugs with 3 or more reports, 101 drugs met all criteria using disproportionality analysis and indicated a potential risk of pancreatitis. The risk of 40 drugs had not been previously noted in “UpToDate” database. Patients taking the drugs had a similar sex distribution, were mostly 45–64 years old, and were heavier (median, 88 kg; P < 0.0001). The median time to onset was 199 days (interquartile range, 27–731.5). Ponatinib (16.48%), tigecycline (14.12%) and valproic acid (13.41%) had higher fatality rates. Potential targets related to pancreatitis were identified in 50 of the 101 drugs.

**Conclusion:**

Clinicians providing the 101 drugs for treatment should stay vigilant to detect pancreatitis early.

## Highlights

• More than 60,000 reports of pancreatitis were suspected for drugs.
• From 867 drugs with reports ≥3, 101 drugs were identified.
• The toxicological mechanisms of 50 of 101 drugs were investigated.

## 1 Introduction

Pancreatitis is the leading cause of gastrointestinal-related hospitalizations, associated with considerable mortality and socioeconomic burden (Peery et al., 2019). Drug-induced pancreatitis is a rare disease (Simons-Linares et al., 2019), but since the first reports of acute pancreatitis caused by chlorthalidone and cortisone in the 1950s, hundreds of different classes of commonly used drugs have been reported to cause pancreatic damage (Hung and Abreu Lanfranco, 2014). The World Health Organization database lists 2,479 episodes suspected to be caused by 525 different drugs between 1968 and 1993 (Lancashire et al., 2003). The study of drugs that may cause pancreatitis helps clinicians understand the characteristics of this rare disease and identify drug-induced pancreatitis and avoid re-administration of potentially harmful drugs.

Although many drugs have been reported, most evidence for the relationship between drugs and pancreatitis is weak, and drug-induced pancreatitis remains poorly understood (Nitsche et al., 2012). Moreover, the underlying toxicological mechanisms of drug-induced pancreatitis remain little understood (Nitsche et al., 2010; Simons-Linares et al., 2019). Possible mechanisms include direct toxic effects, accumulation of toxic metabolites or intermediates, immune response, and hypersensitivity reactions. To date, researchers have studied the correlation between the following drugs and pancreatitis using the U.S. Food and Drug Administration Adverse Event Reporting System (FAERS) database, but not systematically and comprehensively: protease inhibitors (Qin et al., 2021), tocilizumab (Kamath et al., 2021), sodium glucose co-transporter 2 inhibitors (Frent et al., 2021), glucocorticoids (Nango et al., 2019), blinatumomab (Vakharia et al., 2018), eluxadoline (Gawron and Bielefeldt, 2018; Harinstein et al., 2018; Cash et al., 2021), tocilizumab (Flaig et al., 2016), and atypical antipsychotics (Hauben, 2004). No researchers have yet conducted network pharmacology analysis combined with real-world pharmacovigilance research on drug-induced pancreatitis.

**FIGURE 1:**
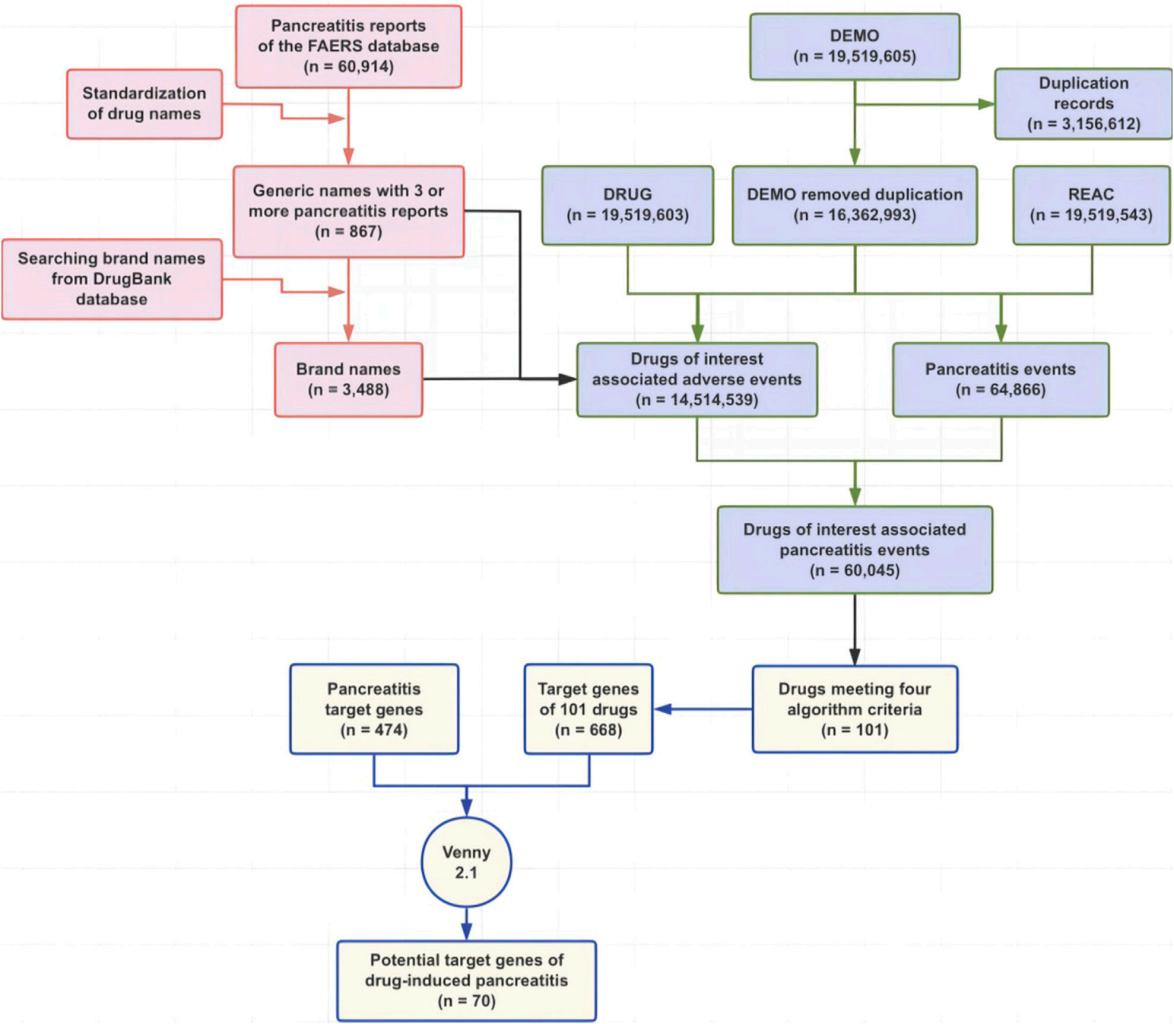
Flowchart of this study. Abbreviations: FAERS, Food and Drug Administration Adverse Event Reporting System.

This study was a combination of real-world pharmacovigilance research based on the FAERS database and network pharmacology (Figure 1) and aimed to summarize potential drugs that can cause pancreatitis and to speculate on the underlying mechanism of drug-induced pancreatitis by analyzing reports of pancreatitis associated with drugs, and targets for drugs and pancreatitis.

## 2 Methods

### 2.1 Adverse event identification

Pancreatitis was standardized using the preferred term (PT) of the International Conference on Harmonization Medical Dictionary for Regular Activities (version 25.0). The following PTs were selected as pancreatitis: “Pancreatitis (10033645),” “Subacute pancreatitis (10084554),” “Pancreatitis viral (10065192),” “Pancreatitis relapsing (10033657),” “Pancreatitis necrotizing (10033654),” “Pancreatitis haemorrhagic (10033650),” “Pancreatitis fungal (10065190)”, “Pancreatitis chronic (10033649),” “Pancreatitis bacterial (10065191),” “Pancreatitis acute (10033647),” “Oedematous pancreatitis (10052400),” “Lupus pancreatitis (10067750),” “Ischaemic pancreatitis (10066127),” “Immune-mediated pancreatitis (10083072),” “Haemorrhagic necrotic pancreatitis (10076058),” “Cytomegalovirus pancreatitis (10049566),” “Autoimmune pancreatitis (10069002).”

### 2.2 Drug screening

The FAERS database is a public, voluntary, spontaneous reporting system, containing seven types of datasets. Data deduplication was conducted according to the FDA recommendations (Xie et al., 2025), the latest FDA_DT (date FDA received case) was selected when the CASEIDs (number for identifying a FAERS case) were the same and the higher PRIMARYID (unique number for identifying a FAERS report) was chosen when the CASEID and FDA_DT were the same. We retrieved 60,914 reports of drug-related pancreatitis events from the database (January 2004 to March 2022), using the PTs described above. Drug names in the resulting reports were manually standardized to generic names using the DrugBank database (https://go.drugbank.com), which was used as a dictionary for drug generic names and brand name data mapping.

### 2.3 Data mining

Reports of pancreatitis events were retrieved from the FAERS database (January 2004 to March 2023) using the generic and brand names as described above. The drugs identified as “primary suspect” in the code for the drug’s reported role in event (ROLE_COD) field of the DRUG files were studied. Based on the basic principles of disproportionality analysis, the reporting odds ratio, proportional reporting ratio, Bayesian confidence propagation neural network, and multi-item gamma Poisson shrinker algorithms were used to investigate the association between the drugs and the pancreatitis event. The equations and criteria for the four algorithms (Candore et al., 2015) are listed in Supplementary Table S1. Data deduplication was conducted according to the U.S. Food and Drug Administration (FDA) recommendations (Bian et al., 2021).

The International Drug Anatomical Therapeutic Chemical (ATC) code was used to classify drugs. The time interval between drug initiation and pancreatitis events (time to onset) and the fatality rate were further analyzed, and the former was defined as the interval between EVENT_DT (adverse event onset date) and START_DT (start date of drug administration). Records with incorrect entries or inputs (EVENT_DT earlier than START_DT) were excluded. The latter was calculated as the number of patients for whom the OUTC_COD (code for the outcome) was DE (death) divided by all the patients for whom the outcome was reported.

### 2.4 Network pharmacological analysis

Pancreatitis target genes were obtained from the DisGeNET database (score_gda ≥0.1, https://www.disgenet.org) and the GeneCard database (relevance score ≥10, https://www.genecards.org). Target genes of drug were obtained from the DGIdb database (https://www.dgidb.org) and the DrugBank database. The UniProt database (https://www.uniprot.org) was used for standardization of target gene names. Venny 2.1 software (https://bioinfogp.cnb.csic.es/tools/venny/) was used to obtain intersecting target genes. The STRING database (https://cn.string-db.org/cgi/input.pl) was used to analyze the protein-protein interactions (PPI) of the target genes. The DAVID database (https://david.ncifcrf.gov) was used for gene ontology (GO) and Kyoto Encyclopedia of Genes and Genomes (KEGG) pathway enrichment analyses. GO analysis included biological process (BP), cellular component (CC), and molecular function (MF).

### 2.5 Statistical analysis

Descriptive analyses were performed to summarize the patients’ demographic features. Continuous variables with normal distribution were described as mean ± standard deviation (x ± s). Non-normally distributed continuous variables were expressed as median [interquartile range (IQR)]. Categorical variables were described as counts (n) and percentages (%). Interval days from drug initiation to onset of pancreatitis events for drugs with the same ATC code in the first four levels and weight were compared using a nonparametric test (the Mann–Whitney U test for dichotomous variables and the Kruskal–Wallis test for more than two subgroups of respondents). Pearson’s chi-squared test or Fisher’s exact test was used to compare sex differences and fatality rate among drugs with the same ATC code in the first four levels. In addition, Pearson’s chi-squared test or Fisher’s exact test was also used to compare the fatality rate of specific drugs with the overall fatality rate of all other drugs. P < 0.05 with 95% confidence intervals (CI) was considered statistically significant. Statistical analyses were performed using SPSS (version 16.0; SPSS Inc., Chicago, IL, United States).

## 3 Results

### 3.1 Disproportionality analysis

**FIGURE 2:**
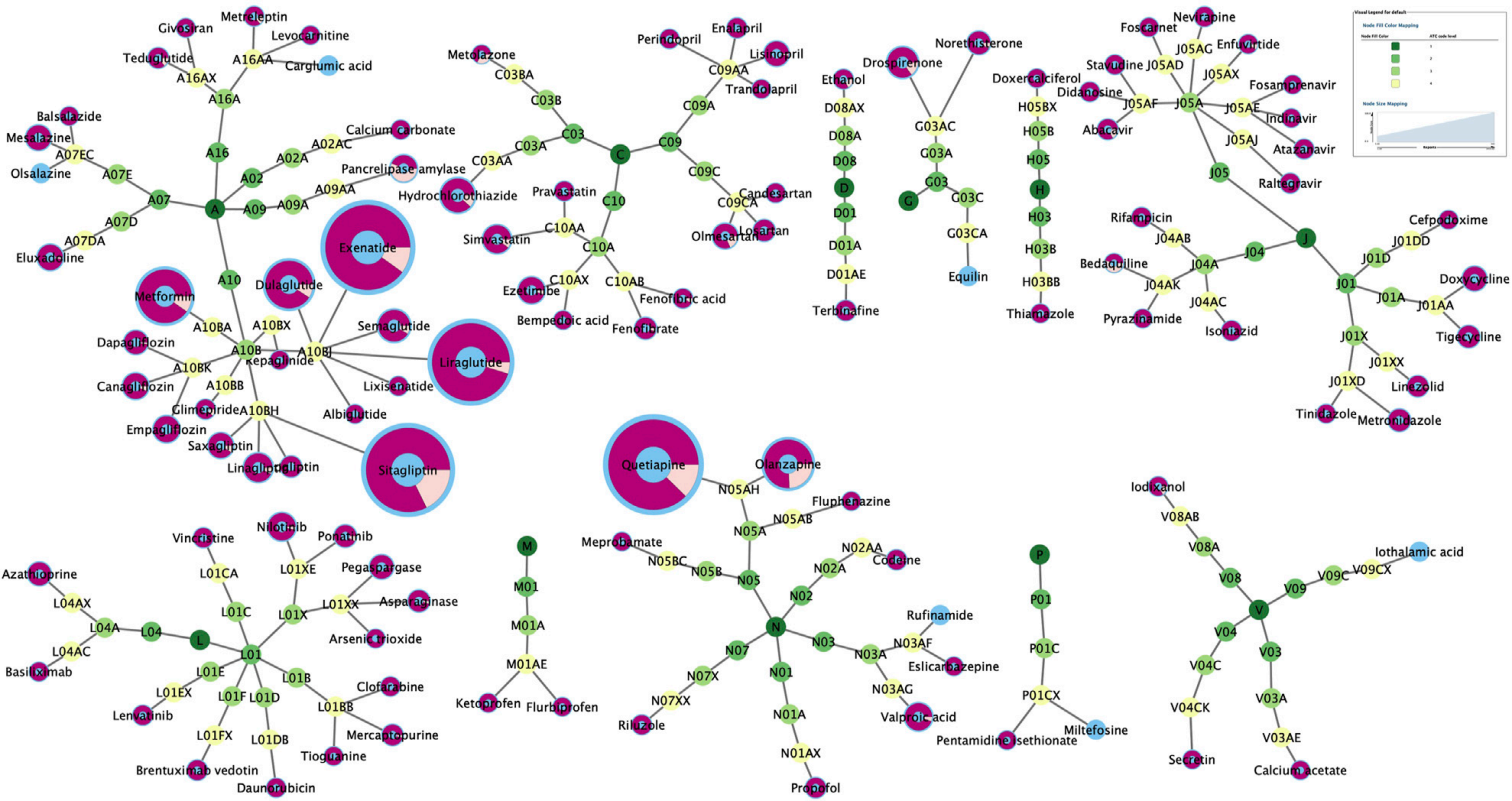
The classification of 101 drugs and proportion of acute pancreatitis (purple) and chronic pancreatitis (pale yellow).

There were 101 drugs that met all four criteria using four algorithms and 40 drugs whose “Drug Information” in the UpToDate database did not mention pancreatitis in “Adverse Reactions” (Table 1). Their ATC codes and proportion of acute pancreatitis and chronic pancreatitis events are shown in Figure 2 and Supplementary Table S2. The 101 drugs included 23 anti-infectives (ten antibacterial, ten antiviral, and three antifungal), 16 hypoglycemic drugs, and 14 antineoplastic drugs. The number of acute pancreatitis was significantly more than that of chronic pancreatitis.

**TABLE 1:** Association of 101 complete signaling drugs with pancreatitis events.

Hypoglycemic, hypotensive, lipid-lowering and anti-infective drugs				Antineoplastic drugs and other drugs			
Drugs	ATC code	Reports (n)	ROR (95% two-sided CI)	Drugs	ATC code	Reports (n)	ROR (95% two-sided CI)
Metformin^a^	A10BA	1700	5.53 (5.27, 5.81)	Mercaptopurine	L01BB	128	14.62 (12.23, 17.48)
Glimepiride^a^	A10BB	63	5.36 (4.17, 6.88)	Clofarabine	L01BB	41	4.40 (3.23, 5.99)
Sitagliptin	A10BH	3,167	26.45 (25.48, 27.46)	Tioguanine^a^	L01BB	6	4.66 (2.08, 10.45)
Linagliptin	A10BH	501	16.35 (14.93, 17.90)	Vincristine^a^	L01CA	71	2.72 (2.15, 3.43)
Saxagliptin	A10BH	330	16.55 (14.80, 18.50)	Daunorubicin^a^	L01DB	27	3.28 (2.25, 4.80)
Alogliptin	A10BH	135	22.79 (19.10, 27.19)	Lenvatinib	L01EX	70	4.40 (3.48, 5.58)
Exenatide	A10BJ	3,210	11.62 (11.21, 12.05)	Brentuximab vedotin	L01FX	89	3.78 (3.06, 4.66)
Liraglutide	A10BJ	2,865	25.37 (24.40, 26.38)	Nilotinib	L01XE	448	4.91 (4.47, 5.39)
Dulaglutide	A10BJ	1,415	6.47 (6.14, 6.83)	Ponatinib	L01XE	182	5.89 (5.08, 6.82)
Semaglutide	A10BJ	650	9.54 (8.81, 10.32)	Pegaspargase	L01XX	267	18.90 (16.69, 21.41)
Lixisenatide	A10BJ	36	4.38 (3.15, 6.10)	Asparaginase	L01XX	163	30.15 (25.62, 35.49)
Albiglutide	A10BJ	7	14.56 (6.79, 31.22)	Arsenic trioxide^a^	L01XX	14	3.14 (1.85, 5.32)
Empagliflozin	A10BK	381	3.71 (3.35, 4.10)	Basiliximab^a^	L04AC	21	3.52 (2.29, 5.41)
Canagliflozin	A10BK	360	3.90 (3.51, 4.32)	Azathioprine	L04AX	238	11.70 (10.27, 13.33)
Dapagliflozin	A10BK	259	3.67 (3.24, 4.15)	Eluxadoline	A07DA	350	47.34 (42.22, 53.09)
Repaglinide	A10BX	12	3.69 (2.08, 6.52)	Metreleptin^a^	A16AA	52	15.10 (11.41, 19.98)
Hydrochlorothiazide	C03AA	752	2.53 (2.36, 2.72)	Carglumic acid	A16AA	16	8.87 (5.38, 14.60)
Metolazone	C03BA	11	7.32 (4.02, 13.33)	Levocarnitine^a^	A16AA	4	5.55 (2.06, 14.94)
Lisinopril	C09AA	306	2.61 (2.33, 2.92)	Teduglutide	A16AX	101	2.69 (2.21, 3.28)
Enalapril	C09AA	69	3.48 (2.75, 4.42)	Givosiran^a^	A16AX	13	6.78 (3.90, 11.76)
Perindopril^a^	C09AA	40	3.72 (2.73, 5.09)	Mesalazine	A07EC	319	9.05 (8.09, 10.12)
Trandolapril	C09AA	14	3.49 (2.06, 5.91)	Balsalazide	A07EC	9	7.59 (3.91, 14.74)
Olmesartan^a^	C09CA	319	4.36 (3.90, 4.87)	Olsalazine	A07EC	3	26.30 (8.00, 86.51)
Losartan^a^	C09CA	158	2.33 (1.99, 2.72)	Ketoprofen	M01AE	17	3.83 (2.37, 6.18)
Candesartan^a^	C09CA	41	3.23 (2.37, 4.39)	Flurbiprofen^a^	M01AE	5	7.77 (3.19, 18.92)
Simvastatin	C10AA	472	2.88 (2.63, 3.16)	Drospirenone^a^	G03AC	589	3.39 (3.12, 3.68)
Pravastatin	C10AA	51	2.90 (2.20, 3.82)	Norethisterone^a^	G03AC	226	3.00 (2.63, 3.42)
Fenofibrate	C10AB	158	8.99 (7.67, 10.54)	Equilin^a^	G03CA	3	19.38 (5.98, 62.78)
Fenofibric acid	C10AB	51	6.54 (4.95, 8.63)	Thiamazole	H03BB	9	5.40 (2.79, 10.46)
Ezetimibe	C10AX	387	6.75 (6.10, 7.47)	Doxercalciferol^a^	H05BX	9	6.09 (3.14, 11.79)
Bempedoic acid^a^	C10AX	12	3.38 (1.91, 5.98)	Propofol	N01AX	92	3.30 (2.69, 4.05)
Terbinafine	D01AE	88	2.70 (2.19, 3.33)	Codeine	N02AA	77	2.84 (2.27, 3.55)
Doxycycline^a^	J01AA	285	4.99 (4.44, 5.62)	Eslicarbazepine^a^	N03AF	8	11.29 (5.56, 22.93)
Tigecycline	J01AA	179	23.28 (19.97, 27.14)	Rufinamide^a^	N03AF	6	4.77 (2.12, 10.69)
Cefpodoxime^a^	J01DD	9	8.24 (4.24, 16.02)	Valproic acid	N03AG	440	6.21 (5.65, 6.83)
Metronidazole	J01XD	180	2.82 (2.43, 3.27)	Fluphenazine^a^	N05AB	7	5.69 (2.69, 12.04)
Tinidazole^a^	J01XD	4	15.58 (5.67, 42.82)	Quetiapine	N05AH	3459	13.38 (12.92, 13.86)
Linezolid^a^	J01XX	128	2.74 (2.30, 3.26)	Olanzapine	N05AH	1459	8.57 (8.13, 9.03)
Rifampicin^a^	J04AB	59	3.67 (2.84, 4.74)	Meprobamate^a^	N05BC	3	7.67 (2.43, 24.21)
Isoniazid	J04AC	70	6.36 (5.01, 8.06)	Riluzole	N07XX	59	12.36 (9.51, 16.05)
Bedaquiline^a^	J04AK	23	3.24 (2.15, 4.89)	Pancrelipase amylase^a^	A09AA	400	10.98 (9.93, 12.14)
Pyrazinamide^a^	J04AK	11	5.17 (2.85, 9.40)	Secretin^a^	V04CK	4	245.46 (61.39, 981.52)
Foscarnet	J05AD	11	3.63 (2.00, 6.58)	Calcium carbonate^a^	A02AC	31	2.95 (2.07, 4.20)
Atazanavir	J05AE	57	3.26 (2.51, 4.24)	Ethanol	D08AX	57	3.48 (2.68, 4.52)
Fosamprenavir^a^	J05AE	10	4.52 (2.42, 8.45)	Calcium acetate^a^	V03AE	9	8.80 (4.53, 17.12)
Indinavir	J05AE	8	5.05 (2.51, 10.17)	Iodixanol	V08AB	35	4.28 (3.06, 5.98)
Abacavir	J05AF	101	2.48 (2.04, 3.02)	Iothalamic acid^a^	V09CX	7	20.96 (9.68, 45.34)
Didanosine	J05AF	51	22.94 (17.22, 30.57)				
Stavudine	J05AF	51	10.96 (8.28, 14.51)				
Nevirapine^a^	J05AG	66	2.57 (2.02, 3.28)				
Raltegravir^a^	J05AJ	65	3.07 (2.40, 3.92)				
Enfuvirtide	J05AX	27	5.32 (3.64, 7.80)				
Pentamidine isethionate	P01CX	6	12.07 (5.32, 27.40)				
Miltefosine^a^	P01CX	3	10.52 (3.31, 33.41)				

^a^ Pancreatitis was not mentioned in “Adverse Reactions” of “Drug Information” in “UpToDate” database.

Abbreviations: ROR, reporting odds ratio; CI, confidence interval; PRR, proportional reporting ratio; χ2, chi-squared; IC, information component; IC025, the lower limit of the 95% two-sided CI of the IC; EBGM, empirical Bayesian geometric mean; EBGM05, the lower 90% one-sided CI of EBG.

Antipsychotics (quetiapine and olanzapine), glucagon-like peptide-1 (GLP-1) analogs (exenatide, liraglutide, dulaglutide), dipeptidyl peptidase 4 (DPP-4) inhibitors (sitagliptin), and metformin were the most commonly reported (59.43%). Notably, many drugs with few reports but a potential association with pancreatitis were found, for which the risk of pancreatitis had not previously been perceived.

### 3.2 Overall demographic characteristics

Demographic information was obtained from 67,574 reports of pancreatitis events for 867 drugs (summarized in Table 2). Complete signaling drugs were defined as those that met all four criteria using the four algorithms. Partial signaling drugs met any one to three of the four criteria (Supplementary Table S3). Complete non-signaling drugs did not meet any of the four criteria.

**TABLE 2:** Demographic characteristics of patients with drug-associated pancreatitis events sourced from the FAERS database (January 2004 to March 2023).

Characteristics	Category	Reports (n, %)			
		All (67,574, 100%)	Complete signaling drugs (29,067, 43.02%)	Partial signaling drugs (16,616, 24.59%)	Complete non-signaling drugs (21,891, 32.40%)
Age	<18 years	2,511 (3.72)	712 (2.45)	924 (5.56)	875 (4.00)
	18–44 years	10,928 (16.17)	4,140 (14.24)	3,145 (18.93)	3,643 (16.64)
	45–64 years	18,829 (27.86)	7,920 (27.25)	4,551 (27.39)	6,358 (29.04)
	65–74 years	7,754 (11.47)	3,021 (10.39)	2,222 (13.37)	2,511 (11.47)
	75–84 years	3,634 (5.38)	1,213 (4.17)	1,057 (6.36)	1,364 (6.23)
	≥85 years	875 (1.29)	280 (0.96)	218 (1.31)	377 (1.72)
	Unknown	23,043 (34.10)	11,781 (40.53)	4,499 (27.08)	6,763 (30.89)
Gender	Female	32,566 (48.19)	12,939 (44.51)	7,818 (47.05)	11,809 (53.94)
	Male	28,238 (41.79)	12,591 (43.32)	7,373 (44.37)	8,274 (37.80)
	Unknown	6,770 (10.02)	3,537 (12.17)	1,425 (8.58)	1,808 (8.26)
Weight (kg)	Mean	82.11	90.27	74.51	75.34
	Median (interquartile range)	80.00 (65.00–97.00)	88.00 (73.00–106.00)	73.00 (60.00–87.00)	73.00 (60.00–88.00)
	Unknown	45,551 (67.41)	18,789 (64.64)	11,539 (69.45)	15,223 (69.54)
Reporting region	Africa	215 (0.32)	65 (0.22)	73 (0.44)	77 (0.35)
	Asian	4,032 (5.97)	968 (3.33)	1,662 (10.00)	1,402 (6.40)
	Europe	14,999 (22.20)	4,182 (14.39)	4,737 (28.51)	6,080 (27.77)
	North America	44,438 (65.76)	22,676 (78.01)	8,978 (54.03)	12,784 (58.40)
	Oceania	740 (1.10)	231 (0.79)	271 (1.63)	238 (1.09)
	South America	1,123 (1.66)	273 (0.94)	283 (1.70)	567 (2.59)
	Unknown	2,027 (3.00)	672 (2.31)	612 (3.68)	743 (3.39)

Excluding reports of unknown age, 66.82% of all pancreatitis events occurred in the 18–64 years age group and only 5.64% occurred in the <18 years age group. Excluding reports of unknown sex, pancreatitis events with signaling drugs (female: complete 50.68%, partial 51.46%) were more evenly distributed between sexes than those with non-signaling drugs (female: 58.80%). Excluding reports of unknown weight, patients with pancreatitis events with complete signaling drugs (median, 88 kg) were heavier (P < 0.0001). More than half of the events occurred in North America, followed by Europe, which may be related to the data sources.

### 3.3 Demographic characteristics of specific drugs

Detailed demographic information on the 101 signaling drugs is summarized in Supplementary Table S4. Pancreatitis events occurred more in the 0–18 years age group for asparaginase (85/109), carglumic acid (10/11), clofarabine (20/38), and vincristine (39/58). The following drugs were reported in significantly more female patients: doxycycline (174:87), drospirenone (582:3), eluxadoline (258:57), liraglutide (1401:1111), metreleptin (45:5), and quetiapine (1822:1490). These drugs were reported in significantly more male patients (P < 0.0001): alogliptin (34:84), atazanavir (11:40), lisinopril (83:204), sitagliptin (1216:1493), and terbinafine (23:58).

### 3.4 Time to onset and fatality rate

**FIGURE 3:**
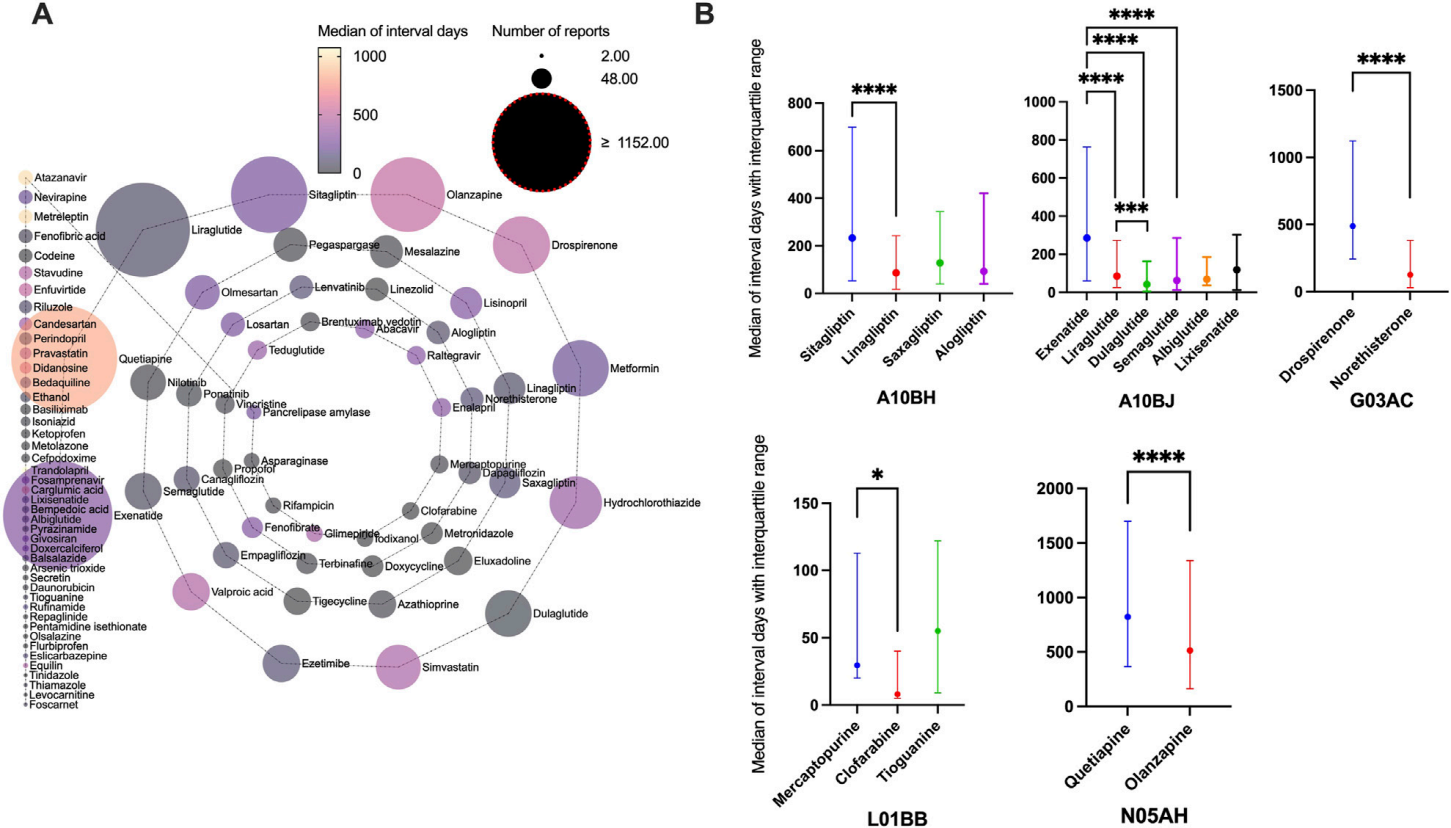
Time interval between drug initiation and pancreatitis event. **(A)** Median of interval days from drug initiation to onset of pancreatitis event for different drugs. **(B)** Drugs with the same Anatomical Therapeutic Chemical code in the first four levels with significant differences in interval days.

Complete information on interval days from drug initiation to the onset of pancreatitis was available for 94 of the 101 drugs, and the distribution of days between was right-skewed (Supplementary Table S5). The median was 199 days (IQR: 27–731.5). More than half of the drugs were administered within a median of 3 months (50/94), approximately two-thirds within 6 months (61/94), and most within a year (78/94). Drugs with the same ATC code in the first four levels with significant differences in interval days are shown in Figure 3. The following drugs showed significantly later onset of pancreatitis than similar drugs: sitagliptin, exenatide, drospirenone, mercaptopurine, and quetiapine. Additionally, time to onset of 20 drugs with valid report number ≥100 is summarized in Table 3, showing a clear distinction between different classes of drugs. Quetiapine (823 days), olanzapine (514 days), drospirenone (488 days), valproic acid (437 days), and simvastatin (434 days) are classified as ultra-long latency drugs (median >1 year), suggesting potential associations with the cumulative effects of long-term medication use. GLP-1 analogs: liraglutide (85 days), semaglutide (63 days), dulaglutide (42 days), along with mesalazine (27 days), pegaspargase (23 days), and nilotinib (13.5 days), are categorized as short-latency drugs (median <3 months), indicating possible acute drug reactions. Notably, pegaspargase and nilotinib, as chemotherapeutic/immunomodulatory agents, exhibit ultra-short latency, likely linked to their direct cytotoxic effects. Lisinopril (IQR: 59–2006 days) and valproic acid (IQR: 89–1712 days) demonstrate extremely wide IQR spans, reflecting significant inter-individual variability and suggesting the potential for idiosyncratic reactions in high-risk populations.

**TABLE 3:** Time to onset of pancreatitis of drugs with valid report number ≥100.

Drugs	Reports (n)	Median of interval days	Interquartile range of interval days
Quetiapine	1,308	823	365–1,698.5
Olanzapine	646	514	162.75–1,339.5
Drospirenone	381	488	243–1,122.5
Valproic acid	163	437	89–1712
Simvastatin	234	434	69.75–1,253
Hydrochlorothiazide	321	359	61.5–1,032
Exenatide	1,390	285.5	60–763.25
Lisinopril	119	270	59–2,006
Olmesartan	140	233.5	30.25–676
Sitagliptin	678	233	53.25–699
Metformin	367	202	47–587
Saxagliptin	113	128	39.5–344
Ezetimibe	164	107.5	27–644
Linagliptin	118	86.5	17–242.75
Liraglutide	1,032	85	25–273
Semaglutide	159	63	12–285
Dulaglutide	252	42	5–163
Mesalazine	126	27	9–124
Pegaspargase	135	23	14–54
Nilotinib	148	13.5	4–182

**FIGURE 4:**
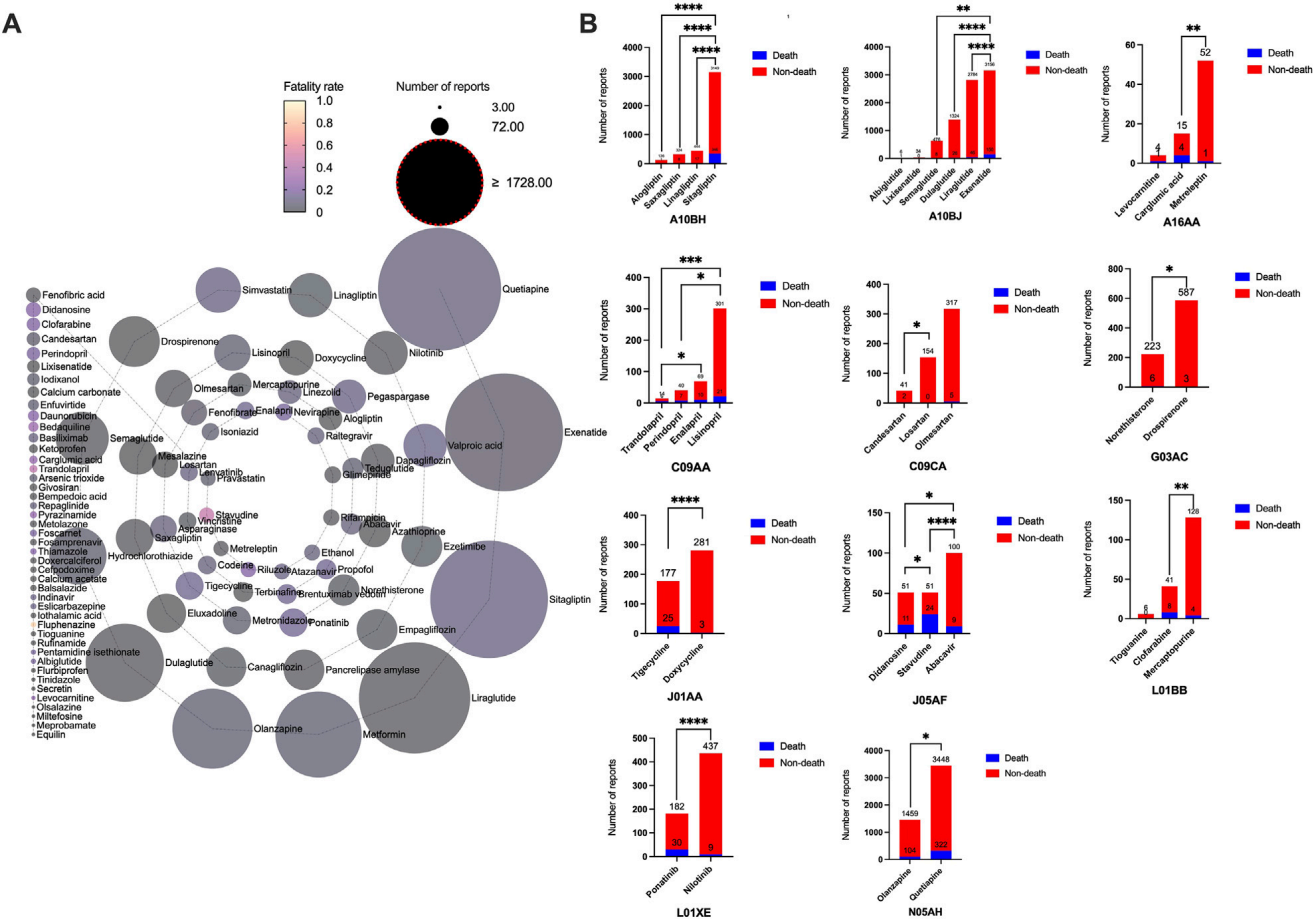
Outcomes of patients with drug-associated pancreatitis. **(A)** Fatality rate due to different categories of drugs associated pancreatitis events. **(B)** Drugs with the same Anatomical Therapeutic Chemical code in the first four levels with significant differences in death and non-death.

The outcomes of patients with drug-associated pancreatitis related to these 101 drugs (Supplementary Table S6) included congenital anomaly, death, disability et al. Drugs with the same ATC code in the first four levels, with significant differences between death and non-death, are shown in Figure 4. The following drugs showed significantly higher fatality rates than similar drugs: sitagliptin, exenatide, carglumic acid, trandolapril, candesartan, norethisterone, tigecycline, stavudine, clofarabine, ponatinib, and quetiapine. Outcomes in patients of drugs with valid report number ≥100 is summarized in Table 4. Ponatinib (16.48%, P < 0.0001), tigecycline (14.12%, P = 0.0001) and valproic acid (13.41%, P < 0.0001) had higher fatality rates. Losartan (0/154) and mesalazine (0/318) had zero fatality rates.

**TABLE 4:** Outcomes in patients of pancreatitis of drugs with valid report number ≥100.

Drugs	Total reports (n)	Death reports (n)	Fatality rate (%)
Ponatinib	182	30	16.48
Tigecycline	177	25	14.12
Valproic acid	425	57	13.41
Pegaspargase	263	31	11.79
Simvastatin	468	53	11.32
Sitagliptin	3,149	346	10.99
Linezolid	127	12	9.45
Quetiapine	3,448	322	9.34
Abacavir	100	9	9.00
Asparaginase	162	14	8.64
Olanzapine	1,459	104	7.13
Lisinopril	301	21	6.98
Teduglutide	101	7	6.93
Metformin	1,689	113	6.69
Hydrochlorothiazide	744	48	6.45
Metronidazole	179	10	5.59
Fenofibrate	152	8	5.26
Exenatide	3,162	150	4.74
Ezetimibe	381	15	3.94
Linagliptin	444	17	3.83
Canagliflozin	359	12	3.34
Mercaptopurine	128	4	3.13
Norethisterone	223	6	2.69
Empagliflozin	378	10	2.65
Saxagliptin	324	8	2.47
Pancrelipase amylase	375	8	2.13
Dulaglutide	1,392	29	2.08
Nilotinib	437	9	2.06
Semaglutide	638	13	2.04
Liraglutide	2,816	46	1.63
Olmesartan	317	5	1.58
Dapagliflozin	254	4	1.57
Eluxadoline	350	5	1.43
Azathioprine	230	3	1.30
Doxycycline	281	3	1.07
Alogliptin	126	1	0.79
Drospirenone	587	3	0.51
Losartan	154	0	0.00
Mesalazine	318	0	0.00

### 3.5 Target gene analysis

**FIGURE 5:**
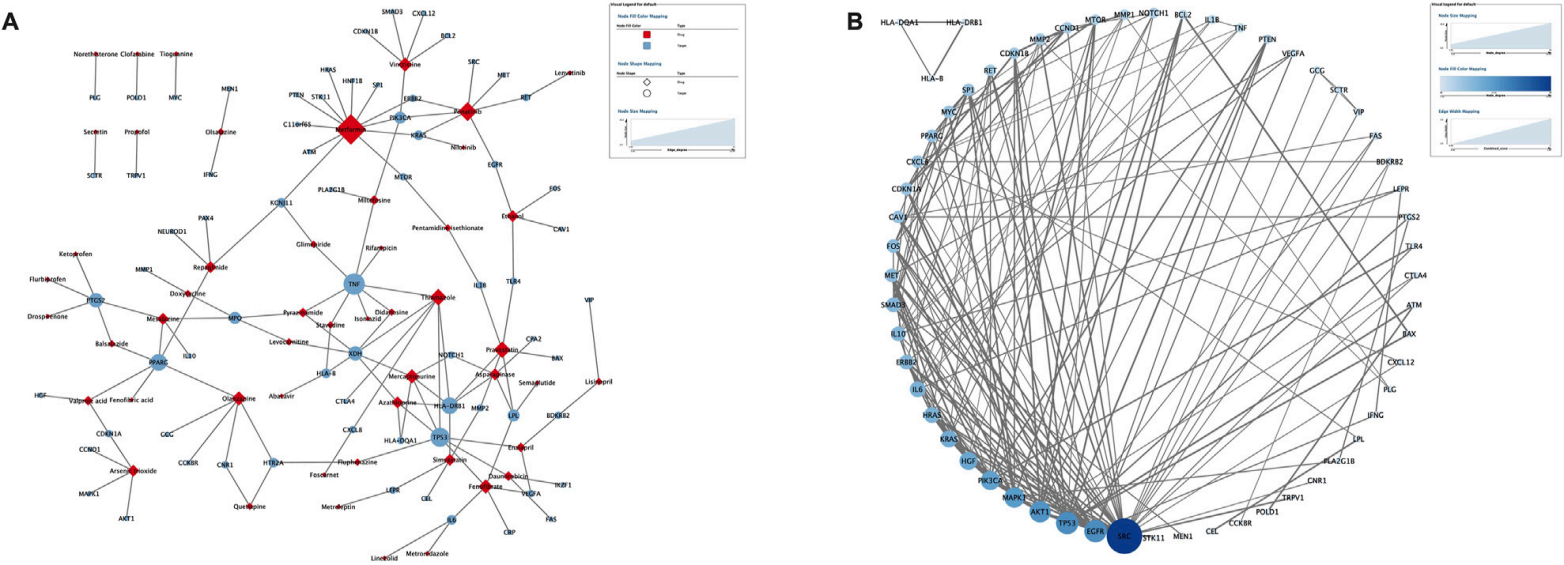
Potential target genes of drug-induced pancreatitis. **(A)** The drug-target network map of 50 drugs and 70 target genes. **(B)** The protein-protein interaction network from STRING database map (known interactions).

The DisGeNET and GeneCard databases were searched, yielding 474 pancreatitis target genes, excluding duplicates. Similarly, 1713 target genes were obtained from the DGIdb and DrugBank databases for the 101 complete signaling drugs. Removal of duplicates after verification yielded 668 target genes. The target genes were intersected using Venny 2.1 software to obtain 70 potential targets (Table 5). Full names of the 70 target genes were showed in Supplementary Table S7. Potential target genes for 50 of 101 drugs were obtained (Figure 5A). Sequentially, the PPI network map and the most interacting target genes of the 70 were obtained using the STRING database (Figure 5B). The following target genes had more interactions: SRC (28), EGFR (16), TP53 (16), AKT1 (15), MAPK1 (14), PIK3CA (13), HGF (12), KRAS (11), HRAS (10), IL (10).

**TABLE 5:** Potential target genes of drug-induced pancreatitis, number of interactions and drugs related to the target.

Target genes	Node_degree	Drugs	Target genes	Node_degree	Drugs	Target genes	Node_degree	Drugs
SRC	28	Ponatinib	MMP2	6	Pravastatin	HLA-DRB1	2	Asparaginase
EGFR	16	Ethanol	MMP2	6	Simvastatin	HLA-DRB1	2	Azathioprine
EGFR	16	Ponatinib	RET	6	Lenvatinib	HLA-DRB1	2	Mercaptopurine
TP53	16	Azathioprine	RET	6	Ponatinib	HLA-DRB1	2	Pravastatin
TP53	16	Daunorubicin	MMP1	5	Doxycycline	HLA-DRB1	2	Simvastatin
TP53	16	Enalapril	MTOR	5	Metformin	HLA-DRB1	2	Thiamazole
TP53	16	Fenofibrate	MTOR	5	Pentamidine isethionate	IFNG	2	Olsalazine
TP53	16	Fluphenazine	NOTCH1	5	Asparaginase	LPL	2	Asparaginase
TP53	16	Mercaptopurine	NOTCH1	5	Mercaptopurine	LPL	2	Fenofibrate
TP53	16	Thiamazole	BCL2	4	Vincristine	LPL	2	Pravastatin
AKT1	15	Arsenic trioxide	IL1B	4	Pentamidine isethionate	LPL	2	Semaglutide
MAPK1	14	Arsenic trioxide	IL1B	4	Pravastatin	PLA2G1B	2	Miltefosine
PIK3CA	13	Metformin	PTEN	4	Metformin	PLG	2	Norethisterone
PIK3CA	13	Miltefosine	TNF	4	Didanosine	TLR4	2	Ethanol
PIK3CA	13	Ponatinib	TNF	4	Glimepiride	TLR4	2	Pravastatin
PIK3CA	13	Vincristine	TNF	4	Isoniazid	CCKBR	1	Olanzapine
HGF	12	Valproic acid	TNF	4	Miltefosine	CEL	1	Simvastatin
KRAS	11	Metformin	TNF	4	Pyrazinamide	CNR1	1	Olanzapine
KRAS	11	Nilotinib	TNF	4	Rifampicin	CNR1	1	Quetiapine
KRAS	11	Ponatinib	TNF	4	Stavudine	MEN1	1	Olsalazine
HRAS	10	Metformin	TNF	4	Thiamazole	POLD1	1	Clofarabine
IL6	10	Fenofibrate	BDKRB2	3	Enalapril	STK11	1	Metformin
IL6	10	Linezolid	BDKRB2	3	Lisinopril	TRPV1	1	Propofol
IL6	10	Metronidazole	FAS	3	Daunorubicin	C11orf65	0	Metformin
ERBB2	9	Metformin	GCG	3	Olanzapine	CPA2	0	Asparaginase
ERBB2	9	Ponatinib	LEPR	3	Metreleptin	CRP	0	Fenofibrate
IL10	9	Mesalazine	LEPR	3	Simvastatin	HNF1B	0	Metformin
SMAD3	9	Vincristine	PTGS2	3	Balsalazide	HTR2A	0	Fluphenazine
FOS	8	Ethanol	PTGS2	3	Drospirenone	HTR2A	0	Olanzapine
MET	8	Ponatinib	PTGS2	3	Flurbiprofen	HTR2A	0	Quetiapine
CAV1	7	Ethanol	PTGS2	3	Ketoprofen	IKZF1	0	Daunorubicin
CDKN1A	7	Arsenic trioxide	PTGS2	3	Mesalazine	KCNJ11	0	Glimepiride
CDKN1A	7	Valproic acid	SCTR	3	Secretin	KCNJ11	0	Metformin
CXCL8	7	Foscarnet	VEGFA	3	Enalapril	KCNJ11	0	Repaglinide
CXCL8	7	Thiamazole	VEGFA	3	Fenofibrate	MPO	0	Doxycycline
MYC	7	Tioguanine	VIP	3	Lisinopril	MPO	0	Levocarnitine
PPARG	7	Balsalazide	ATM	2	Metformin	MPO	0	Mesalazine
PPARG	7	Fenofibric acid	BAX	2	Pravastatin	MPO	0	Pyrazinamide
PPARG	7	Mesalazine	CTLA4	2	Thiamazole	NEUROD1	0	Repaglinide
PPARG	7	Olanzapine	CXCL12	2	Vincristine	PAX4	0	Repaglinide
PPARG	7	Repaglinide	HLA-B	2	Abacavir	XDH	0	Azathioprine
PPARG	7	Valproic acid	HLA-B	2	Stavudine	XDH	0	Didanosine
SP1	7	Metformin	HLA-B	2	Thiamazole	XDH	0	Levocarnitine
CCND1	6	Arsenic trioxide	HLA-DQA1	2	Azathioprine	XDH	0	Mercaptopurine
CDKN1B	6	Vincristine	HLA-DQA1	2	Mercaptopurine	XDH	0	Pyrazinamide

A total of 420 BPs, 41 CCs, 53 MFs, and 154 KEGG pathways were obtained from the GO/KEGG enrichment analysis (P < 0.05). The top 20 most appreciably enriched entities by gene count are shown in Supplementary Figure S1. The toxicological mechanisms of drug-induced pancreatitis may be related to these factors. The KEGG enrichment analysis suggested these drugs mainly work through multiple pathways in pathways in cancer, endocrine resistance, human cytomegalovirus infection, EGFR tyrosine kinase inhibitor resistance, proteoglycans in cancer.

## 4 Discussion

The overall incidence of drug-induced pancreatitis is thought to be between 0.1% and 2% (Balani and Grendell, 2008), accounting for approximately 5% of acute pancreatitis cases (Vinklerová et al., 2010; Lankisch et al., 2015). The low incidence of drug-induced pancreatitis makes prospective studies challenging. We obtained reports of drug-related pancreatitis events from the FAERS database, and comprehensively analyzed the association and demographic information, time to onset, and death reports. Toxicological mechanisms were explored using network pharmacology. Through analysis of the FAERS database, 101 drugs with the risk of pancreatitis were identified, of which 40 drugs had not been reported in the UpToDate database.

Most drug-induced pancreatitis cases (66.82%) occurred in the 18–64 years age group, possibly due to the greater number of drug users in this age group. Pancreatitis events occurred more in the 0–18 years age group for asparaginase, carglumic acid, clofarabine, and vincristine. Asparaginase, clofarabine, and vincristine are commonly used to treat acute lymphoblastic leukemia, the most common malignant tumor in childhood (Ward et al., 2014). Carglumic acid is commonly used to treat hyperammonemia in patients with a deficiency in N-acetyl glutamate synthase (a rare genetic disorder). Enzyme deficiencies of adult patients are mild, and thus carglumic acid is usually used under the age of eighteen (Nashabat et al., 2019). Women are thought to be more susceptible to drug-induced pancreatitis (Balani and Grendell, 2008). However, our demographic results were not strong enough to support this view due to influence by the different numbers of drug users by sex. Pancreatitis events associated with doxycycline, drospirenone, eluxadoline, liraglutide, metreleptin, and quetiapine were more prevalent in women. Doxycycline, drospirenone, liraglutide, and quetiapine are usually used more in women. Eluxadoline is used to treat irritable bowel syndrome (IBS) with diarrhea. Although the overall prevalence of IBS is higher in women than men (prevalence: 14%, 9%) (Lovell and Ford, 2012), the sex ratio of patients does not reach that of patients with pancreatitis. A study of eluxadoline (Cash et al., 2021) found that the proportion of female patients without a gallbladder was higher. Eluxadoline could increase the sphincter of Oddi tone, and absence of the gallbladder could lead to increased pressure in the pancreaticobiliary ductal system, which could potentiate clinical signs and symptoms of pancreatitis (Afghani et al., 2017). Metreleptin is commonly used to treat lipodystrophy, and the dosage used in women is generally greater than in men, possibly accounting for the increased incidence of pancreatitis in women. We found that patients with pancreatitis events with complete signaling drugs (median, 88 kg) were heavier (P < 0.0001). Obesity can increase the risk of pancreatitis and even aggravate the severity of pancreatitis (Khatua et al., 2017).

It is interesting that the hypoglycemic and now anti-obesity drugs (GLP-1 analogs and DDP-4 inhibitors) are significantly associated with pancreatitis (both in number of reports and strength of signal). Long-term overstimulation of GLP-1 receptors in exocrine pancreatic cells increases exocrine pancreatic secretion and could theoretically induce pancreatitis. A population-based matched case-control study found that sitagliptin and exenatide was associated with increased odds of hospitalization for acute pancreatitis (Singh et al., 2013). There are meta-analyses of clinical trials that have reported DDP-4 inhibitors to be associated with an increased risk of pancreatitis (Pinto et al., 2018; Abd El Aziz et al., 2020). No clinical trials have shown the pancreatitis risk of GLP-1 analogs. However, it should be recognized that there is a small number of observed cases of pancreatitis and wide CI of risk estimates. Despite insufficient evidence, the FDA included a label warning about the risk of acute pancreatitis of DDP-4 inhibitors and GLP-1 analogs on the basis of postmarketing data. Risk for pancreatitis in such patients is confounded by diabetes. However, a recent study found that GLP-1 analogs used for weight loss also increase pancreatitis risk (Sodhi et al., 2023). Overall, the evidence in clinical trials supporting the risk of pancreatitis in DPP-4 inhibitors and GLP-1 analogs is weak due to sample size limitations. However, their pancreatitis risk has been preliminarily demonstrated based on the results of post-marketing observational studies. For GLP-1 analogues and DPP-4 inhibitors, a baseline assessment of pancreatic risk factors (e.g., triglyceride level, gallbladder status, alcohol use history) can be performed before initiating therapy, especially in patients with preexisting metabolic syndrome (Tenner et al., 2024). Based on the median incubation period of GLP-1 analogues (42–85 days, excluding exenatide), it is recommended to focus on monitoring serum lipase/amylase during the first 3 months of treatment initiation.

Although there are studies (Singh et al., 2013; Pinto et al., 2018; Abd El Aziz et al., 2020; Dicembrini et al., 2020; Zhang et al., 2022) on whether DPP-4 inhibitors and GLP-1 analogs increase the risk of pancreatitis, few studies have focused on time to onset. We found that sitagliptin-related pancreatitis usually occurred approximately 6 months after drug initiation, while other DPP-4 inhibitors usually occurred at 3 months. The profiles of exenatide relative to those of other GLP-1 analogs were similar. In addition, we summarized the fatality rate of patients. Notably, sitagliptin (fatality rate: 10.99%) and exenatide (fatality rate: 4.79%) related pancreatitis occurred later but might be more severe. Observational studies have reported an increased risk of subclinical pancreatic inflammation and pancreatic cancer in sitagliptin and exenatide users (Cohen, 2013; Halfdanarson and Pannala, 2013; Singh et al., 2013). Chronic pancreatitis has been suggested as a potential mechanism for the development of pancreatic cancer in patients taking incretins (Halfdanarson and Pannala, 2013). Long-term subclinical pancreatitis caused by sitagliptin and exenatide is less noticeable than pancreatitis caused by other drugs, so it is more likely to develop into pancreatic cancer, leading to more serious consequences.

Quetiapine (823 days), olanzapine (514 days), drospirenone (488 days), valproic acid (437 days), and simvastatin (434 days) are classified as ultra-long latency drugs (median >1 year). Annual pancreatic function assessment (imaging plus enzymology) should be performed in patients with long-term use (>1 year), taking into account their cumulative risk profile (Gukovskaya et al., 2024). In patients with valproic acid use and unexplained abdominal pain, the differential diagnosis should be prioritized to rule out pancreatitis (because of its high fatality rate of 13.41%). Pegaspargase (23 days), and nilotinib (13.5 days), are categorized as short-latency drugs (median <3 months). Intensive monitoring can be implemented during the first month of treatment, including weekly enzyme testing during induction therapy. Prophylactic pancreatic enzyme supplementation can be considered for high-risk regimens (O’brien and Omer, 2019).

The pathway of pancreatitis has not yet been established. We assumed there were one or more pathways, and drugs acting on one or more targets in these pathways could promote or inhibit the occurrence of pancreatitis. We identified 70 potential targets of 50 drugs that induce pancreatitis. In addition, the 70 targets obtained were analyzed and SRC, EGFR and TP53 are considered core targets. Activation of SRC mediate cofilin activation (Ramos-Alvarez et al., 2023), which is important in the regulation of insulin secretin and pancreatic acinar depolymerization/reorganization (Xu et al., 2021) related with pathogenesis of pancreatitis. SRC inhibitors (such as ponatinib) may disrupt barrier function in pancreatic ductal epithelial cells by mediating a actin filaments (Yang et al., 2022). In the pancreas, SRC activation can promote repair after acinar cell injury, but excessive activation can promote necrosis, as well as chemokine/cytokine release to induce inflammation, leading to pathological inflammation (Nuche-Berenguer et al., 2016). EGFR signaling pathway plays an important role in the physiological processes of cell growth, proliferation and differentiation. Studies have shown that there is high or abnormal expression of EGFR in many solid tumors, including pancreatic cancer (Verma et al., 2019). In addition to pancreatic cancer, EGRR is also closely related to the occurrence of pancreatitis. Some studies suggest that hyperactivation of EGFR signaling is related with the induction of pancreatitis (Engle et al., 2019; Fan et al., 2020). Some studies have not found that EGFR is overexpressed in pancreatitis tissues (Chen et al., 2015). EGFR signaling activation plays an important role in inflammation-driven metaplasia and cancer initiation. Tp53 is a tumor suppressor protein encoded by tumor suppressor genes. However, Tp53 expression also promote the development of pancreatitis by acinar apoptosis and injury (Zhou et al., 2019; Tan et al., 2020). The expression of TP53 promotes the release of inflammatory factors, mediates acinar cell apoptosis and inflammatory responses, thereby exacerbating pancreatitis symptoms (Tan et al., 2020). In chronic pancreatitis, the endoplasmic reticulum stress pathway is activated. Studies have shown that endoplasmic reticulum stress markers (ATF6, XBP1, CHOP) are significantly upregulated in both chronic pancreatitis patients and mouse models, accompanied by increased TP53 expression (Zhou et al., 2019). In pancreatic tissues of chronic pancreatitis model mice, acinar cell apoptosis is markedly elevated and correlated with higher TP53 levels. Inhibition of TP53 reduces apoptosis, ameliorates pancreatic structural damage and fibrosis, and lowers the levels of inflammatory factors (IL-6, IL-1β, TNF-α). Therefore, TP53 is a double-edged sword, on the one hand has a protective effect against pancreatic cancer, on the other hand may cause pancreatitis.

Our study has several limitations. First, imperfections in information, such as incorrect inputs and incomplete reports, may lead to bias in the analysis. Second, it was not determined whether the drug had a positive or negative effect on the target; therefore, the results might have been overestimated. Third, although 101 drugs were statistically correlated with pancreatitis, this did not indicate that they were biologically related. Limited by the inherent structure of the FAERS database, the effect of multiple potential confounders could not be adjusted. For instance: a. Pre-existing conditions: Some drugs (e.g., GLP-1 analogs, DPP-4 inhibitors) are mainly used by diabetic patients, and diabetes is an independent risk factor for pancreatitis (Tenner et al., 2024). Even though these drugs show a pancreatitis risk when used for weight loss (Sodhi et al., 2023), the confounding effects of metabolic factors like hyperglycemia, obesity, or insulin resistance cannot be entirely ruled out. b. Interactions between comorbidities and polypharmacy: FAERS lacks complete drug use and clinical data (e.g., lipid levels, history of gallbladder disease), making it impossible to assess the impact of known high-risk conditions for pancreatitis, such as hypertriglyceridemia, cholelithiasis, and autoimmune diseases, which may jointly influence drug use and pancreatitis development (Tenner et al., 2024). c. Drug interactions: For example, diuretics (like hydrochlorothiazide) are often combined with angiotension converting enzyme inhibitors (like lisinopril) for hypertension treatment. Both are identified as signal drugs, but their individual effects and synergistic toxicity cannot be distinguished.

## 5 Conclusion

In summary, disproportionality analysis helped to identify 101 from 867 drugs with pancreatitis report number ≥3 in 64,866 reports, and network pharmacology analysis investigated the toxicological mechanisms of 50. The results of network pharmacology analysis help us to understand the mechanism of drug-induced pancreatitis. Although further investigations are warranted to establish the causality, clinicians providing these therapies should stay vigilant to detect pancreatitis early and consider drug factors to provide targeted interventions when diagnosing and treating patients with pancreatitis. In addition, baseline/follow-up pancreatic assessments are recommended for high-risk drug classes (e.g., GLP-1 analogues, antipsychotics).

## Data Availability

The original contributions presented in the study are included in the article/Supplementary Material, further inquiries can be directed to the corresponding author.

## Abbreviations

FAERS, Food and Drug Administration Adverse Event Reporting System; PT, preferred term; IQR, interquartile range; FDA, the U.S. Food and Drug Administration; ATC, Anatomical Therapeutic Chemical; PPI, protein-protein interactions; GO, gene ontology; KEGG, Kyoto Encyclopedia of Genes and Genomes; BP, biological process; CC, cellular component; MF, molecular function; CI, confidence interval; GLP-1, glucagon-like peptide-1; DPP-4, dipeptidyl peptidase 4; IBS, irritable bowel syndrome.
