# Supplementary material for: Drug-induced pancreatitis: a real-world analysis of the FDA Adverse Event Reporting System and network pharmacology
Source: Front Pharmacol. 2025 Apr 16;16:1564127. doi: 10.3389/fphar.2025.1564127 (PMC12040929; doi:10.3389/fphar.2025.1564127)
Supplement: Supplementary file 1 [file DataSheet1.docx]

Supplemental figure 1. The top 20 most appreciably enriched (A) Biological Process (BP), (B) Cellular Component (CC), (C) Molecular Function (MF), (D) Kyoto Encyclopedia of Genes and Genomes (KEGG) pathway of potential target genes of drug-induced pancreatitis by gene count.


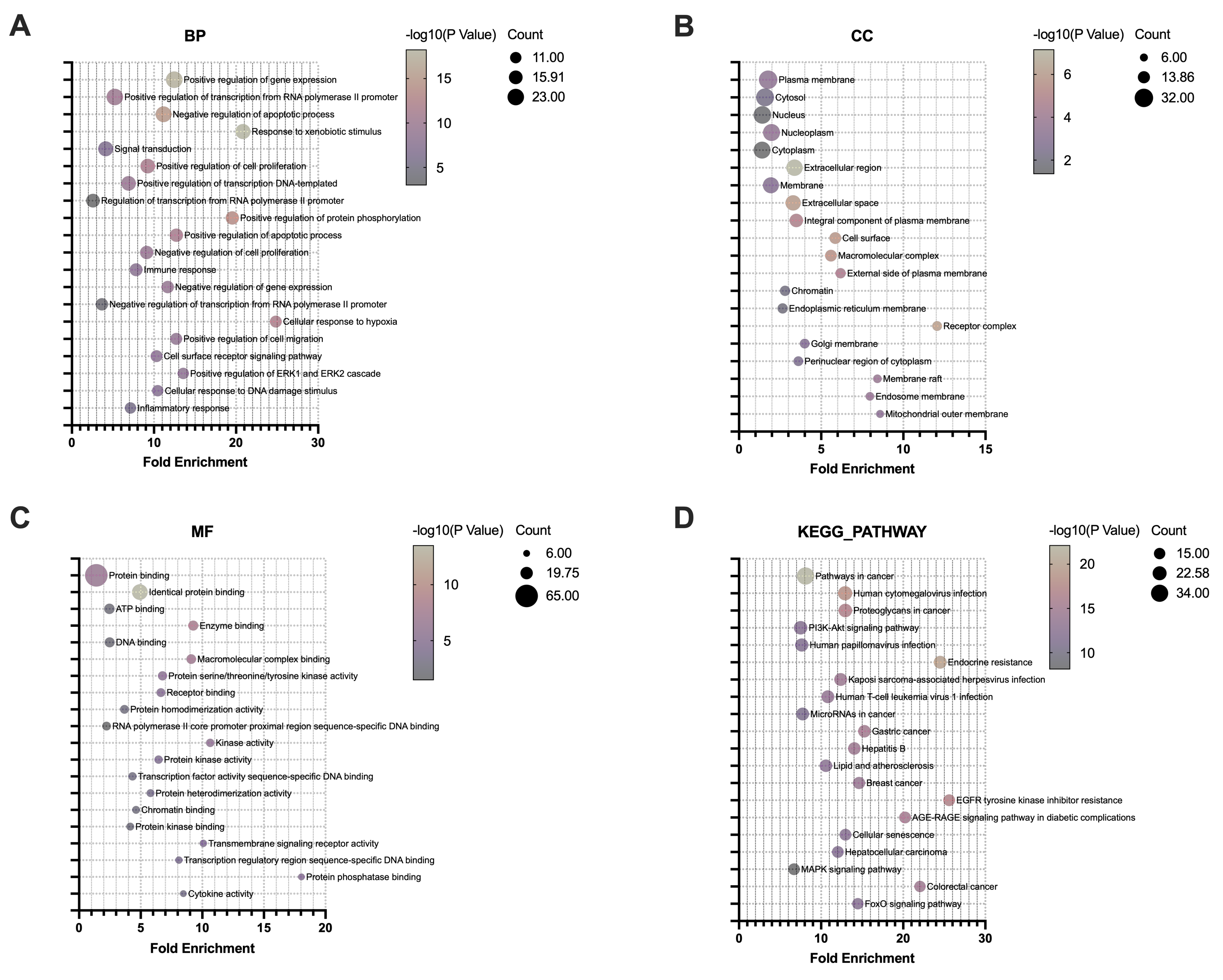


Supplemental table 1. Summary of four algorithms used for signal detection.

| Algorithms | Equation* | Criteria |
| --- | --- | --- |
| ROR | ROR=(a/b)/(c/d) | 95% CI>1, N≥2 |
|  | 95%CI=eln(ROR)±1.96(1/a+1/b+1/c+1/d)^0.5 |  |
| PRR | PRR=(a/(a+c))/(b/(b+d)) | PRR≥2, χ2≥4, N≥3 |
|  | χ2=Σ((O-E)2/E); (O=a，E=(a+b)(a+c)/(a+b+c+d)) |  |
| BCPNN | IC=log2a(a+b+c+d)/((a+c)(a+b)) | IC025>0 |
|  | IC025=eln(IC)-1.96(1/a+1/b+1/c+1/d)^0.5 |  |
| MGPS | EBGM=a(a+b+c+d)/((a+c)(a+b)) | EB05≥2, N>0 |
|  | EB05=eln(EBGM)-1.64(1/a+1/b+1/c+1/d)^0.5 |  |

* a: number of reports containing both the suspect drug and the suspect adverse drug reaction. b: number of reports containing the suspect adverse drug reaction with other medications (except the drug of interest). c: number of reports containing the suspect drug with other adverse drug reactions (except the event of interest). d: number of reports containing other medications and other adverse drug reactions.

Abbreviations: ROR, reporting odds ratio; CI, confidence interval; N, the number of co-occurrences; PRR, proportional reporting ratio; χ2, chi-squared; BCPNN, Bayesian confidence propagation neural network; IC, information component; IC025, the lower limit of the 95% two-sided CI of the IC; MGPS, multi-item gamma Poisson shrinker; EBGM, empirical Bayesian geometric mean; EB05, the lower 90% one-sided CI of EBGM.

Supplemental table 2. The number of acute pancreatitis and chronic pancreatitis.

| Drug | ATC code | Acute pancreatitis | Chronic pancreatitis | Others | Total |
| --- | --- | --- | --- | --- | --- |
| Calcium carbonate | A02AC | 14 | 0 | 17 | 31 |
| Eluxadoline | A07DA | 77 | 2 | 271 | 350 |
| Mesalazine | A07EC | 124 | 2 | 193 | 319 |
| Balsalazide | A07EC | 1 | 0 | 8 | 9 |
| Olsalazine | A07EC | 0 | 0 | 3 | 3 |
| Pancrelipase amylase | A09AA | 74 | 96 | 230 | 400 |
| Metformin | A10BA | 668 | 72 | 960 | 1700 |
| Glimepiride | A10BB | 37 | 5 | 21 | 63 |
| Sitagliptin | A10BH | 571 | 123 | 2473 | 3167 |
| Linagliptin | A10BH | 102 | 8 | 391 | 501 |
| Saxagliptin | A10BH | 78 | 8 | 244 | 330 |
| Alogliptin | A10BH | 61 | 1 | 73 | 135 |
| Exenatide | A10BJ | 799 | 88 | 2323 | 3210 |
| Liraglutide | A10BJ | 633 | 32 | 2200 | 2865 |
| Dulaglutide | A10BJ | 170 | 17 | 1228 | 1415 |
| Semaglutide | A10BJ | 85 | 8 | 557 | 650 |
| Lixisenatide | A10BJ | 10 | 0 | 26 | 36 |
| Albiglutide | A10BJ | 5 | 0 | 2 | 7 |
| Empagliflozin | A10BK | 91 | 1 | 289 | 381 |
| Canagliflozin | A10BK | 84 | 6 | 270 | 360 |
| Dapagliflozin | A10BK | 70 | 2 | 187 | 259 |
| Repaglinide | A10BX | 6 | 0 | 6 | 12 |
| Metreleptin | A16AA | 18 | 3 | 31 | 52 |
| Carglumic acid | A16AA | 0 | 0 | 16 | 16 |
| Levocarnitine | A16AA | 1 | 0 | 3 | 4 |
| Teduglutide | A16AX | 24 | 4 | 73 | 101 |
| Givosiran | A16AX | 3 | 0 | 10 | 13 |
| Hydrochlorothiazide | C03AA | 267 | 35 | 450 | 752 |
| Metolazone | C03BA | 2 | 1 | 8 | 11 |
| Lisinopril | C09AA | 129 | 4 | 173 | 306 |
| Enalapril | C09AA | 45 | 0 | 24 | 69 |
| Perindopril | C09AA | 19 | 2 | 19 | 40 |
| Trandolapril | C09AA | 7 | 0 | 7 | 14 |
| Olmesartan | C09CA | 129 | 29 | 161 | 319 |
| Losartan | C09CA | 87 | 2 | 69 | 158 |
| Candesartan | C09CA | 41 | 0 | 0 | 41 |
| Simvastatin | C10AA | 148 | 17 | 307 | 472 |
| Pravastatin | C10AA | 41 | 1 | 9 | 51 |
| Fenofibrate | C10AB | 74 | 4 | 80 | 158 |
| Fenofibric acid | C10AB | 12 | 1 | 38 | 51 |
| Ezetimibe | C10AX | 107 | 6 | 274 | 387 |
| Bempedoic acid | C10AX | 5 | 0 | 7 | 12 |
| Terbinafine | D01AE | 24 | 2 | 62 | 88 |
| Ethanol | D08AX | 10 | 2 | 45 | 57 |
| Drospirenone | G03AC | 79 | 14 | 496 | 589 |
| Norethisterone | G03AC | 54 | 5 | 167 | 226 |
| Equilin | G03CA | 0 | 0 | 3 | 3 |
| Thiamazole | H03BB | 8 | 0 | 1 | 9 |
| Doxercalciferol | H05BX | 6 | 0 | 3 | 9 |
| Doxycycline | J01AA | 171 | 1 | 113 | 285 |
| Tigecycline | J01AA | 60 | 0 | 119 | 179 |
| Cefpodoxime | J01DD | 9 | 0 | 0 | 9 |
| Metronidazole | J01XD | 109 | 0 | 71 | 180 |
| Tinidazole | J01XD | 4 | 0 | 0 | 4 |
| Linezolid | J01XX | 40 | 1 | 87 | 128 |
| Rifampicin | J04AB | 26 | 0 | 33 | 59 |
| Isoniazid | J04AC | 40 | 0 | 30 | 70 |
| Bedaquiline | J04AK | 5 | 6 | 12 | 23 |
| Pyrazinamide | J04AK | 5 | 0 | 6 | 11 |
| Foscarnet | J05AD | 3 | 0 | 8 | 11 |
| Atazanavir | J05AE | 17 | 0 | 40 | 57 |
| Fosamprenavir | J05AE | 4 | 0 | 6 | 10 |
| Indinavir | J05AE | 4 | 0 | 4 | 8 |
| Abacavir | J05AF | 30 | 6 | 65 | 101 |
| Didanosine | J05AF | 6 | 0 | 45 | 51 |
| Stavudine | J05AF | 12 | 0 | 39 | 51 |
| Nevirapine | J05AG | 22 | 0 | 44 | 66 |
| Raltegravir | J05AJ | 24 | 0 | 41 | 65 |
| Enfuvirtide | J05AX | 9 | 1 | 17 | 27 |
| Mercaptopurine | L01BB | 42 | 2 | 84 | 128 |
| Clofarabine | L01BB | 6 | 0 | 35 | 41 |
| Tioguanine | L01BB | 1 | 0 | 5 | 6 |
| Vincristine | L01CA | 31 | 0 | 40 | 71 |
| Daunorubicin | L01DB | 3 | 0 | 24 | 27 |
| Lenvatinib | L01EX | 20 | 0 | 50 | 70 |
| Brentuximab vedotin | L01FX | 25 | 0 | 64 | 89 |
| Nilotinib | L01XE | 73 | 3 | 372 | 448 |
| Ponatinib | L01XE | 23 | 2 | 157 | 182 |
| Pegaspargase | L01XX | 78 | 1 | 188 | 267 |
| Asparaginase | L01XX | 68 | 3 | 92 | 163 |
| Arsenic trioxide | L01XX | 5 | 0 | 9 | 14 |
| Basiliximab | L04AC | 10 | 0 | 11 | 21 |
| Azathioprine | L04AX | 100 | 0 | 138 | 238 |
| Ketoprofen | M01AE | 12 | 0 | 5 | 17 |
| Flurbiprofen | M01AE | 3 | 0 | 2 | 5 |
| Propofol | N01AX | 29 | 0 | 63 | 92 |
| Codeine | N02AA | 45 | 1 | 31 | 77 |
| Eslicarbazepine | N03AF | 5 | 0 | 3 | 8 |
| Rufinamide | N03AF | 0 | 0 | 6 | 6 |
| Valproic acid | N03AG | 161 | 9 | 270 | 440 |
| Fluphenazine | N05AB | 4 | 0 | 3 | 7 |
| Quetiapine | N05AH | 485 | 68 | 2906 | 3459 |
| Olanzapine | N05AH | 135 | 43 | 1281 | 1459 |
| Meprobamate | N05BC | 3 | 0 | 0 | 3 |
| Riluzole | N07XX | 34 | 0 | 25 | 59 |
| Pentamidine isethionate | P01CX | 1 | 0 | 5 | 6 |
| Miltefosine | P01CX | 0 | 0 | 3 | 3 |
| Calcium acetate | V03AE | 6 | 0 | 3 | 9 |
| Secretin | V04CK | 4 | 0 | 0 | 4 |
| Iodixanol | V08AB | 5 | 1 | 29 | 35 |
| Iothalamic acid | V09CX | 0 | 0 | 7 | 7 |

Supplemental table 3. Association of 266 partial signaling drugs with pancreatitis events.

| Drugs | Reports (n) | ROR | PRR | IC | EBGM | Drugs | Reports (n) | ROR | PRR | IC | EBGM |
| --- | --- | --- | --- | --- | --- | --- | --- | --- | --- | --- | --- |
|  |  | (95% two-sided CI) | (χ2) | (IC025) | (EBGM05) |  |  | (95% two-sided CI) | (χ2) | (IC025) | (EBGM05) |
| Ethinylestradiol | 1451 | 1.52(1.44,1.60) | 1.52(252.37) | 0.59(0.56) | 1.51(1.44) | Meropenem | 17 | 1.29(0.80,2.07) | 1.29(1.08) | 0.36(0.22) | 1.29(0.86) |
| Infliximab | 825 | 1.31(1.23,1.41) | 1.31(60.69) | 0.39(0.36) | 1.31(1.23) | Indomethacin | 17 | 1.80(1.12,2.90) | 1.79(5.97) | 0.84(0.52) | 1.79(1.20) |
| Calcium chloride | 744 | 1.29(1.20,1.38) | 1.29(46.97) | 0.36(0.33) | 1.28(1.21) | Cilastatin | 17 | 1.60(1.00,2.59) | 1.60(3.84) | 0.68(0.42) | 1.60(1.07) |
| Lactic acid | 744 | 1.31(1.22,1.41) | 1.31(53.43) | 0.38(0.36) | 1.30(1.23) | Imipenem | 17 | 1.59(0.99,2.56) | 1.59(3.71) | 0.67(0.41) | 1.59(1.06) |
| Magnesium chloride | 744 | 1.30(1.20,1.39) | 1.29(49.31) | 0.37(0.34) | 1.29(1.21) | Atovaquone | 17 | 1.64(1.02,2.64) | 1.63(4.18) | 0.71(0.44) | 1.63(1.09) |
| Atorvastatin | 438 | 1.41(1.28,1.55) | 1.41(51.62) | 0.49(0.45) | 1.41(1.30) | Proguanil | 17 | 2.27(1.41,3.65) | 2.25(11.91) | 1.17(0.73) | 2.25(1.51) |
| Nivolumab | 375 | 1.59(1.43,1.76) | 1.58(80.49) | 0.66(0.60) | 1.58(1.45) | Zonisamide | 16 | 1.47(0.90,2.40) | 1.47(2.39) | 0.55(0.34) | 1.47(0.97) |
| Rosuvastatin | 343 | 2.10(1.89,2.33) | 2.09(194.29) | 1.06(0.95) | 2.08(1.90) | Doxazosin | 16 | 1.06(0.65,1.74) | 1.06(0.06) | 0.09(0.05) | 1.06(0.70) |
| Omeprazole | 291 | 1.76(1.57,1.97) | 1.75(94.03) | 0.81(0.72) | 1.75(1.59) | Micafungin | 16 | 2.05(1.25,3.35) | 2.04(8.48) | 1.03(0.63) | 2.04(1.35) |
| Tocilizumab | 284 | 1.46(1.30,1.64) | 1.46(40.82) | 0.54(0.48) | 1.46(1.32) | Pramlintide | 15 | 1.52(0.91,2.52) | 1.51(2.62) | 0.60(0.36) | 1.51(0.99) |
| Aripiprazole | 266 | 1.10(0.98,1.24) | 1.10(2.46) | 0.14(0.12) | 1.10(0.99) | Pasireotide | 15 | 2.39(1.44,3.98) | 2.38(12.04) | 1.25(0.75) | 2.38(1.55) |
| Tacrolimus | 264 | 1.29(1.14,1.46) | 1.29(17.14) | 0.37(0.32) | 1.29(1.16) | Itraconazole | 15 | 1.12(0.68,1.87) | 1.12(0.21) | 0.17(0.10) | 1.12(0.74) |
| Bevacizumab | 263 | 1.09(0.97,1.23) | 1.09(1.99) | 0.12(0.11) | 1.09(0.99) | Sodium phosphate | 15 | 1.13(0.68,1.87) | 1.13(0.21) | 0.17(0.10) | 1.13(0.74) |
| Emtricitabine | 231 | 1.25(1.10,1.42) | 1.25(11.49) | 0.32(0.28) | 1.25(1.12) | Glipizide | 14 | 1.04(0.61,1.75) | 1.04(0.02) | 0.05(0.03) | 1.04(0.67) |
| Pembrolizumab | 229 | 1.67(1.47,1.90) | 1.66(60.74) | 0.73(0.64) | 1.66(1.49) | Acitretin | 14 | 1.83(1.08,3.09) | 1.82(5.19) | 0.86(0.51) | 1.82(1.17) |
| Vedolizumab | 224 | 1.31(1.14,1.49) | 1.30(15.88) | 0.38(0.33) | 1.30(1.17) | Caspofungin | 14 | 1.62(0.96,2.73) | 1.61(3.26) | 0.69(0.41) | 1.61(1.04) |
| Pantoprazole | 205 | 1.90(1.66,2.18) | 1.89(86.35) | 0.92(0.80) | 1.89(1.68) | Follitropin | 14 | 1.21(0.72,2.05) | 1.21(0.52) | 0.28(0.16) | 1.21(0.78) |
| Prednisolone | 196 | 1.16(1.01,1.34) | 1.16(4.39) | 0.22(0.19) | 1.16(1.03) | Lubiprostone | 14 | 2.47(1.46,4.18) | 2.45(12.11) | 1.30(0.76) | 2.45(1.58) |
| Ritonavir | 184 | 1.74(1.50,2.01) | 1.73(57.14) | 0.79(0.68) | 1.73(1.53) | Icatibant | 13 | 1.01(0.59,1.74) | 1.01(0.00) | 0.01(0.01) | 1.01(0.64) |
| Tramadol | 180 | 1.53(1.32,1.78) | 1.53(33.15) | 0.61(0.53) | 1.53(1.35) | Anagrelide | 13 | 2.14(1.24,3.69) | 2.13(7.78) | 1.09(0.63) | 2.13(1.35) |
| Prednisone | 177 | 1.64(1.41,1.90) | 1.63(43.30) | 0.70(0.61) | 1.63(1.44) | Tesamorelin | 13 | 1.30(0.75,2.24) | 1.30(0.89) | 0.38(0.22) | 1.30(0.82) |
| Alendronic acid | 176 | 1.65(1.42,1.91) | 1.64(44.53) | 0.72(0.62) | 1.64(1.45) | Desloratadine | 13 | 1.75(1.01,3.02) | 1.74(4.13) | 0.80(0.46) | 1.74(1.10) |
| Dexamethasone | 172 | 1.21(1.05,1.41) | 1.21(6.45) | 0.28(0.24) | 1.21(1.07) | Ceritinib | 12 | 1.56(0.88,2.75) | 1.55(2.37) | 0.63(0.36) | 1.55(0.97) |
| Lamivudine | 163 | 1.83(1.57,2.14) | 1.83(60.96) | 0.87(0.74) | 1.82(1.60) | Cefazolin | 12 | 1.85(1.05,3.27) | 1.85(4.67) | 0.88(0.50) | 1.85(1.15) |
| Octreotide | 162 | 1.72(1.48,2.01) | 1.72(48.83) | 0.78(0.67) | 1.72(1.51) | Ursodeoxycholic acid | 12 | 1.87(1.06,3.30) | 1.86(4.82) | 0.90(0.51) | 1.86(1.16) |
| Amoxicillin | 154 | 1.23(1.05,1.45) | 1.23(6.80) | 0.30(0.26) | 1.23(1.08) | Sevoflurane | 12 | 1.03(0.58,1.82) | 1.03(0.01) | 0.04(0.02) | 1.03(0.64) |
| Ivacaftor | 153 | 1.55(1.32,1.82) | 1.55(29.84) | 0.63(0.54) | 1.55(1.35) | Fluvastatin | 11 | 2.24(1.23,4.05) | 2.22(7.44) | 1.15(0.64) | 2.22(1.35) |
| Sunitinib | 145 | 1.01(0.86,1.19) | 1.01(0.01) | 0.01(0.01) | 1.01(0.88) | Avelumab | 11 | 1.70(0.94,3.08) | 1.70(3.15) | 0.76(0.42) | 1.70(1.03) |
| Orlistat | 144 | 1.52(1.29,1.79) | 1.51(25.16) | 0.60(0.51) | 1.51(1.32) | Interferon alfa-2a | 11 | 1.75(0.97,3.17) | 1.74(3.51) | 0.80(0.44) | 1.74(1.06) |
| Doxorubicin | 144 | 1.30(1.10,1.53) | 1.29(9.66) | 0.37(0.32) | 1.29(1.13) | Brodalumab | 11 | 2.77(1.53,5.02) | 2.75(12.32) | 1.46(0.81) | 2.75(1.67) |
| Ramipril | 138 | 2.16(1.82,2.55) | 2.15(84.66) | 1.10(0.93) | 2.14(1.86) | Tisagenlecleucel | 11 | 1.05(0.58,1.90) | 1.05(0.03) | 0.07(0.04) | 1.05(0.64) |
| Sorafenib | 130 | 1.75(1.48,2.08) | 1.75(41.73) | 0.80(0.68) | 1.75(1.51) | Adefovir dipivoxil | 11 | 1.50(0.83,2.71) | 1.49(1.80) | 0.58(0.32) | 1.49(0.91) |
| Furosemide | 130 | 1.64(1.38,1.95) | 1.64(32.29) | 0.71(0.60) | 1.64(1.42) | Cefotaxime | 11 | 2.84(1.57,5.14) | 2.82(12.93) | 1.49(0.82) | 2.82(1.71) |
| Ipilimumab | 127 | 2.00(1.68,2.38) | 1.99(62.58) | 0.99(0.83) | 1.99(1.72) | Etravirine | 11 | 3.12(1.72,5.65) | 3.09(15.63) | 1.63(0.90) | 3.09(1.88) |
| Paracetamol | 121 | 1.90(1.59,2.27) | 1.89(51.19) | 0.92(0.77) | 1.89(1.63) | Eplerenone | 11 | 1.74(0.96,3.15) | 1.73(3.43) | 0.79(0.44) | 1.73(1.06) |
| Cabozantinib | 119 | 1.10(0.92,1.32) | 1.10(1.14) | 0.14(0.12) | 1.10(0.95) | Brigatinib | 11 | 1.33(0.74,2.41) | 1.33(0.91) | 0.41(0.23) | 1.33(0.81) |
| Atezolizumab | 118 | 1.78(1.49,2.14) | 1.78(40.08) | 0.83(0.69) | 1.77(1.52) | Cobimetinib | 11 | 1.79(0.99,3.25) | 1.79(3.84) | 0.84(0.46) | 1.79(1.09) |
| Ribavirin | 109 | 1.67(1.38,2.01) | 1.66(28.84) | 0.73(0.61) | 1.66(1.42) | Neratinib | 11 | 1.78(0.98,3.21) | 1.77(3.71) | 0.82(0.46) | 1.77(1.08) |
| Pazopanib | 104 | 1.10(0.90,1.33) | 1.10(0.86) | 0.13(0.11) | 1.09(0.93) | Vildagliptin | 10 | 1.39(0.75,2.59) | 1.39(1.10) | 0.48(0.26) | 1.39(0.83) |
| Deferasirox | 104 | 1.21(1.00,1.47) | 1.21(3.77) | 0.27(0.23) | 1.21(1.03) | Pitavastatin | 10 | 1.61(0.86,3.00) | 1.61(2.29) | 0.68(0.37) | 1.61(0.95) |
| Methylprednisolone | 101 | 1.12(0.92,1.36) | 1.12(1.23) | 0.16(0.13) | 1.12(0.95) | Boceprevir | 10 | 2.28(1.23,4.26) | 2.27(7.15) | 1.18(0.63) | 2.27(1.35) |
| Gemcitabine | 97 | 1.04(0.85,1.26) | 1.03(0.11) | 0.05(0.04) | 1.03(0.88) | Perampanel | 10 | 1.23(0.66,2.29) | 1.23(0.44) | 0.30(0.16) | 1.23(0.73) |
| Tenofovir alafenamide | 95 | 1.92(1.57,2.35) | 1.91(41.33) | 0.93(0.76) | 1.91(1.61) | Glibenclamide | 10 | 2.02(1.09,3.77) | 2.01(5.12) | 1.01(0.54) | 2.01(1.20) |
| Desogestrel | 95 | 2.11(1.72,2.58) | 2.10(54.69) | 1.07(0.87) | 2.10(1.77) | Calcitriol | 10 | 2.19(1.18,4.08) | 2.18(6.42) | 1.12(0.60) | 2.18(1.30) |
| Trimethoprim | 92 | 2.00(1.63,2.46) | 2.00(45.81) | 1.00(0.81) | 1.99(1.68) | Lovastatin | 9 | 1.90(0.98,3.66) | 1.89(3.79) | 0.92(0.48) | 1.89(1.09) |
| Mirtazapine | 89 | 1.40(1.13,1.72) | 1.40(9.99) | 0.48(0.39) | 1.39(1.17) | Cimetidine | 9 | 2.43(1.26,4.68) | 2.41(7.48) | 1.27(0.66) | 2.41(1.39) |
| Ceftriaxone | 88 | 2.34(1.90,2.89) | 2.33(66.72) | 1.22(0.99) | 2.32(1.95) | Clomipramine | 9 | 1.94(1.00,3.73) | 1.93(4.04) | 0.95(0.49) | 1.93(1.11) |
| Sulfamethoxazole | 82 | 2.20(1.77,2.74) | 2.19(53.23) | 1.13(0.91) | 2.19(1.83) | Ixabepilone | 9 | 1.62(0.84,3.13) | 1.62(2.14) | 0.70(0.36) | 1.62(0.94) |
| Loperamide | 80 | 1.25(1.01,1.56) | 1.25(4.08) | 0.32(0.26) | 1.25(1.04) | Fosfomycin | 8 | 2.83(1.41,5.67) | 2.80(9.33) | 1.49(0.74) | 2.80(1.57) |
| Memantine | 79 | 2.20(1.76,2.74) | 2.19(51.07) | 1.13(0.90) | 2.19(1.82) | Soybean oil | 8 | 1.92(0.96,3.85) | 1.91(3.51) | 0.94(0.47) | 1.91(1.07) |
| Dolutegravir | 78 | 1.91(1.53,2.38) | 1.90(33.37) | 0.93(0.74) | 1.90(1.58) | Isatuximab | 8 | 1.72(0.86,3.44) | 1.71(2.38) | 0.78(0.39) | 1.71(0.96) |
| Carisoprodol | 77 | 1.23(0.99,1.54) | 1.23(3.39) | 0.30(0.24) | 1.23(1.02) | Daclatasvir | 8 | 1.66(0.83,3.34) | 1.66(2.11) | 0.73(0.36) | 1.66(0.93) |
| Leflunomide | 75 | 1.22(0.97,1.53) | 1.22(2.98) | 0.29(0.23) | 1.22(1.01) | Dapsone | 8 | 1.55(0.78,3.12) | 1.55(1.57) | 0.63(0.32) | 1.55(0.87) |
| Pioglitazone | 75 | 1.15(0.92,1.44) | 1.15(1.47) | 0.20(0.16) | 1.15(0.95) | Inotuzumab ozogamicin | 8 | 2.43(1.21,4.88) | 2.42(6.67) | 1.27(0.63) | 2.42(1.35) |
| Nintedanib | 73 | 1.16(0.93,1.47) | 1.16(1.69) | 0.22(0.17) | 1.16(0.96) | Ethiodized oil | 8 | 2.49(1.24,4.99) | 2.47(7.05) | 1.31(0.65) | 2.47(1.38) |
| Vemurafenib | 72 | 1.96(1.55,2.47) | 1.95(33.37) | 0.96(0.76) | 1.95(1.60) | Butalbital | 8 | 1.54(0.77,3.08) | 1.53(1.49) | 0.62(0.31) | 1.53(0.86) |
| Axitinib | 71 | 1.36(1.08,1.72) | 1.36(6.85) | 0.45(0.35) | 1.36(1.12) | Ertugliflozin | 7 | 2.66(1.26,5.61) | 2.65(7.20) | 1.40(0.67) | 2.65(1.42) |
| Cytarabine | 69 | 1.60(1.26,2.03) | 1.60(15.41) | 0.67(0.53) | 1.60(1.31) | Tipranavir | 7 | 2.90(1.38,6.12) | 2.88(8.63) | 1.53(0.72) | 2.88(1.54) |
| Telaprevir | 67 | 1.96(1.54,2.50) | 1.96(31.46) | 0.97(0.76) | 1.96(1.60) | Vandetanib | 7 | 1.51(0.72,3.17) | 1.51(1.20) | 0.59(0.28) | 1.51(0.81) |
| Irbesartan | 65 | 1.88(1.47,2.40) | 1.87(26.50) | 0.90(0.71) | 1.87(1.53) | Mitoxantrone | 7 | 2.10(1.00,4.41) | 2.09(3.97) | 1.06(0.50) | 2.09(1.12) |
| Efavirenz | 64 | 1.10(0.86,1.41) | 1.10(0.62) | 0.14(0.11) | 1.10(0.90) | Maraviroc | 7 | 1.71(0.81,3.59) | 1.70(2.03) | 0.77(0.36) | 1.70(0.91) |
| Trametinib | 62 | 1.92(1.49,2.46) | 1.91(26.88) | 0.93(0.73) | 1.91(1.55) | Galantamine | 7 | 1.48(0.71,3.12) | 1.48(1.10) | 0.57(0.27) | 1.48(0.80) |
| Allopurinol | 61 | 1.80(1.40,2.32) | 1.80(21.63) | 0.85(0.66) | 1.80(1.45) | Cefalexin | 7 | 1.38(0.66,2.90) | 1.38(0.72) | 0.46(0.22) | 1.38(0.74) |
| Aciclovir | 61 | 2.40(1.86,3.09) | 2.39(49.25) | 1.25(0.97) | 2.38(1.93) | Calcium folinate | 7 | 2.28(1.08,4.79) | 2.26(4.96) | 1.18(0.56) | 2.26(1.21) |
| Irinotecan | 60 | 1.32(1.03,1.70) | 1.32(4.66) | 0.40(0.31) | 1.32(1.07) | Ecallantide | 7 | 1.51(0.72,3.18) | 1.51(1.21) | 0.60(0.28) | 1.51(0.81) |
| Fluconazole | 60 | 1.78(1.38,2.29) | 1.77(20.20) | 0.82(0.64) | 1.77(1.43) | Lanadelumab | 7 | 1.02(0.49,2.15) | 1.02(0.00) | 0.03(0.01) | 1.02(0.55) |
| Epoprostenol | 59 | 1.26(0.98,1.63) | 1.26(3.23) | 0.34(0.26) | 1.26(1.02) | Theophylline | 7 | 1.34(0.64,2.83) | 1.34(0.61) | 0.42(0.20) | 1.34(0.72) |
| Tezacaftor | 59 | 1.14(0.88,1.47) | 1.13(0.94) | 0.18(0.14) | 1.13(0.92) | Triamterene | 7 | 1.95(0.93,4.10) | 1.94(3.22) | 0.96(0.46) | 1.94(1.04) |
| Dabrafenib | 58 | 1.28(0.99,1.65) | 1.28(3.48) | 0.35(0.27) | 1.28(1.03) | Bismuth subcitrate potassium | 7 | 3.05(1.45,6.43) | 3.03(9.54) | 1.60(0.76) | 3.03(1.62) |
| Lopinavir | 58 | 2.37(1.83,3.07) | 2.36(45.41) | 1.24(0.95) | 2.35(1.90) | Nelfinavir | 6 | 2.46(1.10,5.50) | 2.45(5.16) | 1.29(0.58) | 2.45(1.25) |
| Clarithromycin | 57 | 1.02(0.79,1.32) | 1.02(0.02) | 0.03(0.02) | 1.02(0.82) | Famciclovir | 6 | 2.50(1.12,5.60) | 2.49(5.37) | 1.32(0.59) | 2.49(1.27) |
| Insulin degludec | 56 | 1.75(1.35,2.28) | 1.75(17.89) | 0.80(0.62) | 1.74(1.40) | Ceftazidime | 6 | 1.07(0.48,2.38) | 1.07(0.03) | 0.09(0.04) | 1.07(0.55) |
| Regorafenib | 54 | 1.60(1.22,2.09) | 1.59(11.96) | 0.67(0.51) | 1.59(1.27) | Glycerol phenylbutyrate | 6 | 1.51(0.67,3.36) | 1.50(1.01) | 0.59(0.26) | 1.50(0.77) |
| Clavulanic acid | 54 | 1.37(1.05,1.79) | 1.37(5.43) | 0.45(0.35) | 1.37(1.10) | Betaine | 6 | 2.03(0.91,4.54) | 2.02(3.12) | 1.02(0.46) | 2.02(1.03) |
| Lanreotide | 53 | 1.95(1.49,2.56) | 1.95(24.46) | 0.96(0.73) | 1.95(1.55) | Cholestyramine | 6 | 1.13(0.50,2.51) | 1.12(0.08) | 0.17(0.08) | 1.12(0.57) |
| Insulin detemir | 52 | 1.31(1.00,1.72) | 1.31(3.83) | 0.39(0.30) | 1.31(1.04) | Silodosin | 6 | 1.01(0.45,2.25) | 1.01(0.00) | 0.01(0.01) | 1.01(0.52) |
| Remdesivir | 51 | 1.72(1.31,2.27) | 1.72(15.29) | 0.78(0.59) | 1.72(1.36) | Simeprevir | 6 | 2.28(1.02,5.09) | 2.26(4.25) | 1.18(0.53) | 2.26(1.16) |
| Elexacaftor | 51 | 1.57(1.19,2.07) | 1.57(10.45) | 0.65(0.49) | 1.56(1.24) | Bisacodyl | 6 | 1.16(0.52,2.59) | 1.16(0.14) | 0.22(0.10) | 1.16(0.59) |
| Peginterferon alfa-2b | 50 | 1.77(1.34,2.34) | 1.77(16.68) | 0.82(0.62) | 1.77(1.40) | Vorinostat | 6 | 1.07(0.48,2.38) | 1.07(0.03) | 0.09(0.04) | 1.07(0.55) |
| Mycophenolic acid | 49 | 1.32(1.00,1.75) | 1.32(3.76) | 0.40(0.30) | 1.32(1.04) | Sulbactam | 6 | 1.06(0.48,2.36) | 1.06(0.02) | 0.08(0.04) | 1.06(0.54) |
| Ondansetron | 49 | 1.14(0.86,1.50) | 1.14(0.79) | 0.18(0.14) | 1.14(0.90) | Captopril | 5 | 2.44(1.01,5.90) | 2.43(4.23) | 1.28(0.53) | 2.43(1.16) |
| Atenolol | 48 | 1.27(0.95,1.68) | 1.27(2.69) | 0.34(0.26) | 1.27(1.00) | Mitotane | 5 | 1.87(0.78,4.52) | 1.87(2.02) | 0.90(0.37) | 1.87(0.89) |
| Icodextrin | 48 | 1.77(1.34,2.36) | 1.77(16.11) | 0.82(0.62) | 1.77(1.39) | Dacarbazine | 5 | 1.79(0.74,4.32) | 1.79(1.74) | 0.84(0.35) | 1.79(0.86) |
| Bosutinib | 48 | 2.14(1.61,2.84) | 2.13(28.77) | 1.09(0.82) | 2.13(1.68) | Dronabinol | 5 | 1.88(0.78,4.52) | 1.87(2.03) | 0.90(0.37) | 1.87(0.90) |
| Alemtuzumab | 46 | 1.04(0.78,1.39) | 1.04(0.07) | 0.05(0.04) | 1.04(0.81) | Anidulafungin | 5 | 3.26(1.35,7.87) | 3.23(7.71) | 1.69(0.70) | 3.23(1.54) |
| Zidovudine | 46 | 1.76(1.32,2.36) | 1.76(15.13) | 0.81(0.61) | 1.76(1.38) | Chlortalidone | 5 | 1.26(0.52,3.03) | 1.26(0.26) | 0.33(0.14) | 1.26(0.60) |
| Bisoprolol | 46 | 1.13(0.84,1.51) | 1.13(0.66) | 0.17(0.13) | 1.13(0.88) | Trabectedin | 5 | 1.03(0.43,2.48) | 1.03(0.00) | 0.04(0.02) | 1.03(0.49) |
| Bictegravir | 45 | 2.38(1.77,3.19) | 2.36(35.46) | 1.24(0.92) | 2.36(1.85) | Cyanocobalamin | 5 | 1.92(0.80,4.63) | 1.91(2.19) | 0.94(0.39) | 1.91(0.92) |
| Cobicistat | 43 | 1.71(1.26,2.30) | 1.70(12.45) | 0.77(0.57) | 1.70(1.32) | Aluminum hydroxide | 5 | 2.42(1.00,5.83) | 2.40(4.11) | 1.26(0.52) | 2.40(1.15) |
| Durvalumab | 42 | 1.70(1.26,2.31) | 1.70(12.07) | 0.76(0.56) | 1.70(1.32) | Bazedoxifene | 5 | 1.39(0.58,3.34) | 1.38(0.54) | 0.47(0.19) | 1.38(0.66) |
| Interferon alfa-2b | 39 | 2.48(1.81,3.40) | 2.46(34.00) | 1.30(0.95) | 2.46(1.89) | Saquinavir | 4 | 2.47(0.92,6.62) | 2.46(3.47) | 1.30(0.48) | 2.46(1.08) |
| Telmisartan | 39 | 1.38(1.01,1.89) | 1.38(4.07) | 0.46(0.34) | 1.38(1.06) | Nelarabine | 4 | 2.01(0.75,5.37) | 2.00(2.01) | 1.00(0.37) | 2.00(0.88) |
| Elvitegravir | 39 | 1.82(1.33,2.50) | 1.81(14.31) | 0.86(0.63) | 1.81(1.39) | Torasemide | 4 | 1.35(0.51,3.62) | 1.35(0.37) | 0.44(0.16) | 1.35(0.59) |
| Amphotericin b | 38 | 1.66(1.20,2.28) | 1.65(9.79) | 0.72(0.53) | 1.65(1.26) | Deferoxamine | 4 | 1.05(0.39,2.79) | 1.05(0.01) | 0.06(0.02) | 1.05(0.46) |
| Darunavir | 36 | 2.12(1.53,2.94) | 2.11(21.14) | 1.08(0.78) | 2.11(1.60) | Cefixime | 4 | 2.79(1.04,7.47) | 2.77(4.54) | 1.47(0.55) | 2.77(1.21) |
| Sirolimus | 35 | 1.00(0.72,1.39) | 1.00(0.00) | 0.00(0.00) | 1.00(0.76) | Nitisinone | 4 | 1.17(0.44,3.13) | 1.17(0.10) | 0.23(0.09) | 1.17(0.51) |
| Sulfasalazine | 34 | 1.21(0.86,1.69) | 1.21(1.22) | 0.27(0.19) | 1.21(0.91) | Danaparoid | 4 | 2.34(0.88,6.27) | 2.33(3.05) | 1.22(0.46) | 2.33(1.02) |
| Melphalan | 34 | 1.43(1.02,2.01) | 1.43(4.44) | 0.52(0.37) | 1.43(1.08) | Alosetron | 4 | 1.57(0.59,4.19) | 1.56(0.81) | 0.64(0.24) | 1.56(0.69) |
| Tamoxifen | 33 | 2.08(1.47,2.93) | 2.07(18.26) | 1.05(0.74) | 2.07(1.55) | Sargramostim | 4 | 1.21(0.45,3.23) | 1.21(0.15) | 0.27(0.10) | 1.21(0.53) |
| Rabeprazole | 32 | 2.25(1.59,3.19) | 2.24(22.12) | 1.17(0.82) | 2.24(1.68) | Clomifene | 4 | 3.12(1.16,8.36) | 3.09(5.68) | 1.63(0.61) | 3.09(1.35) |
| Epirubicin | 32 | 1.49(1.05,2.11) | 1.49(5.10) | 0.57(0.40) | 1.49(1.11) | Caplacizumab | 4 | 1.17(0.44,3.13) | 1.17(0.10) | 0.23(0.09) | 1.17(0.52) |
| Entecavir | 32 | 1.68(1.19,2.38) | 1.68(8.83) | 0.75(0.53) | 1.68(1.26) | Etodolac | 4 | 1.75(0.65,4.67) | 1.74(1.27) | 0.80(0.30) | 1.74(0.76) |
| Minocycline | 31 | 1.59(1.12,2.27) | 1.59(6.77) | 0.67(0.47) | 1.59(1.18) | Atracurium | 4 | 1.23(0.46,3.28) | 1.23(0.17) | 0.30(0.11) | 1.23(0.54) |
| Dronedarone | 31 | 1.45(1.02,2.07) | 1.45(4.34) | 0.54(0.38) | 1.45(1.08) | Porfimer sodium | 4 | 3.17(1.18,8.49) | 3.14(5.86) | 1.65(0.62) | 3.14(1.38) |
| Erythromycin | 31 | 1.23(0.86,1.74) | 1.22(1.28) | 0.29(0.21) | 1.22(0.91) | Eprosartan | 3 | 2.56(0.82,7.97) | 2.54(2.81) | 1.35(0.43) | 2.54(0.98) |
| Aliskiren | 31 | 1.23(0.87,1.76) | 1.23(1.37) | 0.30(0.21) | 1.23(0.92) | Efalizumab | 3 | 5.08(1.62,15.93) | 5.00(9.63) | 2.32(0.74) | 5.00(1.92) |
| Corticotropin | 31 | 1.16(0.81,1.65) | 1.16(0.66) | 0.21(0.15) | 1.16(0.86) | Entrectinib | 3 | 1.07(0.34,3.31) | 1.07(0.01) | 0.09(0.03) | 1.07(0.41) |
| Blinatumomab | 28 | 1.23(0.85,1.79) | 1.23(1.24) | 0.30(0.21) | 1.23(0.90) | Nicardipine | 3 | 1.14(0.37,3.55) | 1.14(0.05) | 0.19(0.06) | 1.14(0.44) |
| Cefuroxime | 26 | 1.41(0.96,2.08) | 1.41(3.12) | 0.50(0.34) | 1.41(1.02) | Acamprosate | 3 | 1.40(0.45,4.35) | 1.40(0.34) | 0.48(0.15) | 1.39(0.54) |
| Posaconazole | 25 | 2.18(1.47,3.24) | 2.17(15.89) | 1.12(0.75) | 2.17(1.56) | Colesevelam | 3 | 3.07(0.98,9.58) | 3.04(4.13) | 1.61(0.51) | 3.04(1.17) |
| Afatinib | 25 | 1.15(0.78,1.71) | 1.15(0.49) | 0.20(0.14) | 1.15(0.83) | Alpelisib | 3 | 1.16(0.37,3.60) | 1.16(0.06) | 0.21(0.07) | 1.16(0.45) |
| Febuxostat | 24 | 1.41(0.94,2.10) | 1.41(2.83) | 0.49(0.33) | 1.41(1.00) | Rasburicase | 3 | 1.58(0.51,4.93) | 1.58(0.64) | 0.66(0.21) | 1.58(0.61) |
| Omega-3-acid ethyl esters | 24 | 1.45(0.97,2.17) | 1.45(3.37) | 0.54(0.36) | 1.45(1.04) | Pentoxifylline | 3 | 2.64(0.85,8.23) | 2.62(3.02) | 1.39(0.45) | 2.62(1.01) |
| Colchicine | 24 | 2.17(1.45,3.24) | 2.16(14.93) | 1.11(0.74) | 2.16(1.54) | Aldesleukin | 3 | 1.34(0.43,4.17) | 1.34(0.26) | 0.42(0.14) | 1.34(0.52) |
| Idelalisib | 24 | 1.05(0.70,1.57) | 1.05(0.05) | 0.07(0.05) | 1.05(0.75) | Acetylcysteine | 3 | 1.18(0.38,3.68) | 1.18(0.09) | 0.24(0.08) | 1.18(0.46) |
| Aprepitant | 24 | 1.53(1.02,2.28) | 1.52(4.34) | 0.61(0.41) | 1.52(1.09) | Clofazimine | 3 | 1.38(0.44,4.30) | 1.38(0.31) | 0.46(0.15) | 1.38(0.53) |
| Indapamide | 24 | 2.60(1.74,3.88) | 2.58(23.31) | 1.37(0.91) | 2.58(1.84) | Methyldopa | 3 | 1.73(0.56,5.38) | 1.72(0.91) | 0.79(0.25) | 1.72(0.67) |
| Encorafenib | 22 | 1.87(1.23,2.84) | 1.86(8.81) | 0.90(0.59) | 1.86(1.31) | Fluorescein | 3 | 1.18(0.38,3.68) | 1.18(0.09) | 0.24(0.08) | 1.18(0.46) |
| Lanthanum carbonate | 22 | 2.33(1.53,3.55) | 2.32(16.54) | 1.21(0.80) | 2.32(1.63) | Nateglinide | 2 | 4.34(1.07,17.58) | 4.29(5.06) | 2.10(0.52) | 4.29(1.33) |
| Benazepril | 21 | 2.09(1.36,3.21) | 2.08(11.81) | 1.06(0.69) | 2.08(1.45) | Interferon alfacon-1 | 2 | 1.64(0.41,6.60) | 1.64(0.50) | 0.71(0.18) | 1.64(0.51) |
| Risedronic acid | 21 | 1.25(0.81,1.91) | 1.25(1.02) | 0.32(0.21) | 1.24(0.87) | Daclizumab | 2 | 1.05(0.26,4.22) | 1.05(0.00) | 0.07(0.02) | 1.05(0.33) |
| Hydroxycarbamide | 20 | 2.57(1.66,4.00) | 2.56(19.06) | 1.35(0.87) | 2.56(1.77) | Fosinopril | 2 | 1.14(0.29,4.59) | 1.14(0.04) | 0.19(0.05) | 1.14(0.36) |
| Roflumilast | 20 | 1.84(1.19,2.86) | 1.83(7.62) | 0.88(0.56) | 1.83(1.27) | Bendroflumethiazide | 2 | 6.06(1.49,24.65) | 5.94(8.25) | 2.57(0.63) | 5.94(1.84) |
| Telithromycin | 19 | 1.79(1.14,2.81) | 1.78(6.55) | 0.83(0.53) | 1.78(1.22) | Telbivudine | 2 | 3.96(0.98,16.01) | 3.91(4.35) | 1.97(0.49) | 3.91(1.22) |
| Alectinib | 19 | 1.03(0.66,1.62) | 1.03(0.02) | 0.05(0.03) | 1.03(0.71) | Dicycloverine | 2 | 2.63(0.65,10.57) | 2.61(1.99) | 1.38(0.34) | 2.61(0.81) |
| Nitrofurantoin | 19 | 1.16(0.74,1.82) | 1.16(0.42) | 0.21(0.14) | 1.16(0.80) | Sodium phosphate, monobasic | 2 | 1.67(0.42,6.71) | 1.67(0.53) | 0.74(0.18) | 1.67(0.52) |
| Baricitinib | 18 | 1.04(0.65,1.65) | 1.04(0.03) | 0.06(0.04) | 1.04(0.71) | Levomefolic acid | 2 | 8.46(2.07,34.66) | 8.22(12.73) | 3.04(0.74) | 8.21(2.53) |
| Tetracycline | 18 | 2.24(1.41,3.56) | 2.23(12.20) | 1.15(0.73) | 2.23(1.51) | Cefoperazone | 2 | 1.21(0.30,4.85) | 1.21(0.07) | 0.27(0.07) | 1.21(0.38) |
| Ganciclovir | 18 | 1.44(0.91,2.29) | 1.44(2.40) | 0.52(0.33) | 1.44(0.98) | Pamidronic acid | 1 | 2.03(0.28,14.52) | 2.02(0.52) | 1.01(0.14) | 2.02(0.39) |
| Tegaserod | 18 | 1.47(0.92,2.33) | 1.46(2.65) | 0.55(0.35) | 1.46(0.99) | Drotrecogin alfa | 1 | 2.21(0.31,15.84) | 2.20(0.66) | 1.14(0.16) | 2.20(0.42) |
| Binimetinib | 18 | 1.68(1.05,2.66) | 1.67(4.88) | 0.74(0.47) | 1.67(1.13) | Sulindac | 1 | 1.78(0.25,12.72) | 1.77(0.34) | 0.83(0.12) | 1.77(0.34) |
| Gemtuzumab ozogamicin | 17 | 2.12(1.31,3.41) | 2.11(9.91) | 1.07(0.67) | 2.11(1.41) | Nalbuphine | 1 | 2.50(0.35,17.96) | 2.49(0.89) | 1.32(0.18) | 2.49(0.48) |
| Alfuzosin | 17 | 2.17(1.35,3.50) | 2.16(10.64) | 1.11(0.69) | 2.16(1.45) | Diatrizoic acid | 1 | 1.49(0.21,10.62) | 1.48(0.16) | 0.57(0.08) | 1.48(0.29) |

Abbreviations: ROR, reporting odds ratio; CI, confidence interval; PRR, proportional reporting ratio; χ2, chi-squared; IC, information component; IC025, the lower limit of the 95% two-sided CI of the IC; EBGM, empirical Bayesian geometric mean; EBGM05, the lower 90% one-sided CI of EBG.

Supplemental table 4. Detailed demographic characteristics of patients with drug-associated pancreatitis events of 101 drugs.

| Drugs | ATC code | Total | Age | | | | | | | Gender | | | Weight (kg) | Reporting region | | | | | | |
| --- | --- | --- | --- | --- | --- | --- | --- | --- | --- | --- | --- | --- | --- | --- | --- | --- | --- | --- | --- | --- |
|  |  |  | <18y | 18–44y | 45–64y | 65–74y | 75–84y | ≥85y | Unknown | Female | Male | Unknown | Mean | Africa | Asian | Europe | North America | Oceania | South America | Unknown |
| Calcium carbonate | A02AC | 31 | 0(0.00) | 20(64.52) | 5(16.13) | 3(9.68) | 0(0.00) | 0(0.00) | 3(9.68) | 16(51.61) | 13(41.94) | 2(6.45) | 83 | 0(0.00) | 0(0.00) | 2(6.45) | 27(87.10) | 0(0.00) | 1(3.23) | 1(3.23) |
| Eluxadoline | A07DA | 350 | 0(0.00) | 63(18.00) | 90(25.71) | 25(7.14) | 16(4.57) | 4(1.14) | 152(43.43) | 258(73.71) | 57(16.29) | 35(10.00) | 88.16 | 0(0.00) | 0(0.00) | 0(0.00) | 350(100.00) | 0(0.00) | 0(0.00) | 0(0.00) |
| Balsalazide | A07EC | 9 | 1(11.11) | 4(44.44) | 1(11.11) | 1(11.11) | 0(0.00) | 0(0.00) | 2(22.22) | 3(33.33) | 4(44.44) | 2(22.22) | 55.4 | 0(0.00) | 0(0.00) | 1(11.11) | 7(77.78) | 0(0.00) | 0(0.00) | 1(11.11) |
| Mesalazine | A07EC | 319 | 24(7.52) | 109(34.17) | 30(9.40) | 8(2.51) | 2(0.63) | 0(0.00) | 146(45.77) | 99(31.03) | 128(40.13) | 92(28.84) | 74 | 1(0.31) | 51(15.99) | 82(25.71) | 171(53.61) | 5(1.57) | 1(0.31) | 8(2.51) |
| Olsalazine | A07EC | 3 | 0(0.00) | 3(100.00) | 0(0.00) | 0(0.00) | 0(0.00) | 0(0.00) | 0(0.00) | 1(33.33) | 1(33.33) | 1(33.33) | 83 | 0(0.00) | 0(0.00) | 0(0.00) | 1(33.33) | 0(0.00) | 0(0.00) | 2(66.67) |
| Pancrelipase amylase | A09AA | 400 | 4(1.00) | 26(6.50) | 88(22.00) | 52(13.00) | 23(5.75) | 6(1.50) | 201(50.25) | 213(53.25) | 166(41.50) | 21(5.25) | 66.62 | 2(0.50) | 8(2.00) | 9(2.25) | 373(93.25) | 2(0.50) | 4(1.00) | 2(0.50) |
| Metformin | A10BA | 1700 | 30(1.76) | 168(9.88) | 623(36.65) | 292(17.18) | 106(6.24) | 12(0.71) | 469(27.59) | 746(43.88) | 792(46.59) | 162(9.53) | 87.74 | 8(0.47) | 71(4.18) | 655(38.53) | 890(52.35) | 31(1.82) | 16(0.94) | 29(1.71) |
| Glimepiride | A10BB | 63 | 0(0.00) | 3(4.76) | 16(25.40) | 13(20.63) | 18(28.57) | 1(1.59) | 12(19.05) | 23(36.51) | 34(53.97) | 6(9.52) | 82.43 | 1(1.59) | 5(7.94) | 43(68.25) | 9(14.29) | 0(0.00) | 1(1.59) | 4(6.35) |
| Alogliptin | A10BH | 135 | 0(0.00) | 4(2.96) | 41(30.37) | 32(23.70) | 22(16.30) | 5(3.70) | 31(22.96) | 34(25.19) | 84(62.22) | 17(12.59) | 83.56 | 0(0.00) | 45(33.33) | 13(9.63) | 63(46.67) | 3(2.22) | 11(8.15) | 0(0.00) |
| Linagliptin | A10BH | 501 | 1(0.20) | 24(4.79) | 133(26.55) | 90(17.96) | 59(11.78) | 11(2.20) | 183(36.53) | 197(39.32) | 244(48.70) | 60(11.98) | 92.34 | 1(0.20) | 36(7.19) | 59(11.78) | 384(76.65) | 10(2.00) | 10(2.00) | 1(0.20) |
| Saxagliptin | A10BH | 330 | 1(0.30) | 13(3.94) | 122(36.97) | 64(19.39) | 26(7.88) | 3(0.91) | 101(30.61) | 110(33.33) | 167(50.61) | 53(16.06) | 96.53 | 1(0.30) | 21(6.36) | 59(17.88) | 235(71.21) | 4(1.21) | 10(3.03) | 0(0.00) |
| Sitagliptin | A10BH | 3167 | 4(0.13) | 106(3.35) | 721(22.77) | 408(12.88) | 209(6.60) | 47(1.48) | 1672(52.79) | 1216(38.40) | 1493(47.14) | 458(14.46) | 90.81 | 3(0.09) | 67(2.12) | 194(6.13) | 2881(90.97) | 8(0.25) | 14(0.44) | 0(0.00) |
| Albiglutide | A10BJ | 7 | 0(0.00) | 0(0.00) | 5(71.43) | 1(14.29) | 1(14.29) | 0(0.00) | 0(0.00) | 0(0.00) | 7(100.00) | 0(0.00) | 121.43 | 0(0.00) | 0(0.00) | 0(0.00) | 7(100.00) | 0(0.00) | 0(0.00) | 0(0.00) |
| Dulaglutide | A10BJ | 1415 | 1(0.07) | 53(3.75) | 375(26.50) | 140(9.89) | 46(3.25) | 7(0.49) | 793(56.04) | 558(39.43) | 587(41.48) | 270(19.08) | 102.95 | 1(0.07) | 19(1.34) | 69(4.88) | 1316(93.00) | 3(0.21) | 7(0.49) | 0(0.00) |
| Exenatide | A10BJ | 3210 | 2(0.06) | 185(5.76) | 1083(33.74) | 336(10.47) | 78(2.43) | 8(0.25) | 1518(47.29) | 1593(49.63) | 1519(47.32) | 98(3.05) | 103.27 | 7(0.22) | 56(1.74) | 289(9.00) | 2787(86.82) | 50(1.56) | 20(0.62) | 1(0.03) |
| Liraglutide | A10BJ | 2865 | 4(0.14) | 251(8.76) | 1086(37.91) | 384(13.40) | 67(2.34) | 4(0.14) | 1069(37.31) | 1401(48.90) | 1111(38.78) | 353(12.32) | 102.05 | 1(0.03) | 43(1.50) | 207(7.23) | 2568(89.63) | 5(0.17) | 39(1.36) | 2(0.07) |
| Lixisenatide | A10BJ | 36 | 0(0.00) | 1(2.78) | 12(33.33) | 3(8.33) | 2(5.56) | 0(0.00) | 18(50.00) | 13(36.11) | 11(30.56) | 12(33.33) | 123.33 | 0(0.00) | 4(11.11) | 5(13.89) | 25(69.44) | 0(0.00) | 2(5.56) | 0(0.00) |
| Semaglutide | A10BJ | 650 | 1(0.15) | 39(6.00) | 211(32.46) | 77(11.85) | 34(5.23) | 2(0.31) | 286(44.00) | 301(46.31) | 316(48.62) | 33(5.08) | 98.34 | 0(0.00) | 4(0.62) | 45(6.92) | 594(91.38) | 3(0.46) | 4(0.62) | 0(0.00) |
| Canagliflozin | A10BK | 360 | 0(0.00) | 57(15.83) | 142(39.44) | 36(10.00) | 11(3.06) | 0(0.00) | 114(31.67) | 143(39.72) | 167(46.39) | 50(13.89) | 94.88 | 0(0.00) | 6(1.67) | 8(2.22) | 346(96.11) | 0(0.00) | 0(0.00) | 0(0.00) |
| Dapagliflozin | A10BK | 259 | 0(0.00) | 22(8.49) | 107(41.31) | 30(11.58) | 5(1.93) | 2(0.77) | 93(35.91) | 102(39.38) | 128(49.42) | 29(11.20) | 94.17 | 2(0.77) | 19(7.34) | 64(24.71) | 153(59.07) | 9(3.47) | 12(4.63) | 0(0.00) |
| Empagliflozin | A10BK | 381 | 1(0.26) | 38(9.97) | 120(31.50) | 51(13.39) | 23(6.04) | 6(1.57) | 142(37.27) | 138(36.22) | 203(53.28) | 40(10.50) | 91.29 | 2(0.52) | 31(8.14) | 68(17.85) | 253(66.40) | 13(3.41) | 14(3.67) | 0(0.00) |
| Repaglinide | A10BX | 12 | 0(0.00) | 0(0.00) | 6(50.00) | 2(16.67) | 0(0.00) | 1(8.33) | 3(25.00) | 3(25.00) | 7(58.33) | 2(16.67) | 96.83 | 0(0.00) | 0(0.00) | 7(58.33) | 4(33.33) | 0(0.00) | 0(0.00) | 1(8.33) |
| Carglumic acid | A16AA | 16 | 10(62.50) | 1(6.25) | 0(0.00) | 0(0.00) | 0(0.00) | 0(0.00) | 5(31.25) | 2(12.50) | 11(68.75) | 3(18.75) | 30.6 | 0(0.00) | 1(6.25) | 2(12.50) | 13(81.25) | 0(0.00) | 0(0.00) | 0(0.00) |
| Levocarnitine | A16AA | 4 | 1(25.00) | 1(25.00) | 2(50.00) | 0(0.00) | 0(0.00) | 0(0.00) | 0(0.00) | 1(25.00) | 3(75.00) | 0(0.00) | 35 | 0(0.00) | 2(50.00) | 1(25.00) | 1(25.00) | 0(0.00) | 0(0.00) | 0(0.00) |
| Metreleptin | A16AA | 52 | 10(19.23) | 26(50.00) | 11(21.15) | 0(0.00) | 0(0.00) | 0(0.00) | 5(9.62) | 45(86.54) | 5(9.62) | 2(3.85) | 68.96 | 0(0.00) | 0(0.00) | 0(0.00) | 51(98.08) | 0(0.00) | 1(1.92) | 0(0.00) |
| Givosiran | A16AX | 13 | 0(0.00) | 0(0.00) | 0(0.00) | 0(0.00) | 0(0.00) | 0(0.00) | 13(100.00) | 0(0.00) | 0(0.00) | 13(100.00) |  | 0(0.00) | 0(0.00) | 4(30.77) | 9(69.23) | 0(0.00) | 0(0.00) | 0(0.00) |
| Teduglutide | A16AX | 101 | 1(0.99) | 17(16.83) | 27(26.73) | 11(10.89) | 5(4.95) | 0(0.00) | 40(39.60) | 63(62.38) | 27(26.73) | 11(10.89) | 59.46 | 0(0.00) | 4(3.96) | 13(12.87) | 83(82.18) | 1(0.99) | 0(0.00) | 0(0.00) |
| Hydrochlorothiazide | C03AA | 752 | 1(0.13) | 30(3.99) | 180(23.94) | 92(12.23) | 48(6.38) | 12(1.60) | 389(51.73) | 226(30.05) | 230(30.59) | 296(39.36) | 87.29 | 0(0.00) | 27(3.59) | 95(12.63) | 545(72.47) | 5(0.66) | 25(3.32) | 55(7.31) |
| Metolazone | C03BA | 11 | 0(0.00) | 1(9.09) | 6(54.55) | 1(9.09) | 1(9.09) | 2(18.18) | 0(0.00) | 4(36.36) | 7(63.64) | 0(0.00) | 114.89 | 0(0.00) | 0(0.00) | 0(0.00) | 10(90.91) | 0(0.00) | 0(0.00) | 1(9.09) |
| Enalapril | C09AA | 69 | 1(1.45) | 3(4.35) | 23(33.33) | 18(26.09) | 17(24.64) | 3(4.35) | 4(5.80) | 31(44.93) | 37(53.62) | 1(1.45) | 85.83 | 0(0.00) | 1(1.45) | 53(76.81) | 9(13.04) | 1(1.45) | 1(1.45) | 4(5.80) |
| Lisinopril | C09AA | 306 | 1(0.33) | 27(8.82) | 151(49.35) | 70(22.88) | 22(7.19) | 6(1.96) | 29(9.48) | 93(30.39) | 204(66.67) | 9(2.94) | 90.68 | 0(0.00) | 3(0.98) | 52(16.99) | 233(76.14) | 0(0.00) | 2(0.65) | 16(5.23) |
| Perindopril | C09AA | 40 | 0(0.00) | 3(7.50) | 20(50.00) | 9(22.50) | 1(2.50) | 1(2.50) | 6(15.00) | 11(27.50) | 25(62.50) | 4(10.00) | 97.73 | 0(0.00) | 0(0.00) | 27(67.50) | 9(22.50) | 0(0.00) | 1(2.50) | 3(7.50) |
| Trandolapril | C09AA | 14 | 0(0.00) | 0(0.00) | 4(28.57) | 3(21.43) | 3(21.43) | 0(0.00) | 4(28.57) | 8(57.14) | 6(42.86) | 0(0.00) | 84 | 0(0.00) | 0(0.00) | 2(14.29) | 3(21.43) | 0(0.00) | 0(0.00) | 9(64.29) |
| Candesartan | C09CA | 41 | 0(0.00) | 3(7.32) | 6(14.63) | 15(36.59) | 4(9.76) | 8(19.51) | 5(12.20) | 20(48.78) | 17(41.46) | 4(9.76) | 78.74 | 0(0.00) | 1(2.44) | 37(90.24) | 1(2.44) | 0(0.00) | 0(0.00) | 2(4.88) |
| Losartan | C09CA | 158 | 0(0.00) | 5(3.16) | 67(42.41) | 29(18.35) | 19(12.03) | 9(5.70) | 29(18.35) | 55(34.81) | 80(50.63) | 23(14.56) | 82.1 | 0(0.00) | 4(2.53) | 89(56.33) | 53(33.54) | 0(0.00) | 3(1.90) | 9(5.70) |
| Olmesartan | C09CA | 319 | 0(0.00) | 1(0.31) | 8(2.51) | 11(3.45) | 7(2.19) | 1(0.31) | 291(91.22) | 24(7.52) | 15(4.70) | 280(87.77) | 76.06 | 0(0.00) | 6(1.88) | 10(3.13) | 299(93.73) | 0(0.00) | 2(0.63) | 2(0.63) |
| Pravastatin | C10AA | 51 | 0(0.00) | 0(0.00) | 24(47.06) | 11(21.57) | 12(23.53) | 0(0.00) | 4(7.84) | 24(47.06) | 25(49.02) | 2(3.92) | 96.29 | 0(0.00) | 0(0.00) | 24(47.06) | 26(50.98) | 0(0.00) | 0(0.00) | 1(1.96) |
| Simvastatin | C10AA | 472 | 3(0.64) | 28(5.93) | 142(30.08) | 111(23.52) | 51(10.81) | 14(2.97) | 123(26.06) | 178(37.71) | 235(49.79) | 59(12.50) | 84.15 | 1(0.21) | 3(0.64) | 243(51.48) | 180(38.14) | 9(1.91) | 7(1.48) | 29(6.14) |
| Fenofibrate | C10AB | 158 | 9(5.70) | 27(17.09) | 48(30.38) | 25(15.82) | 2(1.27) | 4(2.53) | 43(27.22) | 51(32.28) | 93(58.86) | 14(8.86) | 86.4 | 0(0.00) | 5(3.16) | 63(39.87) | 77(48.73) | 1(0.63) | 0(0.00) | 12(7.59) |
| Fenofibric acid | C10AB | 51 | 0(0.00) | 3(5.88) | 8(15.69) | 9(17.65) | 0(0.00) | 0(0.00) | 31(60.78) | 11(21.57) | 26(50.98) | 14(27.45) | 96.76 | 0(0.00) | 0(0.00) | 0(0.00) | 50(98.04) | 0(0.00) | 0(0.00) | 1(1.96) |
| Bempedoic acid | C10AX | 12 | 0(0.00) | 0(0.00) | 4(33.33) | 2(16.67) | 2(16.67) | 0(0.00) | 4(33.33) | 6(50.00) | 4(33.33) | 2(16.67) | 80 | 0(0.00) | 0(0.00) | 5(41.67) | 7(58.33) | 0(0.00) | 0(0.00) | 0(0.00) |
| Ezetimibe | C10AX | 387 | 0(0.00) | 21(5.43) | 110(28.42) | 73(18.86) | 39(10.08) | 12(3.10) | 132(34.11) | 143(36.95) | 193(49.87) | 51(13.18) | 85.91 | 0(0.00) | 6(1.55) | 70(18.09) | 241(62.27) | 16(4.13) | 0(0.00) | 54(13.95) |
| Terbinafine | D01AE | 88 | 0(0.00) | 16(18.18) | 31(35.23) | 17(19.32) | 4(4.55) | 0(0.00) | 20(22.73) | 23(26.14) | 58(65.91) | 7(7.95) | 71 | 0(0.00) | 2(2.27) | 44(50.00) | 27(30.68) | 2(2.27) | 0(0.00) | 13(14.77) |
| Ethanol | D08AX | 57 | 0(0.00) | 7(12.28) | 17(29.82) | 9(15.79) | 3(5.26) | 0(0.00) | 21(36.84) | 33(57.89) | 18(31.58) | 6(10.53) | 69.92 | 0(0.00) | 0(0.00) | 2(3.51) | 55(96.49) | 0(0.00) | 0(0.00) | 0(0.00) |
| Drospirenone | G03AC | 589 | 41(6.96) | 469(79.63) | 26(4.41) | 1(0.17) | 0(0.00) | 0(0.00) | 52(8.83) | 582(98.81) | 3(0.51) | 4(0.68) | 78.48 | 0(0.00) | 16(2.72) | 36(6.11) | 525(89.13) | 3(0.51) | 3(0.51) | 6(1.02) |
| Norethisterone | G03AC | 226 | 1(0.44) | 23(10.18) | 68(30.09) | 39(17.26) | 17(7.52) | 2(0.88) | 76(33.63) | 85(37.61) | 100(44.25) | 41(18.14) | 91.8 | 0(0.00) | 10(4.42) | 44(19.47) | 162(71.68) | 3(1.33) | 6(2.65) | 1(0.44) |
| Equilin | G03CA | 3 | 0(0.00) | 0(0.00) | 1(33.33) | 2(66.67) | 0(0.00) | 0(0.00) | 0(0.00) | 3(100.00) | 0(0.00) | 0(0.00) | 44.67 | 0(0.00) | 0(0.00) | 0(0.00) | 3(100.00) | 0(0.00) | 0(0.00) | 0(0.00) |
| Thiamazole | H03BB | 9 | 0(0.00) | 0(0.00) | 2(22.22) | 2(22.22) | 2(22.22) | 0(0.00) | 3(33.33) | 6(66.67) | 1(11.11) | 2(22.22) | 43.67 | 0(0.00) | 4(44.44) | 2(22.22) | 3(33.33) | 0(0.00) | 0(0.00) | 0(0.00) |
| Doxercalciferol | H05BX | 9 | 0(0.00) | 0(0.00) | 3(33.33) | 1(11.11) | 0(0.00) | 0(0.00) | 5(55.56) | 3(33.33) | 1(11.11) | 5(55.56) | 82 | 0(0.00) | 0(0.00) | 0(0.00) | 8(88.89) | 0(0.00) | 0(0.00) | 1(11.11) |
| Doxycycline | J01AA | 285 | 1(0.35) | 68(23.86) | 91(31.93) | 19(6.67) | 51(17.89) | 16(5.61) | 39(13.68) | 174(61.05) | 87(30.53) | 24(8.42) | 79.36 | 0(0.00) | 21(7.37) | 83(29.12) | 175(61.40) | 2(0.70) | 0(0.00) | 4(1.40) |
| Tigecycline | J01AA | 179 | 11(6.15) | 31(17.32) | 41(22.91) | 24(13.41) | 15(8.38) | 3(1.68) | 54(30.17) | 93(51.96) | 64(35.75) | 22(12.29) | 77.67 | 2(1.12) | 9(5.03) | 25(13.97) | 140(78.21) | 0(0.00) | 2(1.12) | 1(0.56) |
| Cefpodoxime | J01DD | 9 | 1(11.11) | 5(55.56) | 2(22.22) | 0(0.00) | 0(0.00) | 0(0.00) | 1(11.11) | 3(33.33) | 5(55.56) | 1(11.11) | 79 | 0(0.00) | 0(0.00) | 8(88.89) | 0(0.00) | 0(0.00) | 0(0.00) | 1(11.11) |
| Metronidazole | J01XD | 180 | 17(9.44) | 55(30.56) | 40(22.22) | 20(11.11) | 4(2.22) | 11(6.11) | 33(18.33) | 98(54.44) | 49(27.22) | 33(18.33) | 73.7 | 1(0.56) | 14(7.78) | 47(26.11) | 95(52.78) | 5(2.78) | 0(0.00) | 18(10.00) |
| Tinidazole | J01XD | 4 | 0(0.00) | 0(0.00) | 4(100.00) | 0(0.00) | 0(0.00) | 0(0.00) | 0(0.00) | 4(100.00) | 0(0.00) | 0(0.00) | 99 | 0(0.00) | 2(50.00) | 0(0.00) | 2(50.00) | 0(0.00) | 0(0.00) | 0(0.00) |
| Linezolid | J01XX | 128 | 12(9.38) | 32(25.00) | 29(22.66) | 19(14.84) | 5(3.91) | 8(6.25) | 23(17.97) | 67(52.34) | 43(33.59) | 18(14.06) | 74.47 | 3(2.34) | 8(6.25) | 74(57.81) | 34(26.56) | 0(0.00) | 2(1.56) | 7(5.47) |
| Rifampicin | J04AB | 59 | 7(11.86) | 7(11.86) | 13(22.03) | 8(13.56) | 4(6.78) | 7(11.86) | 13(22.03) | 27(45.76) | 22(37.29) | 10(16.95) | 52.23 | 1(1.69) | 11(18.64) | 32(54.24) | 5(8.47) | 0(0.00) | 0(0.00) | 10(16.95) |
| Isoniazid | J04AC | 70 | 6(8.57) | 19(27.14) | 11(15.71) | 11(15.71) | 7(10.00) | 11(15.71) | 5(7.14) | 46(65.71) | 23(32.86) | 1(1.43) | 62.57 | 2(2.86) | 11(15.71) | 18(25.71) | 31(44.29) | 0(0.00) | 0(0.00) | 8(11.43) |
| Bedaquiline | J04AK | 23 | 0(0.00) | 10(43.48) | 6(26.09) | 0(0.00) | 1(4.35) | 0(0.00) | 6(26.09) | 7(30.43) | 12(52.17) | 4(17.39) | 55 | 6(26.09) | 6(26.09) | 10(43.48) | 1(4.35) | 0(0.00) | 0(0.00) | 0(0.00) |
| Pyrazinamide | J04AK | 11 | 0(0.00) | 3(27.27) | 2(18.18) | 0(0.00) | 1(9.09) | 3(27.27) | 2(18.18) | 7(63.64) | 3(27.27) | 1(9.09) |  | 2(18.18) | 0(0.00) | 3(27.27) | 1(9.09) | 0(0.00) | 0(0.00) | 5(45.45) |
| Foscarnet | J05AD | 11 | 0(0.00) | 4(36.36) | 3(27.27) | 0(0.00) | 0(0.00) | 0(0.00) | 4(36.36) | 2(18.18) | 8(72.73) | 1(9.09) | 56.33 | 0(0.00) | 1(9.09) | 2(18.18) | 7(63.64) | 0(0.00) | 0(0.00) | 1(9.09) |
| Atazanavir | J05AE | 57 | 0(0.00) | 19(33.33) | 23(40.35) | 2(3.51) | 0(0.00) | 0(0.00) | 13(22.81) | 11(19.30) | 40(70.18) | 6(10.53) | 62.08 | 0(0.00) | 2(3.51) | 19(33.33) | 22(38.60) | 0(0.00) | 1(1.75) | 13(22.81) |
| Fosamprenavir | J05AE | 10 | 0(0.00) | 2(20.00) | 6(60.00) | 0(0.00) | 0(0.00) | 0(0.00) | 2(20.00) | 0(0.00) | 10(100.00) | 0(0.00) | 79 | 0(0.00) | 1(10.00) | 4(40.00) | 4(40.00) | 0(0.00) | 0(0.00) | 1(10.00) |
| Indinavir | J05AE | 8 | 0(0.00) | 5(62.50) | 0(0.00) | 0(0.00) | 1(12.50) | 0(0.00) | 2(25.00) | 1(12.50) | 7(87.50) | 0(0.00) | 79 | 0(0.00) | 0(0.00) | 0(0.00) | 3(37.50) | 0(0.00) | 0(0.00) | 5(62.50) |
| Abacavir | J05AF | 101 | 10(9.90) | 28(27.72) | 30(29.70) | 4(3.96) | 0(0.00) | 0(0.00) | 29(28.71) | 23(22.77) | 69(68.32) | 9(8.91) | 67.05 | 2(1.98) | 13(12.87) | 32(31.68) | 42(41.58) | 2(1.98) | 1(0.99) | 9(8.91) |
| Didanosine | J05AF | 51 | 0(0.00) | 26(50.98) | 16(31.37) | 2(3.92) | 1(1.96) | 0(0.00) | 6(11.76) | 23(45.10) | 27(52.94) | 1(1.96) | 64.57 | 0(0.00) | 0(0.00) | 19(37.25) | 15(29.41) | 0(0.00) | 1(1.96) | 16(31.37) |
| Stavudine | J05AF | 51 | 7(13.73) | 21(41.18) | 15(29.41) | 0(0.00) | 0(0.00) | 0(0.00) | 8(15.69) | 15(29.41) | 32(62.75) | 4(7.84) | 62.08 | 1(1.96) | 12(23.53) | 14(27.45) | 12(23.53) | 0(0.00) | 0(0.00) | 12(23.53) |
| Nevirapine | J05AG | 66 | 9(13.64) | 15(22.73) | 13(19.70) | 1(1.52) | 0(0.00) | 0(0.00) | 28(42.42) | 20(30.30) | 32(48.48) | 14(21.21) | 73.83 | 2(3.03) | 5(7.58) | 11(16.67) | 17(25.76) | 0(0.00) | 0(0.00) | 31(46.97) |
| Raltegravir | J05AJ | 65 | 3(4.62) | 20(30.77) | 28(43.08) | 0(0.00) | 1(1.54) | 0(0.00) | 13(20.00) | 27(41.54) | 35(53.85) | 3(4.62) | 74.09 | 3(4.62) | 6(9.23) | 35(53.85) | 16(24.62) | 0(0.00) | 5(7.69) | 0(0.00) |
| Enfuvirtide | J05AX | 27 | 0(0.00) | 7(25.93) | 6(22.22) | 0(0.00) | 0(0.00) | 0(0.00) | 14(51.85) | 5(18.52) | 21(77.78) | 1(3.70) | 73.57 | 0(0.00) | 0(0.00) | 2(7.41) | 15(55.56) | 0(0.00) | 0(0.00) | 10(37.04) |
| Clofarabine | L01BB | 41 | 20(48.78) | 12(29.27) | 3(7.32) | 2(4.88) | 1(2.44) | 0(0.00) | 3(7.32) | 26(63.41) | 15(36.59) | 0(0.00) | 56.43 | 0(0.00) | 4(9.76) | 5(12.20) | 31(75.61) | 0(0.00) | 0(0.00) | 1(2.44) |
| Mercaptopurine | L01BB | 128 | 45(35.16) | 23(17.97) | 26(20.31) | 4(3.13) | 1(0.78) | 0(0.00) | 29(22.66) | 50(39.06) | 62(48.44) | 16(12.50) | 56.76 | 1(0.78) | 16(12.50) | 16(12.50) | 89(69.53) | 0(0.00) | 0(0.00) | 6(4.69) |
| Tioguanine | L01BB | 6 | 1(16.67) | 2(33.33) | 0(0.00) | 0(0.00) | 0(0.00) | 0(0.00) | 3(50.00) | 5(83.33) | 1(16.67) | 0(0.00) | 42 | 0(0.00) | 0(0.00) | 5(83.33) | 0(0.00) | 1(16.67) | 0(0.00) | 0(0.00) |
| Vincristine | L01CA | 71 | 39(54.93) | 10(14.08) | 7(9.86) | 1(1.41) | 1(1.41) | 0(0.00) | 13(18.31) | 28(39.44) | 41(57.75) | 2(2.82) | 54.2 | 0(0.00) | 3(4.23) | 39(54.93) | 28(39.44) | 0(0.00) | 1(1.41) | 0(0.00) |
| Daunorubicin | L01DB | 27 | 8(29.63) | 5(18.52) | 3(11.11) | 3(11.11) | 0(0.00) | 0(0.00) | 8(29.63) | 18(66.67) | 9(33.33) | 0(0.00) | 70.43 | 0(0.00) | 0(0.00) | 2(7.41) | 22(81.48) | 0(0.00) | 0(0.00) | 3(11.11) |
| Lenvatinib | L01EX | 70 | 2(2.86) | 6(8.57) | 31(44.29) | 20(28.57) | 8(11.43) | 0(0.00) | 3(4.29) | 26(37.14) | 44(62.86) | 0(0.00) | 74.73 | 0(0.00) | 18(25.71) | 30(42.86) | 16(22.86) | 2(2.86) | 4(5.71) | 0(0.00) |
| Brentuximab vedotin | L01FX | 89 | 1(1.12) | 15(16.85) | 18(20.22) | 13(14.61) | 4(4.49) | 0(0.00) | 38(42.70) | 27(30.34) | 42(47.19) | 20(22.47) | 85.48 | 0(0.00) | 14(15.73) | 21(23.60) | 52(58.43) | 0(0.00) | 2(2.25) | 0(0.00) |
| Nilotinib | L01XE | 448 | 1(0.22) | 31(6.92) | 106(23.66) | 58(12.95) | 28(6.25) | 7(1.56) | 217(48.44) | 168(37.50) | 213(47.54) | 67(14.96) | 88.65 | 1(0.22) | 23(5.13) | 58(12.95) | 351(78.35) | 5(1.12) | 10(2.23) | 0(0.00) |
| Ponatinib | L01XE | 182 | 1(0.55) | 24(13.19) | 43(23.63) | 26(14.29) | 14(7.69) | 0(0.00) | 74(40.66) | 68(37.36) | 100(54.95) | 14(7.69) | 87.15 | 0(0.00) | 10(5.49) | 18(9.89) | 148(81.32) | 1(0.55) | 4(2.20) | 1(0.55) |
| Arsenic trioxide | L01XX | 14 | 1(7.14) | 3(21.43) | 5(35.71) | 3(21.43) | 0(0.00) | 0(0.00) | 2(14.29) | 9(64.29) | 3(21.43) | 2(14.29) | 88.13 | 0(0.00) | 2(14.29) | 0(0.00) | 8(57.14) | 0(0.00) | 0(0.00) | 4(28.57) |
| Asparaginase | L01XX | 163 | 85(52.15) | 20(12.27) | 2(1.23) | 1(0.61) | 1(0.61) | 0(0.00) | 54(33.13) | 55(33.74) | 75(46.01) | 33(20.25) | 57.95 | 1(0.61) | 29(17.79) | 22(13.50) | 103(63.19) | 0(0.00) | 1(0.61) | 7(4.29) |
| Pegaspargase | L01XX | 267 | 70(26.22) | 36(13.48) | 2(0.75) | 0(0.00) | 0(0.00) | 0(0.00) | 159(59.55) | 51(19.10) | 70(26.22) | 146(54.68) | 59.57 | 0(0.00) | 6(2.25) | 99(37.08) | 145(54.31) | 2(0.75) | 6(2.25) | 9(3.37) |
| Basiliximab | L04AC | 21 | 2(9.52) | 9(42.86) | 5(23.81) | 2(9.52) | 1(4.76) | 0(0.00) | 2(9.52) | 5(23.81) | 14(66.67) | 2(9.52) | 60.75 | 0(0.00) | 6(28.57) | 11(52.38) | 2(9.52) | 0(0.00) | 0(0.00) | 2(9.52) |
| Azathioprine | L04AX | 238 | 14(5.88) | 88(36.97) | 60(25.21) | 16(6.72) | 5(2.10) | 0(0.00) | 55(23.11) | 123(51.68) | 81(34.03) | 34(14.29) | 78.44 | 0(0.00) | 11(4.62) | 98(41.18) | 113(47.48) | 3(1.26) | 0(0.00) | 13(5.46) |
| Flurbiprofen | M01AE | 5 | 2(40.00) | 1(20.00) | 2(40.00) | 0(0.00) | 0(0.00) | 0(0.00) | 0(0.00) | 5(100.00) | 0(0.00) | 0(0.00) |  | 0(0.00) | 0(0.00) | 5(100.00) | 0(0.00) | 0(0.00) | 0(0.00) | 0(0.00) |
| Ketoprofen | M01AE | 17 | 2(11.76) | 4(23.53) | 4(23.53) | 3(17.65) | 1(5.88) | 3(17.65) | 0(0.00) | 8(47.06) | 9(52.94) | 0(0.00) | 69.7 | 0(0.00) | 0(0.00) | 16(94.12) | 1(5.88) | 0(0.00) | 0(0.00) | 0(0.00) |
| Propofol | N01AX | 92 | 4(4.35) | 31(33.70) | 37(40.22) | 13(14.13) | 3(3.26) | 2(2.17) | 2(2.17) | 38(41.30) | 53(57.61) | 1(1.09) | 73.44 | 0(0.00) | 4(4.35) | 36(39.13) | 41(44.57) | 2(2.17) | 0(0.00) | 9(9.78) |
| Codeine | N02AA | 77 | 5(6.49) | 27(35.06) | 18(23.38) | 16(20.78) | 3(3.90) | 3(3.90) | 5(6.49) | 31(40.26) | 31(40.26) | 15(19.48) | 83.69 | 0(0.00) | 0(0.00) | 48(62.34) | 10(12.99) | 10(12.99) | 1(1.30) | 8(10.39) |
| Eslicarbazepine | N03AF | 8 | 1(12.50) | 0(0.00) | 1(12.50) | 0(0.00) | 0(0.00) | 0(0.00) | 6(75.00) | 2(25.00) | 1(12.50) | 5(62.50) | 40 | 0(0.00) | 0(0.00) | 8(100.00) | 0(0.00) | 0(0.00) | 0(0.00) | 0(0.00) |
| Rufinamide | N03AF | 6 | 3(50.00) | 3(50.00) | 0(0.00) | 0(0.00) | 0(0.00) | 0(0.00) | 0(0.00) | 3(50.00) | 3(50.00) | 0(0.00) | 40 | 0(0.00) | 1(16.67) | 3(50.00) | 2(33.33) | 0(0.00) | 0(0.00) | 0(0.00) |
| Valproic acid | N03AG | 440 | 107(24.32) | 100(22.73) | 83(18.86) | 17(3.86) | 2(0.45) | 2(0.45) | 129(29.32) | 167(37.95) | 237(53.86) | 36(8.18) | 71.77 | 2(0.45) | 30(6.82) | 115(26.14) | 213(48.41) | 1(0.23) | 9(2.05) | 70(15.91) |
| Fluphenazine | N05AB | 7 | 0(0.00) | 7(100.00) | 0(0.00) | 0(0.00) | 0(0.00) | 0(0.00) | 0(0.00) | 0(0.00) | 7(100.00) | 0(0.00) | 75 | 0(0.00) | 1(14.29) | 5(71.43) | 1(14.29) | 0(0.00) | 0(0.00) | 0(0.00) |
| Olanzapine | N05AH | 1459 | 17(1.17) | 491(33.65) | 317(21.73) | 36(2.47) | 10(0.69) | 2(0.14) | 586(40.16) | 612(41.95) | 668(45.78) | 179(12.27) | 87.81 | 1(0.07) | 26(1.78) | 94(6.44) | 1228(84.17) | 6(0.41) | 2(0.14) | 102(6.99) |
| Quetiapine | N05AH | 3459 | 42(1.21) | 902(26.08) | 734(21.22) | 33(0.95) | 17(0.49) | 5(0.14) | 1726(49.90) | 1822(52.67) | 1490(43.08) | 147(4.25) | 91.01 | 0(0.00) | 22(0.64) | 149(4.31) | 3275(94.68) | 2(0.06) | 4(0.12) | 7(0.20) |
| Meprobamate | N05BC | 3 | 0(0.00) | 3(100.00) | 0(0.00) | 0(0.00) | 0(0.00) | 0(0.00) | 0(0.00) | 3(100.00) | 0(0.00) | 0(0.00) |  | 3(100.00) | 0(0.00) | 0(0.00) | 0(0.00) | 0(0.00) | 0(0.00) | 0(0.00) |
| Riluzole | N07XX | 59 | 0(0.00) | 1(1.69) | 12(20.34) | 26(44.07) | 8(13.56) | 0(0.00) | 12(20.34) | 16(27.12) | 39(66.10) | 4(6.78) | 67.29 | 0(0.00) | 5(8.47) | 41(69.49) | 11(18.64) | 0(0.00) | 0(0.00) | 2(3.39) |
| Miltefosine | P01CX | 3 | 2(66.67) | 1(33.33) | 0(0.00) | 0(0.00) | 0(0.00) | 0(0.00) | 0(0.00) | 1(33.33) | 2(66.67) | 0(0.00) |  | 0(0.00) | 0(0.00) | 1(33.33) | 2(66.67) | 0(0.00) | 0(0.00) | 0(0.00) |
| Pentamidine isethionate | P01CX | 6 | 0(0.00) | 1(16.67) | 2(33.33) | 0(0.00) | 0(0.00) | 0(0.00) | 3(50.00) | 1(16.67) | 3(50.00) | 2(33.33) | 59.33 | 0(0.00) | 0(0.00) | 0(0.00) | 6(100.00) | 0(0.00) | 0(0.00) | 0(0.00) |
| Calcium acetate | V03AE | 9 | 0(0.00) | 2(22.22) | 3(33.33) | 0(0.00) | 0(0.00) | 0(0.00) | 4(44.44) | 3(33.33) | 4(44.44) | 2(22.22) | 93 | 0(0.00) | 0(0.00) | 0(0.00) | 7(77.78) | 0(0.00) | 0(0.00) | 2(22.22) |
| Secretin | V04CK | 4 | 0(0.00) | 2(50.00) | 2(50.00) | 0(0.00) | 0(0.00) | 0(0.00) | 0(0.00) | 4(100.00) | 0(0.00) | 0(0.00) | 84 | 0(0.00) | 0(0.00) | 0(0.00) | 4(100.00) | 0(0.00) | 0(0.00) | 0(0.00) |
| Iodixanol | V08AB | 35 | 0(0.00) | 2(5.71) | 9(25.71) | 9(25.71) | 7(20.00) | 4(11.43) | 4(11.43) | 12(34.29) | 22(62.86) | 1(2.86) | 52.48 | 0(0.00) | 27(77.14) | 2(5.71) | 3(8.57) | 0(0.00) | 0(0.00) | 3(8.57) |
| Iothalamic acid | V09CX | 7 | 0(0.00) | 0(0.00) | 0(0.00) | 0(0.00) | 0(0.00) | 0(0.00) | 7(100.00) | 0(0.00) | 0(0.00) | 7(100.00) |  | 0(0.00) | 7(100.00) | 0(0.00) | 0(0.00) | 0(0.00) | 0(0.00) | 0(0.00) |

Supplemental table 4. (Continued) Detailed demographic characteristics of patients with drug-associated pancreatitis events of 101 drugs.

| Drugs | ATC code | Reporting year | | | | | | | | | | | | | | | | | | | | | |
| --- | --- | --- | --- | --- | --- | --- | --- | --- | --- | --- | --- | --- | --- | --- | --- | --- | --- | --- | --- | --- | --- | --- | --- |
|  |  | 2003 | 2004 | 2005 | 2006 | 2007 | 2008 | 2009 | 2010 | 2011 | 2012 | 2013 | 2014 | 2015 | 2016 | 2017 | 2018 | 2019 | 2020 | 2021 | 2022 | 2023 | Unknown |
| Calcium carbonate | A02AC | 0(0.00) | 0(0.00) | 1(3.23) | 0(0.00) | 1(3.23) | 1(3.23) | 1(3.23) | 1(3.23) | 1(3.23) | 0(0.00) | 3(9.68) | 1(3.23) | 2(6.45) | 1(3.23) | 1(3.23) | 4(12.90) | 3(9.68) | 5(16.13) | 3(9.68) | 1(3.23) | 1(3.23) | 0(0.00) |
| Eluxadoline | A07DA | 0(0.00) | 0(0.00) | 0(0.00) | 0(0.00) | 0(0.00) | 0(0.00) | 0(0.00) | 0(0.00) | 0(0.00) | 0(0.00) | 0(0.00) | 0(0.00) | 0(0.00) | 119(34.00) | 134(38.29) | 38(10.86) | 41(11.71) | 12(3.43) | 3(0.86) | 3(0.86) | 0(0.00) | 0(0.00) |
| Balsalazide | A07EC | 0(0.00) | 1(11.11) | 0(0.00) | 1(11.11) | 4(44.44) | 0(0.00) | 0(0.00) | 1(11.11) | 0(0.00) | 0(0.00) | 1(11.11) | 0(0.00) | 0(0.00) | 1(11.11) | 0(0.00) | 0(0.00) | 0(0.00) | 0(0.00) | 0(0.00) | 0(0.00) | 0(0.00) | 0(0.00) |
| Mesalazine | A07EC | 0(0.00) | 5(1.57) | 4(1.25) | 30(9.40) | 14(4.39) | 9(2.82) | 14(4.39) | 15(4.70) | 11(3.45) | 11(3.45) | 12(3.76) | 26(8.15) | 11(3.45) | 13(4.08) | 17(5.33) | 21(6.58) | 23(7.21) | 44(13.79) | 25(7.84) | 14(4.39) | 0(0.00) | 0(0.00) |
| Olsalazine | A07EC | 0(0.00) | 2(66.67) | 0(0.00) | 0(0.00) | 1(33.33) | 0(0.00) | 0(0.00) | 0(0.00) | 0(0.00) | 0(0.00) | 0(0.00) | 0(0.00) | 0(0.00) | 0(0.00) | 0(0.00) | 0(0.00) | 0(0.00) | 0(0.00) | 0(0.00) | 0(0.00) | 0(0.00) | 0(0.00) |
| Pancrelipase amylase | A09AA | 0(0.00) | 2(0.50) | 1(0.25) | 0(0.00) | 0(0.00) | 0(0.00) | 2(0.50) | 3(0.75) | 5(1.25) | 7(1.75) | 13(3.25) | 21(5.25) | 15(3.75) | 25(6.25) | 13(3.25) | 40(10.00) | 70(17.50) | 53(13.25) | 58(14.50) | 58(14.50) | 14(3.50) | 0(0.00) |
| Metformin | A10BA | 0(0.00) | 21(1.24) | 4(0.24) | 10(0.59) | 10(0.59) | 34(2.00) | 47(2.76) | 52(3.06) | 97(5.71) | 139(8.18) | 189(11.12) | 150(8.82) | 100(5.88) | 145(8.53) | 110(6.47) | 154(9.06) | 130(7.65) | 137(8.06) | 88(5.18) | 62(3.65) | 18(1.06) | 3(0.18) |
| Glimepiride | A10BB | 0(0.00) | 1(1.59) | 1(1.59) | 1(1.59) | 1(1.59) | 4(6.35) | 1(1.59) | 2(3.17) | 3(4.76) | 2(3.17) | 1(1.59) | 3(4.76) | 3(4.76) | 10(15.87) | 2(3.17) | 13(20.63) | 12(19.05) | 1(1.59) | 2(3.17) | 0(0.00) | 0(0.00) | 0(0.00) |
| Alogliptin | A10BH | 0(0.00) | 0(0.00) | 0(0.00) | 0(0.00) | 0(0.00) | 0(0.00) | 0(0.00) | 0(0.00) | 0(0.00) | 0(0.00) | 21(15.56) | 13(9.63) | 13(9.63) | 16(11.85) | 9(6.67) | 15(11.11) | 10(7.41) | 13(9.63) | 16(11.85) | 7(5.19) | 1(0.74) | 1(0.74) |
| Linagliptin | A10BH | 0(0.00) | 0(0.00) | 0(0.00) | 0(0.00) | 0(0.00) | 0(0.00) | 0(0.00) | 0(0.00) | 0(0.00) | 61(12.18) | 89(17.76) | 74(14.77) | 86(17.17) | 57(11.38) | 43(8.58) | 30(5.99) | 23(4.59) | 14(2.79) | 14(2.79) | 5(1.00) | 5(1.00) | 0(0.00) |
| Saxagliptin | A10BH | 0(0.00) | 0(0.00) | 0(0.00) | 0(0.00) | 1(0.30) | 0(0.00) | 0(0.00) | 10(3.03) | 66(20.00) | 57(17.27) | 66(20.00) | 46(13.94) | 31(9.39) | 20(6.06) | 11(3.33) | 10(3.03) | 3(0.91) | 2(0.61) | 3(0.91) | 3(0.91) | 0(0.00) | 1(0.30) |
| Sitagliptin | A10BH | 0(0.00) | 0(0.00) | 0(0.00) | 0(0.00) | 28(0.88) | 52(1.64) | 148(4.67) | 301(9.50) | 264(8.34) | 341(10.77) | 462(14.59) | 401(12.66) | 379(11.97) | 278(8.78) | 191(6.03) | 110(3.47) | 74(2.34) | 74(2.34) | 24(0.76) | 19(0.60) | 11(0.35) | 10(0.32) |
| Albiglutide | A10BJ | 0(0.00) | 0(0.00) | 0(0.00) | 0(0.00) | 0(0.00) | 0(0.00) | 0(0.00) | 0(0.00) | 0(0.00) | 0(0.00) | 0(0.00) | 0(0.00) | 0(0.00) | 3(42.86) | 2(28.57) | 1(14.29) | 1(14.29) | 0(0.00) | 0(0.00) | 0(0.00) | 0(0.00) | 0(0.00) |
| Dulaglutide | A10BJ | 0(0.00) | 0(0.00) | 0(0.00) | 0(0.00) | 0(0.00) | 0(0.00) | 0(0.00) | 0(0.00) | 0(0.00) | 0(0.00) | 0(0.00) | 0(0.00) | 45(3.18) | 146(10.32) | 196(13.85) | 197(13.92) | 211(14.91) | 215(15.19) | 210(14.84) | 174(12.30) | 20(1.41) | 1(0.07) |
| Exenatide | A10BJ | 0(0.00) | 0(0.00) | 14(0.44) | 36(1.12) | 89(2.77) | 305(9.50) | 505(15.73) | 428(13.33) | 748(23.30) | 200(6.23) | 254(7.91) | 100(3.12) | 176(5.48) | 96(2.99) | 85(2.65) | 63(1.96) | 68(2.12) | 22(0.69) | 10(0.31) | 6(0.19) | 1(0.03) | 4(0.12) |
| Liraglutide | A10BJ | 0(0.00) | 0(0.00) | 0(0.00) | 0(0.00) | 1(0.03) | 0(0.00) | 0(0.00) | 212(7.40) | 488(17.03) | 272(9.49) | 263(9.18) | 379(13.23) | 296(10.33) | 259(9.04) | 143(4.99) | 218(7.61) | 116(4.05) | 98(3.42) | 50(1.75) | 42(1.47) | 19(0.66) | 9(0.31) |
| Lixisenatide | A10BJ | 0(0.00) | 0(0.00) | 0(0.00) | 0(0.00) | 0(0.00) | 0(0.00) | 0(0.00) | 0(0.00) | 0(0.00) | 0(0.00) | 0(0.00) | 0(0.00) | 0(0.00) | 0(0.00) | 7(19.44) | 7(19.44) | 9(25.00) | 6(16.67) | 3(8.33) | 4(11.11) | 0(0.00) | 0(0.00) |
| Semaglutide | A10BJ | 0(0.00) | 0(0.00) | 0(0.00) | 0(0.00) | 0(0.00) | 0(0.00) | 0(0.00) | 0(0.00) | 0(0.00) | 0(0.00) | 0(0.00) | 0(0.00) | 0(0.00) | 0(0.00) | 0(0.00) | 30(4.62) | 79(12.15) | 145(22.31) | 153(23.54) | 163(25.08) | 80(12.31) | 0(0.00) |
| Canagliflozin | A10BK | 0(0.00) | 0(0.00) | 0(0.00) | 0(0.00) | 0(0.00) | 0(0.00) | 0(0.00) | 0(0.00) | 0(0.00) | 0(0.00) | 1(0.28) | 19(5.28) | 118(32.78) | 76(21.11) | 69(19.17) | 53(14.72) | 5(1.39) | 7(1.94) | 9(2.50) | 2(0.56) | 1(0.28) | 0(0.00) |
| Dapagliflozin | A10BK | 0(0.00) | 0(0.00) | 0(0.00) | 0(0.00) | 0(0.00) | 0(0.00) | 0(0.00) | 0(0.00) | 0(0.00) | 0(0.00) | 0(0.00) | 6(2.32) | 21(8.11) | 30(11.58) | 28(10.81) | 40(15.44) | 36(13.90) | 24(9.27) | 26(10.04) | 33(12.74) | 15(5.79) | 0(0.00) |
| Empagliflozin | A10BK | 0(0.00) | 0(0.00) | 0(0.00) | 0(0.00) | 0(0.00) | 0(0.00) | 0(0.00) | 0(0.00) | 0(0.00) | 0(0.00) | 0(0.00) | 0(0.00) | 17(4.46) | 27(7.09) | 46(12.07) | 46(12.07) | 68(17.85) | 37(9.71) | 69(18.11) | 49(12.86) | 22(5.77) | 0(0.00) |
| Repaglinide | A10BX | 0(0.00) | 1(8.33) | 0(0.00) | 0(0.00) | 0(0.00) | 0(0.00) | 0(0.00) | 0(0.00) | 0(0.00) | 1(8.33) | 1(8.33) | 2(16.67) | 0(0.00) | 0(0.00) | 0(0.00) | 1(8.33) | 4(33.33) | 1(8.33) | 1(8.33) | 0(0.00) | 0(0.00) | 0(0.00) |
| Carglumic acid | A16AA | 0(0.00) | 0(0.00) | 0(0.00) | 0(0.00) | 0(0.00) | 0(0.00) | 0(0.00) | 0(0.00) | 0(0.00) | 1(6.25) | 2(12.50) | 0(0.00) | 0(0.00) | 0(0.00) | 2(12.50) | 5(31.25) | 2(12.50) | 1(6.25) | 0(0.00) | 3(18.75) | 0(0.00) | 0(0.00) |
| Levocarnitine | A16AA | 0(0.00) | 0(0.00) | 0(0.00) | 0(0.00) | 0(0.00) | 0(0.00) | 0(0.00) | 0(0.00) | 1(25.00) | 0(0.00) | 0(0.00) | 0(0.00) | 1(25.00) | 0(0.00) | 2(50.00) | 0(0.00) | 0(0.00) | 0(0.00) | 0(0.00) | 0(0.00) | 0(0.00) | 0(0.00) |
| Metreleptin | A16AA | 0(0.00) | 0(0.00) | 0(0.00) | 0(0.00) | 0(0.00) | 0(0.00) | 0(0.00) | 0(0.00) | 0(0.00) | 0(0.00) | 0(0.00) | 0(0.00) | 0(0.00) | 5(9.62) | 6(11.54) | 7(13.46) | 12(23.08) | 12(23.08) | 1(1.92) | 3(5.77) | 6(11.54) | 0(0.00) |
| Givosiran | A16AX | 0(0.00) | 0(0.00) | 0(0.00) | 0(0.00) | 0(0.00) | 0(0.00) | 0(0.00) | 0(0.00) | 0(0.00) | 0(0.00) | 0(0.00) | 0(0.00) | 0(0.00) | 0(0.00) | 0(0.00) | 0(0.00) | 0(0.00) | 1(7.69) | 2(15.38) | 8(61.54) | 2(15.38) | 0(0.00) |
| Teduglutide | A16AX | 0(0.00) | 0(0.00) | 0(0.00) | 0(0.00) | 0(0.00) | 0(0.00) | 0(0.00) | 0(0.00) | 0(0.00) | 0(0.00) | 1(0.99) | 10(9.90) | 9(8.91) | 5(4.95) | 4(3.96) | 10(9.90) | 9(8.91) | 9(8.91) | 16(15.84) | 20(19.80) | 8(7.92) | 0(0.00) |
| Hydrochlorothiazide | C03AA | 0(0.00) | 20(2.66) | 29(3.86) | 26(3.46) | 32(4.26) | 31(4.12) | 35(4.65) | 28(3.72) | 20(2.66) | 28(3.72) | 27(3.59) | 10(1.33) | 27(3.59) | 27(3.59) | 19(2.53) | 181(24.07) | 125(16.62) | 46(6.12) | 19(2.53) | 21(2.79) | 1(0.13) | 0(0.00) |
| Metolazone | C03BA | 0(0.00) | 0(0.00) | 1(9.09) | 1(9.09) | 0(0.00) | 1(9.09) | 0(0.00) | 3(27.27) | 2(18.18) | 1(9.09) | 1(9.09) | 0(0.00) | 0(0.00) | 0(0.00) | 0(0.00) | 1(9.09) | 0(0.00) | 0(0.00) | 0(0.00) | 0(0.00) | 0(0.00) | 0(0.00) |
| Enalapril | C09AA | 0(0.00) | 3(4.35) | 4(5.80) | 0(0.00) | 4(5.80) | 1(1.45) | 8(11.59) | 1(1.45) | 2(2.90) | 3(4.35) | 3(4.35) | 3(4.35) | 2(2.90) | 2(2.90) | 3(4.35) | 8(11.59) | 5(7.25) | 6(8.70) | 10(14.49) | 1(1.45) | 0(0.00) | 0(0.00) |
| Lisinopril | C09AA | 0(0.00) | 6(1.96) | 12(3.92) | 8(2.61) | 19(6.21) | 16(5.23) | 14(4.58) | 24(7.84) | 18(5.88) | 19(6.21) | 16(5.23) | 14(4.58) | 12(3.92) | 14(4.58) | 16(5.23) | 10(3.27) | 16(5.23) | 27(8.82) | 35(11.44) | 7(2.29) | 3(0.98) | 0(0.00) |
| Perindopril | C09AA | 0(0.00) | 3(7.50) | 1(2.50) | 2(5.00) | 2(5.00) | 0(0.00) | 3(7.50) | 0(0.00) | 4(10.00) | 3(7.50) | 5(12.50) | 3(7.50) | 4(10.00) | 2(5.00) | 0(0.00) | 1(2.50) | 1(2.50) | 3(7.50) | 1(2.50) | 1(2.50) | 1(2.50) | 0(0.00) |
| Trandolapril | C09AA | 1(7.14) | 2(14.29) | 7(50.00) | 1(7.14) | 0(0.00) | 0(0.00) | 0(0.00) | 1(7.14) | 0(0.00) | 0(0.00) | 0(0.00) | 0(0.00) | 1(7.14) | 0(0.00) | 0(0.00) | 0(0.00) | 0(0.00) | 0(0.00) | 1(7.14) | 0(0.00) | 0(0.00) | 0(0.00) |
| Candesartan | C09CA | 0(0.00) | 1(2.44) | 1(2.44) | 0(0.00) | 0(0.00) | 1(2.44) | 0(0.00) | 0(0.00) | 0(0.00) | 1(2.44) | 0(0.00) | 0(0.00) | 1(2.44) | 1(2.44) | 0(0.00) | 8(19.51) | 9(21.95) | 15(36.59) | 0(0.00) | 1(2.44) | 2(4.88) | 0(0.00) |
| Losartan | C09CA | 0(0.00) | 7(4.43) | 3(1.90) | 9(5.70) | 8(5.06) | 8(5.06) | 3(1.90) | 3(1.90) | 2(1.27) | 5(3.16) | 2(1.27) | 10(6.33) | 8(5.06) | 17(10.76) | 3(1.90) | 5(3.16) | 17(10.76) | 28(17.72) | 13(8.23) | 2(1.27) | 5(3.16) | 0(0.00) |
| Olmesartan | C09CA | 0(0.00) | 1(0.31) | 1(0.31) | 0(0.00) | 0(0.00) | 2(0.63) | 3(0.94) | 6(1.88) | 2(0.63) | 3(0.94) | 6(1.88) | 2(0.63) | 5(1.57) | 7(2.19) | 4(1.25) | 160(50.16) | 111(34.80) | 2(0.63) | 2(0.63) | 2(0.63) | 0(0.00) | 0(0.00) |
| Pravastatin | C10AA | 0(0.00) | 1(1.96) | 0(0.00) | 0(0.00) | 1(1.96) | 1(1.96) | 1(1.96) | 2(3.92) | 1(1.96) | 1(1.96) | 1(1.96) | 5(9.80) | 6(11.76) | 4(7.84) | 1(1.96) | 1(1.96) | 10(19.61) | 2(3.92) | 9(17.65) | 1(1.96) | 3(5.88) | 0(0.00) |
| Simvastatin | C10AA | 0(0.00) | 9(1.91) | 38(8.05) | 40(8.47) | 29(6.14) | 37(7.84) | 26(5.51) | 28(5.93) | 12(2.54) | 18(3.81) | 23(4.87) | 29(6.14) | 25(5.30) | 21(4.45) | 15(3.18) | 23(4.87) | 23(4.87) | 23(4.87) | 15(3.18) | 33(6.99) | 3(0.64) | 2(0.42) |
| Fenofibrate | C10AB | 1(0.63) | 8(5.06) | 9(5.70) | 9(5.70) | 4(2.53) | 15(9.49) | 10(6.33) | 6(3.80) | 9(5.70) | 8(5.06) | 1(0.63) | 1(0.63) | 2(1.27) | 1(0.63) | 3(1.90) | 16(10.13) | 1(0.63) | 18(11.39) | 17(10.76) | 13(8.23) | 6(3.80) | 0(0.00) |
| Fenofibric acid | C10AB | 0(0.00) | 0(0.00) | 0(0.00) | 0(0.00) | 0(0.00) | 0(0.00) | 12(23.53) | 24(47.06) | 4(7.84) | 8(15.69) | 1(1.96) | 0(0.00) | 1(1.96) | 0(0.00) | 0(0.00) | 0(0.00) | 1(1.96) | 0(0.00) | 0(0.00) | 0(0.00) | 0(0.00) | 0(0.00) |
| Bempedoic acid | C10AX | 0(0.00) | 0(0.00) | 0(0.00) | 0(0.00) | 0(0.00) | 0(0.00) | 0(0.00) | 0(0.00) | 0(0.00) | 0(0.00) | 0(0.00) | 0(0.00) | 0(0.00) | 0(0.00) | 0(0.00) | 0(0.00) | 0(0.00) | 1(8.33) | 4(33.33) | 6(50.00) | 1(8.33) | 0(0.00) |
| Ezetimibe | C10AX | 0(0.00) | 27(6.98) | 59(15.25) | 61(15.76) | 42(10.85) | 30(7.75) | 16(4.13) | 21(5.43) | 11(2.84) | 14(3.62) | 13(3.36) | 8(2.07) | 7(1.81) | 8(2.07) | 5(1.29) | 9(2.33) | 12(3.10) | 7(1.81) | 18(4.65) | 15(3.88) | 2(0.52) | 2(0.52) |
| Terbinafine | D01AE | 1(1.14) | 5(5.68) | 8(9.09) | 4(4.55) | 4(4.55) | 2(2.27) | 14(15.91) | 0(0.00) | 7(7.95) | 3(3.41) | 3(3.41) | 7(7.95) | 2(2.27) | 1(1.14) | 1(1.14) | 1(1.14) | 6(6.82) | 3(3.41) | 9(10.23) | 6(6.82) | 1(1.14) | 0(0.00) |
| Ethanol | D08AX | 0(0.00) | 0(0.00) | 0(0.00) | 0(0.00) | 0(0.00) | 0(0.00) | 1(1.75) | 0(0.00) | 0(0.00) | 0(0.00) | 1(1.75) | 10(17.54) | 9(15.79) | 5(8.77) | 3(5.26) | 8(14.04) | 7(12.28) | 6(10.53) | 2(3.51) | 5(8.77) | 0(0.00) | 0(0.00) |
| Drospirenone | G03AC | 0(0.00) | 5(0.85) | 4(0.68) | 4(0.68) | 3(0.51) | 8(1.36) | 12(2.04) | 28(4.75) | 231(39.22) | 158(26.83) | 54(9.17) | 36(6.11) | 6(1.02) | 8(1.36) | 2(0.34) | 3(0.51) | 7(1.19) | 4(0.68) | 3(0.51) | 6(1.02) | 7(1.19) | 0(0.00) |
| Norethisterone | G03AC | 0(0.00) | 0(0.00) | 1(0.44) | 0(0.00) | 1(0.44) | 0(0.00) | 1(0.44) | 9(3.98) | 57(25.22) | 32(14.16) | 41(18.14) | 31(13.72) | 22(9.73) | 11(4.87) | 7(3.10) | 7(3.10) | 0(0.00) | 0(0.00) | 3(1.33) | 2(0.88) | 1(0.44) | 0(0.00) |
| Equilin | G03CA | 0(0.00) | 0(0.00) | 0(0.00) | 0(0.00) | 0(0.00) | 0(0.00) | 0(0.00) | 0(0.00) | 0(0.00) | 0(0.00) | 0(0.00) | 0(0.00) | 3(100.00) | 0(0.00) | 0(0.00) | 0(0.00) | 0(0.00) | 0(0.00) | 0(0.00) | 0(0.00) | 0(0.00) | 0(0.00) |
| Thiamazole | H03BB | 0(0.00) | 0(0.00) | 0(0.00) | 0(0.00) | 0(0.00) | 0(0.00) | 3(33.33) | 0(0.00) | 0(0.00) | 2(22.22) | 0(0.00) | 0(0.00) | 2(22.22) | 1(11.11) | 1(11.11) | 0(0.00) | 0(0.00) | 0(0.00) | 0(0.00) | 0(0.00) | 0(0.00) | 0(0.00) |
| Doxercalciferol | H05BX | 0(0.00) | 0(0.00) | 2(22.22) | 0(0.00) | 0(0.00) | 0(0.00) | 0(0.00) | 0(0.00) | 0(0.00) | 7(77.78) | 0(0.00) | 0(0.00) | 0(0.00) | 0(0.00) | 0(0.00) | 0(0.00) | 0(0.00) | 0(0.00) | 0(0.00) | 0(0.00) | 0(0.00) | 0(0.00) |
| Doxycycline | J01AA | 0(0.00) | 2(0.70) | 2(0.70) | 5(1.75) | 3(1.05) | 1(0.35) | 3(1.05) | 16(5.61) | 9(3.16) | 0(0.00) | 20(7.02) | 19(6.67) | 30(10.53) | 41(14.39) | 10(3.51) | 3(1.05) | 23(8.07) | 33(11.58) | 34(11.93) | 10(3.51) | 20(7.02) | 1(0.35) |
| Tigecycline | J01AA | 0(0.00) | 0(0.00) | 10(5.59) | 4(2.23) | 11(6.15) | 12(6.70) | 11(6.15) | 32(17.88) | 13(7.26) | 12(6.70) | 22(12.29) | 2(1.12) | 4(2.23) | 4(2.23) | 5(2.79) | 3(1.68) | 9(5.03) | 8(4.47) | 11(6.15) | 4(2.23) | 2(1.12) | 0(0.00) |
| Cefpodoxime | J01DD | 0(0.00) | 0(0.00) | 1(11.11) | 1(11.11) | 0(0.00) | 0(0.00) | 0(0.00) | 0(0.00) | 3(33.33) | 3(33.33) | 0(0.00) | 1(11.11) | 0(0.00) | 0(0.00) | 0(0.00) | 0(0.00) | 0(0.00) | 0(0.00) | 0(0.00) | 0(0.00) | 0(0.00) | 0(0.00) |
| Metronidazole | J01XD | 0(0.00) | 4(2.22) | 6(3.33) | 10(5.56) | 8(4.44) | 4(2.22) | 3(1.67) | 3(1.67) | 4(2.22) | 7(3.89) | 3(1.67) | 4(2.22) | 2(1.11) | 6(3.33) | 29(16.11) | 17(9.44) | 16(8.89) | 29(16.11) | 16(8.89) | 9(5.00) | 0(0.00) | 0(0.00) |
| Tinidazole | J01XD | 0(0.00) | 0(0.00) | 0(0.00) | 0(0.00) | 0(0.00) | 0(0.00) | 0(0.00) | 0(0.00) | 2(50.00) | 0(0.00) | 0(0.00) | 0(0.00) | 2(50.00) | 0(0.00) | 0(0.00) | 0(0.00) | 0(0.00) | 0(0.00) | 0(0.00) | 0(0.00) | 0(0.00) | 0(0.00) |
| Linezolid | J01XX | 0(0.00) | 4(3.13) | 6(4.69) | 8(6.25) | 5(3.91) | 3(2.34) | 1(0.78) | 1(0.78) | 4(3.13) | 3(2.34) | 7(5.47) | 4(3.13) | 2(1.56) | 5(3.91) | 5(3.91) | 12(9.38) | 10(7.81) | 22(17.19) | 2(1.56) | 23(17.97) | 0(0.00) | 1(0.78) |
| Rifampicin | J04AB | 2(3.39) | 2(3.39) | 3(5.08) | 3(5.08) | 1(1.69) | 2(3.39) | 2(3.39) | 6(10.17) | 1(1.69) | 4(6.78) | 1(1.69) | 2(3.39) | 10(16.95) | 6(10.17) | 1(1.69) | 1(1.69) | 5(8.47) | 3(5.08) | 4(6.78) | 0(0.00) | 0(0.00) | 0(0.00) |
| Isoniazid | J04AC | 1(1.43) | 0(0.00) | 4(5.71) | 3(4.29) | 2(2.86) | 5(7.14) | 0(0.00) | 0(0.00) | 2(2.86) | 3(4.29) | 2(2.86) | 4(5.71) | 3(4.29) | 4(5.71) | 4(5.71) | 9(12.86) | 13(18.57) | 2(2.86) | 3(4.29) | 6(8.57) | 0(0.00) | 0(0.00) |
| Bedaquiline | J04AK | 0(0.00) | 0(0.00) | 0(0.00) | 0(0.00) | 0(0.00) | 0(0.00) | 0(0.00) | 0(0.00) | 0(0.00) | 0(0.00) | 0(0.00) | 0(0.00) | 0(0.00) | 3(13.04) | 2(8.70) | 3(13.04) | 3(13.04) | 2(8.70) | 2(8.70) | 7(30.43) | 1(4.35) | 0(0.00) |
| Pyrazinamide | J04AK | 1(9.09) | 0(0.00) | 1(9.09) | 2(18.18) | 1(9.09) | 2(18.18) | 0(0.00) | 0(0.00) | 0(0.00) | 0(0.00) | 2(18.18) | 0(0.00) | 0(0.00) | 0(0.00) | 0(0.00) | 0(0.00) | 1(9.09) | 0(0.00) | 0(0.00) | 1(9.09) | 0(0.00) | 0(0.00) |
| Foscarnet | J05AD | 0(0.00) | 1(9.09) | 0(0.00) | 0(0.00) | 0(0.00) | 0(0.00) | 0(0.00) | 0(0.00) | 0(0.00) | 0(0.00) | 0(0.00) | 0(0.00) | 0(0.00) | 2(18.18) | 6(54.55) | 0(0.00) | 1(9.09) | 0(0.00) | 0(0.00) | 0(0.00) | 1(9.09) | 0(0.00) |
| Atazanavir | J05AE | 0(0.00) | 7(12.28) | 11(19.30) | 5(8.77) | 4(7.02) | 4(7.02) | 2(3.51) | 4(7.02) | 6(10.53) | 5(8.77) | 0(0.00) | 1(1.75) | 0(0.00) | 2(3.51) | 1(1.75) | 0(0.00) | 0(0.00) | 4(7.02) | 1(1.75) | 0(0.00) | 0(0.00) | 0(0.00) |
| Fosamprenavir | J05AE | 0(0.00) | 0(0.00) | 2(20.00) | 2(20.00) | 1(10.00) | 1(10.00) | 1(10.00) | 0(0.00) | 0(0.00) | 0(0.00) | 1(10.00) | 0(0.00) | 0(0.00) | 1(10.00) | 0(0.00) | 0(0.00) | 1(10.00) | 0(0.00) | 0(0.00) | 0(0.00) | 0(0.00) | 0(0.00) |
| Indinavir | J05AE | 0(0.00) | 3(37.50) | 2(25.00) | 0(0.00) | 2(25.00) | 1(12.50) | 0(0.00) | 0(0.00) | 0(0.00) | 0(0.00) | 0(0.00) | 0(0.00) | 0(0.00) | 0(0.00) | 0(0.00) | 0(0.00) | 0(0.00) | 0(0.00) | 0(0.00) | 0(0.00) | 0(0.00) | 0(0.00) |
| Abacavir | J05AF | 0(0.00) | 5(4.95) | 6(5.94) | 4(3.96) | 4(3.96) | 5(4.95) | 3(2.97) | 6(5.94) | 2(1.98) | 3(2.97) | 1(0.99) | 6(5.94) | 11(10.89) | 6(5.94) | 8(7.92) | 7(6.93) | 5(4.95) | 1(0.99) | 4(3.96) | 10(9.90) | 3(2.97) | 1(0.99) |
| Didanosine | J05AF | 0(0.00) | 10(19.61) | 10(19.61) | 18(35.29) | 2(3.92) | 1(1.96) | 1(1.96) | 1(1.96) | 1(1.96) | 0(0.00) | 1(1.96) | 0(0.00) | 3(5.88) | 1(1.96) | 0(0.00) | 0(0.00) | 0(0.00) | 0(0.00) | 1(1.96) | 1(1.96) | 0(0.00) | 0(0.00) |
| Stavudine | J05AF | 2(3.92) | 9(17.65) | 6(11.76) | 13(25.49) | 3(5.88) | 3(5.88) | 1(1.96) | 2(3.92) | 0(0.00) | 4(7.84) | 0(0.00) | 1(1.96) | 1(1.96) | 0(0.00) | 3(5.88) | 2(3.92) | 0(0.00) | 1(1.96) | 0(0.00) | 0(0.00) | 0(0.00) | 0(0.00) |
| Nevirapine | J05AG | 0(0.00) | 7(10.61) | 10(15.15) | 10(15.15) | 6(9.09) | 1(1.52) | 2(3.03) | 3(4.55) | 11(16.67) | 4(6.06) | 2(3.03) | 0(0.00) | 3(4.55) | 1(1.52) | 2(3.03) | 0(0.00) | 1(1.52) | 1(1.52) | 2(3.03) | 0(0.00) | 0(0.00) | 0(0.00) |
| Raltegravir | J05AJ | 0(0.00) | 0(0.00) | 0(0.00) | 0(0.00) | 0(0.00) | 6(9.23) | 9(13.85) | 8(12.31) | 3(4.62) | 1(1.54) | 10(15.38) | 6(9.23) | 3(4.62) | 5(7.69) | 5(7.69) | 3(4.62) | 1(1.54) | 4(6.15) | 1(1.54) | 0(0.00) | 0(0.00) | 0(0.00) |
| Enfuvirtide | J05AX | 0(0.00) | 6(22.22) | 6(22.22) | 1(3.70) | 3(11.11) | 2(7.41) | 9(33.33) | 0(0.00) | 0(0.00) | 0(0.00) | 0(0.00) | 0(0.00) | 0(0.00) | 0(0.00) | 0(0.00) | 0(0.00) | 0(0.00) | 0(0.00) | 0(0.00) | 0(0.00) | 0(0.00) | 0(0.00) |
| Clofarabine | L01BB | 0(0.00) | 0(0.00) | 4(9.76) | 2(4.88) | 4(9.76) | 6(14.63) | 1(2.44) | 7(17.07) | 2(4.88) | 4(9.76) | 1(2.44) | 1(2.44) | 3(7.32) | 2(4.88) | 1(2.44) | 2(4.88) | 0(0.00) | 1(2.44) | 0(0.00) | 0(0.00) | 0(0.00) | 0(0.00) |
| Mercaptopurine | L01BB | 0(0.00) | 3(2.34) | 0(0.00) | 3(2.34) | 4(3.13) | 2(1.56) | 4(3.13) | 7(5.47) | 6(4.69) | 5(3.91) | 15(11.72) | 5(3.91) | 9(7.03) | 9(7.03) | 8(6.25) | 7(5.47) | 19(14.84) | 6(4.69) | 10(7.81) | 5(3.91) | 1(0.78) | 0(0.00) |
| Tioguanine | L01BB | 0(0.00) | 0(0.00) | 0(0.00) | 0(0.00) | 0(0.00) | 0(0.00) | 0(0.00) | 1(16.67) | 0(0.00) | 0(0.00) | 0(0.00) | 0(0.00) | 1(16.67) | 0(0.00) | 0(0.00) | 1(16.67) | 2(33.33) | 1(16.67) | 0(0.00) | 0(0.00) | 0(0.00) | 0(0.00) |
| Vincristine | L01CA | 0(0.00) | 0(0.00) | 0(0.00) | 0(0.00) | 2(2.82) | 1(1.41) | 0(0.00) | 2(2.82) | 2(2.82) | 0(0.00) | 3(4.23) | 1(1.41) | 2(2.82) | 3(4.23) | 2(2.82) | 17(23.94) | 8(11.27) | 6(8.45) | 8(11.27) | 8(11.27) | 6(8.45) | 0(0.00) |
| Daunorubicin | L01DB | 0(0.00) | 1(3.70) | 2(7.41) | 1(3.70) | 0(0.00) | 0(0.00) | 3(11.11) | 2(7.41) | 0(0.00) | 2(7.41) | 1(3.70) | 1(3.70) | 2(7.41) | 2(7.41) | 1(3.70) | 0(0.00) | 0(0.00) | 1(3.70) | 6(22.22) | 2(7.41) | 0(0.00) | 0(0.00) |
| Lenvatinib | L01EX | 0(0.00) | 0(0.00) | 0(0.00) | 0(0.00) | 0(0.00) | 0(0.00) | 0(0.00) | 0(0.00) | 0(0.00) | 0(0.00) | 0(0.00) | 0(0.00) | 1(1.43) | 0(0.00) | 3(4.29) | 1(1.43) | 10(14.29) | 15(21.43) | 12(17.14) | 19(27.14) | 9(12.86) | 0(0.00) |
| Brentuximab vedotin | L01FX | 0(0.00) | 0(0.00) | 0(0.00) | 0(0.00) | 0(0.00) | 0(0.00) | 0(0.00) | 0(0.00) | 2(2.25) | 1(1.12) | 5(5.62) | 21(23.60) | 4(4.49) | 11(12.36) | 4(4.49) | 5(5.62) | 6(6.74) | 6(6.74) | 14(15.73) | 5(5.62) | 3(3.37) | 2(2.25) |
| Nilotinib | L01XE | 0(0.00) | 0(0.00) | 0(0.00) | 0(0.00) | 0(0.00) | 5(1.12) | 7(1.56) | 15(3.35) | 12(2.68) | 37(8.26) | 42(9.38) | 41(9.15) | 32(7.14) | 49(10.94) | 37(8.26) | 41(9.15) | 39(8.71) | 29(6.47) | 40(8.93) | 17(3.79) | 5(1.12) | 0(0.00) |
| Ponatinib | L01XE | 0(0.00) | 0(0.00) | 0(0.00) | 0(0.00) | 0(0.00) | 0(0.00) | 0(0.00) | 0(0.00) | 0(0.00) | 0(0.00) | 28(15.38) | 20(10.99) | 2(1.10) | 19(10.44) | 17(9.34) | 8(4.40) | 12(6.59) | 20(10.99) | 23(12.64) | 29(15.93) | 4(2.20) | 0(0.00) |
| Arsenic trioxide | L01XX | 0(0.00) | 2(14.29) | 2(14.29) | 1(7.14) | 0(0.00) | 1(7.14) | 0(0.00) | 0(0.00) | 0(0.00) | 0(0.00) | 1(7.14) | 0(0.00) | 0(0.00) | 0(0.00) | 0(0.00) | 0(0.00) | 0(0.00) | 2(14.29) | 5(35.71) | 0(0.00) | 0(0.00) | 0(0.00) |
| Asparaginase | L01XX | 0(0.00) | 2(1.23) | 1(0.61) | 5(3.07) | 0(0.00) | 3(1.84) | 5(3.07) | 20(12.27) | 5(3.07) | 16(9.82) | 23(14.11) | 9(5.52) | 2(1.23) | 4(2.45) | 4(2.45) | 6(3.68) | 13(7.98) | 18(11.04) | 4(2.45) | 17(10.43) | 3(1.84) | 3(1.84) |
| Pegaspargase | L01XX | 1(0.37) | 2(0.75) | 4(1.50) | 1(0.37) | 0(0.00) | 4(1.50) | 12(4.49) | 10(3.75) | 5(1.87) | 11(4.12) | 3(1.12) | 4(1.50) | 5(1.87) | 15(5.62) | 14(5.24) | 36(13.48) | 17(6.37) | 18(6.74) | 23(8.61) | 67(25.09) | 15(5.62) | 0(0.00) |
| Basiliximab | L04AC | 0(0.00) | 1(4.76) | 0(0.00) | 0(0.00) | 0(0.00) | 2(9.52) | 0(0.00) | 1(4.76) | 0(0.00) | 1(4.76) | 0(0.00) | 0(0.00) | 1(4.76) | 2(9.52) | 3(14.29) | 3(14.29) | 3(14.29) | 2(9.52) | 0(0.00) | 2(9.52) | 0(0.00) | 0(0.00) |
| Azathioprine | L04AX | 0(0.00) | 10(4.20) | 2(0.84) | 8(3.36) | 5(2.10) | 3(1.26) | 16(6.72) | 3(1.26) | 11(4.62) | 6(2.52) | 14(5.88) | 11(4.62) | 11(4.62) | 12(5.04) | 10(4.20) | 35(14.71) | 17(7.14) | 26(10.92) | 12(5.04) | 22(9.24) | 2(0.84) | 2(0.84) |
| Flurbiprofen | M01AE | 0(0.00) | 0(0.00) | 0(0.00) | 0(0.00) | 1(20.00) | 0(0.00) | 0(0.00) | 0(0.00) | 2(40.00) | 0(0.00) | 0(0.00) | 0(0.00) | 0(0.00) | 0(0.00) | 0(0.00) | 0(0.00) | 2(40.00) | 0(0.00) | 0(0.00) | 0(0.00) | 0(0.00) | 0(0.00) |
| Ketoprofen | M01AE | 0(0.00) | 0(0.00) | 0(0.00) | 0(0.00) | 0(0.00) | 0(0.00) | 0(0.00) | 0(0.00) | 0(0.00) | 1(5.88) | 0(0.00) | 1(5.88) | 0(0.00) | 0(0.00) | 0(0.00) | 8(47.06) | 3(17.65) | 1(5.88) | 2(11.76) | 1(5.88) | 0(0.00) | 0(0.00) |
| Propofol | N01AX | 0(0.00) | 3(3.26) | 8(8.70) | 4(4.35) | 1(1.09) | 4(4.35) | 4(4.35) | 5(5.43) | 1(1.09) | 5(5.43) | 4(4.35) | 5(5.43) | 2(2.17) | 0(0.00) | 9(9.78) | 8(8.70) | 6(6.52) | 2(2.17) | 6(6.52) | 10(10.87) | 5(5.43) | 0(0.00) |
| Codeine | N02AA | 0(0.00) | 4(5.19) | 5(6.49) | 5(6.49) | 2(2.60) | 3(3.90) | 0(0.00) | 5(6.49) | 4(5.19) | 11(14.29) | 3(3.90) | 3(3.90) | 10(12.99) | 3(3.90) | 1(1.30) | 5(6.49) | 5(6.49) | 3(3.90) | 3(3.90) | 2(2.60) | 0(0.00) | 0(0.00) |
| Eslicarbazepine | N03AF | 0(0.00) | 0(0.00) | 0(0.00) | 0(0.00) | 0(0.00) | 0(0.00) | 0(0.00) | 0(0.00) | 0(0.00) | 0(0.00) | 0(0.00) | 0(0.00) | 2(25.00) | 0(0.00) | 0(0.00) | 0(0.00) | 0(0.00) | 0(0.00) | 2(25.00) | 3(37.50) | 1(12.50) | 0(0.00) |
| Rufinamide | N03AF | 0(0.00) | 0(0.00) | 0(0.00) | 0(0.00) | 0(0.00) | 0(0.00) | 1(16.67) | 2(33.33) | 0(0.00) | 0(0.00) | 0(0.00) | 1(16.67) | 0(0.00) | 0(0.00) | 1(16.67) | 1(16.67) | 0(0.00) | 0(0.00) | 0(0.00) | 0(0.00) | 0(0.00) | 0(0.00) |
| Valproic acid | N03AG | 10(2.27) | 39(8.86) | 36(8.18) | 24(5.45) | 34(7.73) | 27(6.14) | 30(6.82) | 19(4.32) | 13(2.95) | 16(3.64) | 27(6.14) | 20(4.55) | 15(3.41) | 14(3.18) | 15(3.41) | 16(3.64) | 18(4.09) | 23(5.23) | 16(3.64) | 27(6.14) | 0(0.00) | 1(0.23) |
| Fluphenazine | N05AB | 0(0.00) | 0(0.00) | 0(0.00) | 0(0.00) | 3(42.86) | 0(0.00) | 0(0.00) | 0(0.00) | 0(0.00) | 0(0.00) | 0(0.00) | 0(0.00) | 0(0.00) | 0(0.00) | 0(0.00) | 2(28.57) | 2(28.57) | 0(0.00) | 0(0.00) | 0(0.00) | 0(0.00) | 0(0.00) |
| Olanzapine | N05AH | 0(0.00) | 45(3.08) | 125(8.57) | 308(21.11) | 529(36.26) | 13(0.89) | 26(1.78) | 15(1.03) | 67(4.59) | 10(0.69) | 3(0.21) | 5(0.34) | 186(12.75) | 14(0.96) | 7(0.48) | 12(0.82) | 16(1.10) | 10(0.69) | 47(3.22) | 18(1.23) | 3(0.21) | 0(0.00) |
| Quetiapine | N05AH | 0(0.00) | 8(0.23) | 10(0.29) | 32(0.93) | 722(20.87) | 72(2.08) | 1348(38.97) | 646(18.68) | 174(5.03) | 60(1.73) | 141(4.08) | 29(0.84) | 36(1.04) | 43(1.24) | 9(0.26) | 40(1.16) | 28(0.81) | 17(0.49) | 24(0.69) | 16(0.46) | 4(0.12) | 0(0.00) |
| Meprobamate | N05BC | 0(0.00) | 0(0.00) | 0(0.00) | 0(0.00) | 0(0.00) | 0(0.00) | 0(0.00) | 0(0.00) | 3(100.00) | 0(0.00) | 0(0.00) | 0(0.00) | 0(0.00) | 0(0.00) | 0(0.00) | 0(0.00) | 0(0.00) | 0(0.00) | 0(0.00) | 0(0.00) | 0(0.00) | 0(0.00) |
| Riluzole | N07XX | 0(0.00) | 1(1.69) | 1(1.69) | 0(0.00) | 1(1.69) | 0(0.00) | 1(1.69) | 0(0.00) | 1(1.69) | 0(0.00) | 0(0.00) | 0(0.00) | 1(1.69) | 0(0.00) | 7(11.86) | 6(10.17) | 9(15.25) | 16(27.12) | 6(10.17) | 9(15.25) | 0(0.00) | 0(0.00) |
| Miltefosine | P01CX | 0(0.00) | 0(0.00) | 0(0.00) | 0(0.00) | 0(0.00) | 0(0.00) | 0(0.00) | 0(0.00) | 0(0.00) | 0(0.00) | 0(0.00) | 0(0.00) | 0(0.00) | 1(33.33) | 0(0.00) | 0(0.00) | 0(0.00) | 0(0.00) | 2(66.67) | 0(0.00) | 0(0.00) | 0(0.00) |
| Pentamidine isethionate | P01CX | 0(0.00) | 0(0.00) | 0(0.00) | 0(0.00) | 0(0.00) | 1(16.67) | 1(16.67) | 0(0.00) | 0(0.00) | 0(0.00) | 0(0.00) | 0(0.00) | 1(16.67) | 0(0.00) | 0(0.00) | 2(33.33) | 0(0.00) | 0(0.00) | 0(0.00) | 0(0.00) | 0(0.00) | 1(16.67) |
| Calcium acetate | V03AE | 0(0.00) | 2(22.22) | 0(0.00) | 0(0.00) | 0(0.00) | 0(0.00) | 0(0.00) | 0(0.00) | 0(0.00) | 0(0.00) | 0(0.00) | 0(0.00) | 2(22.22) | 0(0.00) | 0(0.00) | 0(0.00) | 4(44.44) | 0(0.00) | 1(11.11) | 0(0.00) | 0(0.00) | 0(0.00) |
| Secretin | V04CK | 0(0.00) | 0(0.00) | 0(0.00) | 0(0.00) | 0(0.00) | 0(0.00) | 0(0.00) | 4(100.00) | 0(0.00) | 0(0.00) | 0(0.00) | 0(0.00) | 0(0.00) | 0(0.00) | 0(0.00) | 0(0.00) | 0(0.00) | 0(0.00) | 0(0.00) | 0(0.00) | 0(0.00) | 0(0.00) |
| Iodixanol | V08AB | 0(0.00) | 2(5.71) | 1(2.86) | 0(0.00) | 0(0.00) | 0(0.00) | 1(2.86) | 3(8.57) | 0(0.00) | 0(0.00) | 0(0.00) | 1(2.86) | 1(2.86) | 0(0.00) | 1(2.86) | 0(0.00) | 0(0.00) | 0(0.00) | 0(0.00) | 0(0.00) | 25(71.43) | 0(0.00) |
| Iothalamic acid | V09CX | 0(0.00) | 0(0.00) | 0(0.00) | 0(0.00) | 0(0.00) | 0(0.00) | 0(0.00) | 0(0.00) | 7(100.00) | 0(0.00) | 0(0.00) | 0(0.00) | 0(0.00) | 0(0.00) | 0(0.00) | 0(0.00) | 0(0.00) | 0(0.00) | 0(0.00) | 0(0.00) | 0(0.00) | 0(0.00) |

Abbreviations: ATC, ;Anatomical Therapeutic.

Supplemental table 5. Median and mean of interval days from drug initiation to onset of pancreatitis event of 94 drugs with time information。

| Drugs | ATC code | Median of interval days | Mean of interval days | Reports (n) |
| --- | --- | --- | --- | --- |
| Eluxadoline | A07DA | 1 | 177.82 | 95 |
| Olsalazine | A07EC | 7 | 527.00 | 3 |
| Balsalazide | A07EC | 61 | 166.80 | 5 |
| Mesalazine | A07EC | 27 | 154.67 | 126 |
| Pancrelipase amylase | A09AA | 218.5 | 466.58 | 26 |
| Metformin | A10BA | 202 | 698.19 | 367 |
| Glimepiride | A10BB | 407.5 | 989.28 | 32 |
| Alogliptin | A10BH | 93 | 451.84 | 64 |
| Saxagliptin | A10BH | 128 | 390.31 | 113 |
| Linagliptin | A10BH | 86.5 | 285.80 | 118 |
| Sitagliptin | A10BH | 233 | 523.34 | 678 |
| Albiglutide | A10BJ | 69 | 125.67 | 6 |
| Lixisenatide | A10BJ | 119 | 171.17 | 6 |
| Semaglutide | A10BJ | 63 | 322.36 | 159 |
| Dulaglutide | A10BJ | 42 | 184.02 | 252 |
| Liraglutide | A10BJ | 85 | 300.15 | 1032 |
| Exenatide | A10BJ | 285.5 | 664.46 | 1390 |
| Dapagliflozin | A10BK | 55 | 268.29 | 59 |
| Canagliflozin | A10BK | 79 | 312.48 | 79 |
| Empagliflozin | A10BK | 88 | 345.03 | 80 |
| Repaglinide | A10BX | 13 | 605.00 | 3 |
| Levocarnitine | A16AA | 1.5 | 1.50 | 2 |
| Carglumic acid | A16AA | 711 | 647.86 | 7 |
| Metreleptin | A16AA | 976 | 856.09 | 23 |
| Givosiran | A16AX | 253 | 252.80 | 5 |
| Teduglutide | A16AX | 350 | 677.65 | 43 |
| Hydrochlorothiazide | C03AA | 359 | 745.17 | 321 |
| Metolazone | C03BA | 6 | 111.56 | 9 |
| Trandolapril | C09AA | 1076 | 1991.71 | 7 |
| Perindopril | C09AA | 102 | 333.47 | 19 |
| Enalapril | C09AA | 295 | 1084.93 | 41 |
| Lisinopril | C09AA | 270 | 990.82 | 119 |
| Candesartan | C09CA | 388.5 | 874.70 | 20 |
| Losartan | C09CA | 242 | 761.46 | 68 |
| Olmesartan | C09CA | 233.5 | 518.77 | 140 |
| Pravastatin | C10AA | 378 | 375.44 | 16 |
| Simvastatin | C10AA | 434 | 877.81 | 234 |
| Fenofibric acid | C10AB | 51 | 764.39 | 23 |
| Fenofibrate | C10AB | 270 | 645.90 | 52 |
| Bempedoic acid | C10AX | 60 | 60.00 | 6 |
| Ezetimibe | C10AX | 107.5 | 450.41 | 164 |
| Terbinafine | D01AE | 23 | 395.71 | 52 |
| Ethanol | D08AX | 75 | 293.46 | 13 |
| Norethisterone | G03AC | 125 | 492.39 | 62 |
| Drospirenone | G03AC | 488 | 911.59 | 381 |
| Equilin | G03CA | 426 | 3103.67 | 3 |
| Thiamazole | H03BB | 56 | 56.00 | 2 |
| Doxercalciferol | H05BX | 240 | 198.00 | 5 |
| Doxycycline | J01AA | 10 | 65.55 | 55 |
| Tigecycline | J01AA | 8 | 20.94 | 88 |
| Cefpodoxime | J01DD | 2 | 7.67 | 9 |
| Tinidazole | J01XD | 5 | 5.00 | 2 |
| Metronidazole | J01XD | 10 | 172.43 | 56 |
| Linezolid | J01XX | 10 | 133.38 | 64 |
| Rifampicin | J04AB | 17.5 | 190.20 | 30 |
| Isoniazid | J04AC | 31 | 405.55 | 11 |
| Pyrazinamide | J04AK | 21 | 261.00 | 5 |
| Bedaquiline | J04AK | 128 | 175.57 | 14 |
| Foscarnet | J05AD | 56 | 56.00 | 2 |
| Fosamprenavir | J05AE | 248 | 1553.43 | 7 |
| Atazanavir | J05AE | 977 | 1006.69 | 26 |
| Didanosine | J05AF | 567.5 | 1116.31 | 16 |
| Stavudine | J05AF | 457 | 909.67 | 21 |
| Abacavir | J05AF | 298 | 760.86 | 43 |
| Nevirapine | J05AG | 205 | 619.00 | 23 |
| Raltegravir | J05AJ | 297 | 726.68 | 41 |
| Enfuvirtide | J05AX | 518 | 577.90 | 21 |
| Tioguanine | L01BB | 55 | 62.00 | 3 |
| Clofarabine | L01BB | 8 | 44.06 | 35 |
| Mercaptopurine | L01BB | 29.5 | 144.26 | 38 |
| Vincristine | L01CA | 30 | 259.81 | 43 |
| Daunorubicin | L01DB | 23 | 26.50 | 4 |
| Lenvatinib | L01EX | 125 | 256.54 | 65 |
| Brentuximab vedotin | L01FX | 13 | 25.49 | 43 |
| Ponatinib | L01XE | 32 | 274.23 | 77 |
| Nilotinib | L01XE | 13.5 | 272.20 | 148 |
| Arsenic trioxide | L01XX | 17 | 22.40 | 5 |
| Asparaginase | L01XX | 15 | 554.59 | 29 |
| Pegaspargase | L01XX | 23 | 75.03 | 135 |
| Basiliximab | L04AC | 2 | 283.00 | 13 |
| Azathioprine | L04AX | 29.5 | 176.77 | 90 |
| Flurbiprofen | M01AE | 1 | 61.33 | 3 |
| Ketoprofen | M01AE | 0.5 | 9.80 | 10 |
| Propofol | N01AX | 3 | 139.45 | 44 |
| Codeine | N02AA | 2.5 | 209.32 | 22 |
| Eslicarbazepine | N03AF | 143 | 166.33 | 3 |
| Rufinamide | N03AF | 169 | 155.33 | 3 |
| Valproic acid | N03AG | 437 | 1329.26 | 163 |
| Olanzapine | N05AH | 514 | 881.08 | 646 |
| Quetiapine | N05AH | 823 | 1342.82 | 1308 |
| Riluzole | N07XX | 100 | 716.45 | 20 |
| Pentamidine isethionate | P01CX | 5 | 7.67 | 3 |
| Secretin | V04CK | 0 | 0.00 | 4 |
| Iodixanol | V08AB | 0 | 0.03 | 32 |

Abbreviations: ATC, ;Anatomical Therapeutic.

Supplemental table 6. Outcomes of patients with drug-associated pancreatitis of 101 drugs.

| Drugs | ATC code | Outcome (n, %) | | | | | | | Reports (N) | Fatality rate (%)* |
| --- | --- | --- | --- | --- | --- | --- | --- | --- | --- | --- |
|  |  | Congenital Anomaly | Death | Disability | Hospitalization - Initial or Prolonged | Life-Threatening | Other Serious (Important Medical Event) | Required Intervention to Prevent Permanent Impairment/Damage |  | (95% two-sided CI) |
| Calcium carbonate | A02AC | 0(0.00) | 0(0.00) | 0(0.00) | 21(70.00) | 5(16.67) | 19(63.33) | 0(0.00) | 30 | 0.00 |
| Eluxadoline | A07DA | 0(0.00) | 5(1.43) | 8(2.29) | 212(60.57) | 6(1.71) | 172(49.14) | 0(0.00) | 350 | 1.43 |
| Balsalazide | A07EC | 0(0.00) | 0(0.00) | 1(11.11) | 7(77.78) | 0(0.00) | 2(22.22) | 0(0.00) | 9 | 0.00 |
| Mesalazine | A07EC | 0(0.00) | 0(0.00) | 3(0.94) | 194(61.01) | 7(2.20) | 207(65.09) | 2(0.63) | 318 | 0.00 |
| Olsalazine | A07EC | 0(0.00) | 0(0.00) | 0(0.00) | 1(33.33) | 0(0.00) | 3(100.00) | 1(33.33) | 3 | 0.00 |
| Pancrelipase amylase | A09AA | 2(0.53) | 8(2.13) | 0(0.00) | 254(67.73) | 5(1.33) | 231(61.60) | 0(0.00) | 375 | 2.13(0.67,3.60) |
| Metformin | A10BA | 0(0.00) | 113(6.69) | 69(4.09) | 1150(68.09) | 202(11.96) | 1013(59.98) | 11(0.65) | 1689 | 6.69(5.50,7.88) |
| Glimepiride | A10BB | 0(0.00) | 1(1.59) | 2(3.17) | 43(68.25) | 4(6.35) | 31(49.21) | 0(0.00) | 63 | 1.59 |
| Alogliptin | A10BH | 0(0.00) | 1(0.79) | 3(2.38) | 87(69.05) | 5(3.97) | 85(67.46) | 7(5.56) | 126 | 0.79 |
| Linagliptin | A10BH | 0(0.00) | 17(3.83) | 8(1.80) | 266(59.91) | 22(4.95) | 226(50.90) | 0(0.00) | 444 | 3.83(2.04,5.61) |
| Saxagliptin | A10BH | 0(0.00) | 8(2.47) | 4(1.23) | 182(56.17) | 25(7.72) | 159(49.07) | 1(0.31) | 324 | 2.47(0.78,4.16) |
| Sitagliptin | A10BH | 1(0.03) | 346(10.99) | 254(8.07) | 1891(60.05) | 391(12.42) | 2362(75.01) | 9(0.29) | 3149 | 10.99(9.90,12.08) |
| Albiglutide | A10BJ | 0(0.00) | 1(16.67) | 0(0.00) | 5(83.33) | 0(0.00) | 1(16.67) | 1(16.67) | 6 | 16.67 |
| Dulaglutide | A10BJ | 0(0.00) | 29(2.08) | 11(0.79) | 590(42.39) | 38(2.73) | 892(64.08) | 7(0.50) | 1392 | 2.08(1.33,2.83) |
| Exenatide | A10BJ | 0(0.00) | 150(4.74) | 72(2.28) | 1797(56.83) | 147(4.65) | 2283(72.20) | 15(0.47) | 3162 | 4.74(4.00,5.48) |
| Liraglutide | A10BJ | 0(0.00) | 46(1.63) | 22(0.78) | 1417(50.32) | 67(2.38) | 1644(58.38) | 8(0.28) | 2816 | 1.63(1.17,2.10) |
| Lixisenatide | A10BJ | 0(0.00) | 0(0.00) | 1(2.78) | 16(44.44) | 1(2.78) | 28(77.78) | 0(0.00) | 36 | 0.00 |
| Semaglutide | A10BJ | 1(0.16) | 13(2.04) | 8(1.25) | 266(41.69) | 20(3.13) | 405(63.48) | 9(1.41) | 638 | 2.04(0.94,3.13) |
| Canagliflozin | A10BK | 0(0.00) | 12(3.34) | 8(2.23) | 253(70.47) | 29(8.08) | 140(39.00) | 1(0.28) | 359 | 3.34(1.48,5.20) |
| Dapagliflozin | A10BK | 0(0.00) | 4(1.57) | 2(0.79) | 143(56.30) | 17(6.69) | 161(63.39) | 0(0.00) | 254 | 1.57 |
| Empagliflozin | A10BK | 1(0.26) | 10(2.65) | 5(1.32) | 216(57.14) | 36(9.52) | 281(74.34) | 1(0.26) | 378 | 2.65(1.03,4.26) |
| Repaglinide | A10BX | 0(0.00) | 1(9.09) | 0(0.00) | 8(72.73) | 0(0.00) | 9(81.82) | 0(0.00) | 11 | 9.09 |
| Carglumic acid | A16AA | 0(0.00) | 4(26.67) | 0(0.00) | 14(93.33) | 0(0.00) | 5(33.33) | 0(0.00) | 15 | 26.67 |
| Levocarnitine | A16AA | 0(0.00) | 1(25.00) | 0(0.00) | 3(75.00) | 0(0.00) | 3(75.00) | 0(0.00) | 4 | 25.00 |
| Metreleptin | A16AA | 0(0.00) | 1(1.92) | 0(0.00) | 48(92.31) | 0(0.00) | 16(30.77) | 0(0.00) | 52 | 1.92 |
| Givosiran | A16AX | 0(0.00) | 0(0.00) | 0(0.00) | 6(46.15) | 1(7.69) | 13(100.00) | 0(0.00) | 13 | 0.00 |
| Teduglutide | A16AX | 0(0.00) | 7(6.93) | 0(0.00) | 82(81.19) | 0(0.00) | 85(84.16) | 0(0.00) | 101 | 6.93(1.98,11.88) |
| Hydrochlorothiazide | C03AA | 0(0.00) | 48(6.45) | 8(1.08) | 627(84.27) | 60(8.06) | 410(55.11) | 19(2.55) | 744 | 6.45(4.69,8.22) |
| Metolazone | C03BA | 0(0.00) | 0(0.00) | 1(9.09) | 10(90.91) | 2(18.18) | 2(18.18) | 0(0.00) | 11 | 0.00 |
| Enalapril | C09AA | 0(0.00) | 10(14.49) | 1(1.45) | 56(81.16) | 7(10.14) | 18(26.09) | 1(1.45) | 69 | 14.49(6.19,22.80) |
| Lisinopril | C09AA | 0(0.00) | 21(6.98) | 15(4.98) | 251(83.39) | 53(17.61) | 131(43.52) | 25(8.31) | 301 | 6.98(4.10,9.85) |
| Perindopril | C09AA | 0(0.00) | 7(17.50) | 0(0.00) | 34(85.00) | 4(10.00) | 13(32.50) | 0(0.00) | 40 | 17.50(5.72,29.28) |
| Trandolapril | C09AA | 0(0.00) | 6(42.86) | 0(0.00) | 9(64.29) | 2(14.29) | 2(14.29) | 0(0.00) | 14 | 42.86(16.93,68.78) |
| Candesartan | C09CA | 0(0.00) | 2(4.88) | 0(0.00) | 37(90.24) | 12(29.27) | 6(14.63) | 0(0.00) | 41 | 4.88 |
| Losartan | C09CA | 0(0.00) | 0(0.00) | 4(2.60) | 111(72.08) | 19(12.34) | 77(50.00) | 1(0.65) | 154 | 0.00 |
| Olmesartan | C09CA | 0(0.00) | 5(1.58) | 0(0.00) | 287(90.54) | 3(0.95) | 241(76.03) | 1(0.32) | 317 | 1.58 |
| Pravastatin | C10AA | 0(0.00) | 3(5.88) | 1(1.96) | 39(76.47) | 6(11.76) | 25(49.02) | 0(0.00) | 51 | 5.88 |
| Simvastatin | C10AA | 0(0.00) | 53(11.32) | 33(7.05) | 334(71.37) | 108(23.08) | 197(42.09) | 6(1.28) | 468 | 11.32(8.45,14.20) |
| Fenofibrate | C10AB | 1(0.66) | 8(5.26) | 12(7.89) | 129(84.87) | 17(11.18) | 56(36.84) | 3(1.97) | 152 | 5.26(1.71,8.81) |
| Fenofibric acid | C10AB | 0(0.00) | 1(1.96) | 1(1.96) | 29(56.86) | 1(1.96) | 30(58.82) | 0(0.00) | 51 | 1.96 |
| Bempedoic acid | C10AX | 0(0.00) | 0(0.00) | 0(0.00) | 8(66.67) | 0(0.00) | 11(91.67) | 0(0.00) | 12 | 0.00 |
| Ezetimibe | C10AX | 0(0.00) | 15(3.94) | 20(5.25) | 253(66.40) | 64(16.80) | 204(53.54) | 7(1.84) | 381 | 3.94(1.98,5.89) |
| Terbinafine | D01AE | 0(0.00) | 1(1.14) | 6(6.82) | 76(86.36) | 9(10.23) | 50(56.82) | 0(0.00) | 88 | 1.14 |
| Ethanol | D08AX | 0(0.00) | 5(8.77) | 0(0.00) | 42(73.68) | 0(0.00) | 41(71.93) | 0(0.00) | 57 | 8.77 |
| Drospirenone | G03AC | 0(0.00) | 3(0.51) | 8(1.36) | 480(81.77) | 22(3.75) | 380(64.74) | 1(0.17) | 587 | 0.51 |
| Norethisterone | G03AC | 0(0.00) | 6(2.69) | 4(1.79) | 121(54.26) | 19(8.52) | 112(50.22) | 1(0.45) | 223 | 2.69(0.57,4.81) |
| Equilin | G03CA | 0(0.00) | 0(0.00) | 0(0.00) | 3(100.00) | 0(0.00) | 0(0.00) | 0(0.00) | 3 | 0.00 |
| Thiamazole | H03BB | 0(0.00) | 2(22.22) | 0(0.00) | 7(77.78) | 1(11.11) | 3(33.33) | 0(0.00) | 9 | 22.22 |
| Doxercalciferol | H05BX | 0(0.00) | 0(0.00) | 0(0.00) | 6(66.67) | 0(0.00) | 5(55.56) | 0(0.00) | 9 | 0.00 |
| Doxycycline | J01AA | 0(0.00) | 3(1.07) | 3(1.07) | 241(85.77) | 32(11.39) | 113(40.21) | 4(1.42) | 281 | 1.07 |
| Tigecycline | J01AA | 0(0.00) | 25(14.12) | 5(2.82) | 109(61.58) | 16(9.04) | 96(54.24) | 1(0.56) | 177 | 14.12(8.99,19.26) |
| Cefpodoxime | J01DD | 0(0.00) | 0(0.00) | 0(0.00) | 8(88.89) | 0(0.00) | 1(11.11) | 0(0.00) | 9 | 0.00 |
| Metronidazole | J01XD | 1(0.56) | 10(5.59) | 3(1.68) | 129(72.07) | 12(6.70) | 96(53.63) | 1(0.56) | 179 | 5.59(2.22,8.95) |
| Tinidazole | J01XD | 0(0.00) | 0(0.00) | 0(0.00) | 4(100.00) | 0(0.00) | 1(25.00) | 0(0.00) | 4 | 0.00 |
| Linezolid | J01XX | 0(0.00) | 12(9.45) | 1(0.79) | 68(53.54) | 6(4.72) | 75(59.06) | 0(0.00) | 127 | 9.45(4.36,14.54) |
| Rifampicin | J04AB | 0(0.00) | 1(1.69) | 1(1.69) | 43(72.88) | 6(10.17) | 24(40.68) | 0(0.00) | 59 | 1.69 |
| Isoniazid | J04AC | 1(1.45) | 5(7.25) | 1(1.45) | 39(56.52) | 10(14.49) | 39(56.52) | 0(0.00) | 69 | 7.25 |
| Bedaquiline | J04AK | 0(0.00) | 7(30.43) | 0(0.00) | 9(39.13) | 2(8.70) | 14(60.87) | 0(0.00) | 23 | 30.43(11.63,49.24) |
| Pyrazinamide | J04AK | 0(0.00) | 3(27.27) | 0(0.00) | 10(90.91) | 4(36.36) | 3(27.27) | 0(0.00) | 11 | 27.27 |
| Foscarnet | J05AD | 0(0.00) | 2(18.18) | 2(18.18) | 4(36.36) | 2(18.18) | 9(81.82) | 0(0.00) | 11 | 18.18 |
| Atazanavir | J05AE | 0(0.00) | 7(12.28) | 0(0.00) | 38(66.67) | 4(7.02) | 27(47.37) | 1(1.75) | 57 | 12.28(3.76,20.80) |
| Fosamprenavir | J05AE | 0(0.00) | 0(0.00) | 0(0.00) | 9(90.00) | 0(0.00) | 1(10.00) | 0(0.00) | 10 | 0.00 |
| Indinavir | J05AE | 0(0.00) | 1(12.50) | 1(12.50) | 6(75.00) | 1(12.50) | 4(50.00) | 0(0.00) | 8 | 12.50 |
| Abacavir | J05AF | 0(0.00) | 9(9.00) | 3(3.00) | 62(62.00) | 11(11.00) | 65(65.00) | 1(1.00) | 100 | 9.00(3.39,14.61) |
| Didanosine | J05AF | 0(0.00) | 11(21.57) | 0(0.00) | 24(47.06) | 4(7.84) | 33(64.71) | 2(3.92) | 51 | 21.57(10.28,32.86) |
| Stavudine | J05AF | 0(0.00) | 24(47.06) | 2(3.92) | 27(52.94) | 6(11.76) | 28(54.90) | 0(0.00) | 51 | 47.06(33.36,60.76) |
| Nevirapine | J05AG | 0(0.00) | 11(16.67) | 0(0.00) | 26(39.39) | 2(3.03) | 36(54.55) | 0(0.00) | 66 | 16.67(7.68,25.66) |
| Raltegravir | J05AJ | 0(0.00) | 6(9.23) | 3(4.62) | 50(76.92) | 9(13.85) | 32(49.23) | 0(0.00) | 65 | 9.23(2.19,16.27) |
| Enfuvirtide | J05AX | 0(0.00) | 2(7.41) | 2(7.41) | 22(81.48) | 3(11.11) | 4(14.81) | 0(0.00) | 27 | 7.41 |
| Clofarabine | L01BB | 0(0.00) | 8(19.51) | 2(4.88) | 39(95.12) | 18(43.90) | 10(24.39) | 2(4.88) | 41 | 19.51(7.38,31.64) |
| Mercaptopurine | L01BB | 0(0.00) | 4(3.13) | 1(0.78) | 81(63.28) | 7(5.47) | 58(45.31) | 2(1.56) | 128 | 3.13 |
| Tioguanine | L01BB | 0(0.00) | 0(0.00) | 0(0.00) | 3(50.00) | 1(16.67) | 4(66.67) | 0(0.00) | 6 | 0.00 |
| Vincristine | L01CA | 0(0.00) | 2(2.82) | 3(4.23) | 48(67.61) | 12(16.90) | 36(50.70) | 0(0.00) | 71 | 2.82 |
| Daunorubicin | L01DB | 0(0.00) | 6(22.22) | 0(0.00) | 21(77.78) | 0(0.00) | 10(37.04) | 1(3.70) | 27 | 22.22(6.54,37.90) |
| Lenvatinib | L01EX | 0(0.00) | 8(11.43) | 0(0.00) | 65(92.86) | 6(8.57) | 9(12.86) | 0(0.00) | 70 | 11.43(3.98,18.88) |
| Brentuximab vedotin | L01FX | 0(0.00) | 13(14.61) | 5(5.62) | 61(68.54) | 18(20.22) | 67(75.28) | 4(4.49) | 89 | 14.61(7.27,21.94) |
| Nilotinib | L01XE | 0(0.00) | 9(2.06) | 1(0.23) | 208(47.60) | 15(3.43) | 281(64.30) | 1(0.23) | 437 | 2.06(0.73,3.39) |
| Ponatinib | L01XE | 0(0.00) | 30(16.48) | 0(0.00) | 127(69.78) | 3(1.65) | 153(84.07) | 0(0.00) | 182 | 16.48(11.09,21.87) |
| Arsenic trioxide | L01XX | 0(0.00) | 1(7.14) | 0(0.00) | 11(78.57) | 1(7.14) | 6(42.86) | 0(0.00) | 14 | 7.14 |
| Asparaginase | L01XX | 0(0.00) | 14(8.64) | 3(1.85) | 73(45.06) | 11(6.79) | 109(67.28) | 2(1.23) | 162 | 8.64(4.32,12.97) |
| Pegaspargase | L01XX | 0(0.00) | 31(11.79) | 9(3.42) | 211(80.23) | 55(20.91) | 96(36.50) | 3(1.14) | 263 | 11.79(7.89,15.68) |
| Basiliximab | L04AC | 0(0.00) | 2(9.52) | 0(0.00) | 12(57.14) | 1(4.76) | 15(71.43) | 0(0.00) | 21 | 9.52 |
| Azathioprine | L04AX | 0(0.00) | 3(1.30) | 3(1.30) | 156(67.83) | 12(5.22) | 119(51.74) | 3(1.30) | 230 | 1.30 |
| Flurbiprofen | M01AE | 0(0.00) | 0(0.00) | 0(0.00) | 5(100.00) | 0(0.00) | 0(0.00) | 0(0.00) | 5 | 0.00 |
| Ketoprofen | M01AE | 0(0.00) | 0(0.00) | 0(0.00) | 16(94.12) | 0(0.00) | 3(17.65) | 0(0.00) | 17 | 0.00 |
| Propofol | N01AX | 0(0.00) | 14(15.38) | 5(5.49) | 57(62.64) | 21(23.08) | 34(37.36) | 5(5.49) | 91 | 15.38(7.97,22.80) |
| Codeine | N02AA | 0(0.00) | 7(9.09) | 0(0.00) | 61(79.22) | 3(3.90) | 33(42.86) | 0(0.00) | 77 | 9.09(2.67,15.51) |
| Eslicarbazepine | N03AF | 0(0.00) | 1(12.50) | 0(0.00) | 7(87.50) | 1(12.50) | 5(62.50) | 0(0.00) | 8 | 12.50 |
| Rufinamide | N03AF | 0(0.00) | 0(0.00) | 1(16.67) | 6(100.00) | 0(0.00) | 0(0.00) | 0(0.00) | 6 | 0.00 |
| Valproic acid | N03AG | 1(0.24) | 57(13.41) | 13(3.06) | 334(78.59) | 54(12.71) | 161(37.88) | 25(5.88) | 425 | 13.41(10.17,16.65) |
| Fluphenazine | N05AB | 0(0.00) | 6(85.71) | 0(0.00) | 4(57.14) | 0(0.00) | 3(42.86) | 0(0.00) | 7 | 85.71 |
| Olanzapine | N05AH | 0(0.00) | 104(7.13) | 16(1.10) | 712(48.80) | 66(4.52) | 1214(83.21) | 8(0.55) | 1459 | 7.13(5.81,8.45) |
| Quetiapine | N05AH | 0(0.00) | 322(9.34) | 42(1.22) | 739(21.43) | 75(2.18) | 3119(90.46) | 18(0.52) | 3448 | 9.34(8.37,10.31) |
| Meprobamate | N05BC | 0(0.00) | 0(0.00) | 0(0.00) | 3(100.00) | 1(33.33) | 2(66.67) | 0(0.00) | 3 | 0.00 |
| Riluzole | N07XX | 0(0.00) | 11(20.75) | 0(0.00) | 35(66.04) | 7(13.21) | 36(67.92) | 0(0.00) | 53 | 20.75(9.84,31.67) |
| Miltefosine | P01CX | 0(0.00) | 0(0.00) | 0(0.00) | 0(0.00) | 0(0.00) | 3(100.00) | 0(0.00) | 3 | 0.00 |
| Pentamidine isethionate | P01CX | 0(0.00) | 1(16.67) | 0(0.00) | 3(50.00) | 0(0.00) | 2(33.33) | 0(0.00) | 6 | 16.67 |
| Calcium acetate | V03AE | 0(0.00) | 0(0.00) | 0(0.00) | 8(88.89) | 0(0.00) | 5(55.56) | 0(0.00) | 9 | 0.00 |
| Secretin | V04CK | 0(0.00) | 0(0.00) | 0(0.00) | 4(100.00) | 0(0.00) | 0(0.00) | 0(0.00) | 4 | 0.00 |
| Iodixanol | V08AB | 0(0.00) | 2(5.71) | 0(0.00) | 31(88.57) | 1(2.86) | 4(11.43) | 1(2.86) | 35 | 5.71 |
| Iothalamic acid | V09CX | 0(0.00) | 0(0.00) | 0(0.00) | 0(0.00) | 0(0.00) | 7(100.00) | 0(0.00) | 7 | 0.00 |

*The 95% two-sided confidence interval was calculated using the normal approximation method for drugs with both death and non-death reports greater than 5。

Abbreviations: ATC, Anatomical Therapeutic Chemical; CI, confidence interval.

Supplemental table 7. Full names of potential target genes of drug-induced pancreatitis.

| Target genes | Full names | Target genes | Full names |
| --- | --- | --- | --- |
| AKT1 | AKT serine/threonine kinase 1 | KCNJ11 | Potassium inwardly rectifying channel subfamily J member 11 |
| ATM | ATM serine/threonine kinase | KRAS | KRAS proto-oncogene, GTPase |
| BAX | BCL2 associated X, apoptosis regulator | LEPR | Leptin receptor |
| BCL2 | BCL2 apoptosis regulator | LPL | Lipoprotein lipase |
| BDKRB2 | Bradykinin receptor B2 | MAPK1 | Mitogen-activated protein kinase 1 |
| C11orf65 | Chromosome 11 open reading frame 65 | MEN1 | Menin 1 |
| CAV1 | Caveolin 1 | MET | MET proto-oncogene, receptor tyrosine kinase |
| CCKBR | Cholecystokinin B receptor | MMP1 | Matrix metallopeptidase 1 |
| CCND1 | Cyclin D1 | MMP2 | Matrix metallopeptidase 2 |
| CDKN1A | Cyclin dependent kinase inhibitor 1A | MPO | Myeloperoxidase |
| CDKN1B | Cyclin dependent kinase inhibitor 1B | MTOR | Mechanistic target of rapamycin kinase |
| CEL | Carboxyl ester lipase | MYC | MYC proto-oncogene, bHLH transcription factor |
| CNR1 | Cannabinoid receptor 1 | NEUROD1 | Neuronal differentiation 1 |
| CPA2 | Carboxypeptidase A2 | NOTCH1 | Notch receptor 1 |
| CRP | C-reactive protein | PAX4 | Paired box 4 |
| CTLA4 | Cytotoxic T-lymphocyte associated protein 4 | PIK3CA | Phosphatidylinositol-4,5-bisphosphate 3-kinase catalytic subunit alpha |
| CXCL12 | C-X-C motif chemokine ligand 12 | PLA2G1B | Phospholipase A2 group IB |
| CXCL8 | C-X-C motif chemokine ligand 8 | PLG | Plasminogen |
| EGFR | Epidermal growth factor receptor | POLD1 | DNA polymerase delta 1, catalytic subunit |
| ERBB2 | Erb-b2 receptor tyrosine kinase 2 | PPARG | Peroxisome proliferator activated receptor gamma |
| FAS | Fas cell surface death receptor | PTEN | phosphatase and tensin homolog |
| FOS | Fos proto-oncogene, AP-1 transcription factor subunit | PTGS2 | Prostaglandin-endoperoxide synthase 2 |
| GCG | Glucagon | RET | Ret proto-oncogene |
| HGF | Hepatocyte growth factor | SCTR | Secretin receptor |
| HLA-B | Major histocompatibility complex, class I, B | SMAD3 | SMAD family member 3 |
| HLA-DQA1 | Major histocompatibility complex, class II, DQ alpha 1 | SP1 | Sp1 Ttanscription factor |
| HLA-DRB1 | Major histocompatibility complex, class II, DR beta 1 | SRC | SRC proto-oncogene, non-receptor tyrosine kinase |
| HNF1B | HNF1 homeobox B | STK11 | Serine/threonine kinase 11 |
| HRAS | HRas proto-oncogene, GTPase | TLR4 | Toll like receptor 4 |
| HTR2A | 5-hydroxytryptamine receptor 2A | TNF | Tumor necrosis factor |
| IFNG | Interferon gamma | TP53 | Tumor protein p53 |
| IKZF1 | IKAROS family zinc finger 1 | TRPV1 | Transient receptor potential cation channel subfamily V member 1 |
| IL10 | Interleukin 10 | VEGFA | Vascular endothelial growth factor A |
| IL1B | Interleukin 1 beta | VIP | Vasoactive intestinal peptide |
| IL6 | Interleukin 6 | XDH | Xanthine dehydrogenase |
